# Design and synthesis of 4-piperazinyl quinoline derived urea/thioureas for anti-breast cancer activity by a hybrid pharmacophore approach

**DOI:** 10.1080/14756366.2019.1571055

**Published:** 2019-02-06

**Authors:** Raja Solomon Viswas, Sheetal Pundir, Hoyun Lee

**Affiliations:** a Health Sciences North Research Institute, Sudbury, Canada;; b Department of Medicine, The University of Ottawa, Ottawa, Canada

**Keywords:** 4-Aminoquinoline, anticancer agents, urea, thiourea, repurposing, pharmacophore approach

## Abstract

In an attempt to improve anti-breast cancer activity, a new series of 4-piperazinylquinoline derivatives based on the urea/thiourea scaffold were designed and synthesised by a pharmacophore hybrid approach. We then examined for their antiproliferative effects on three human breast tumor cell lines, MDA-MB231, MDA-MB468 and MCF7, and two non-cancer breast epithelial cell lines, 184B5 and MCF10A. Among those 26 novel compounds examined, **5**, **9, 17**, **18**, **21**, **23** and **29** showed significantly improved antiproliferative activity on breast cancer cells. Compound **23** (4-(7-chloro-quinolin-4-yl)-piperazine-1-carbothioic acid (2-morpholin-4-yl-ethyl)-amide) (**RL-15**) is especially desirable, since its antigrowth/cell-killing activity is 7-11 fold higher on cancer than non-cancer cells. Data from cell biological studies demonstrated that cancer cells compromised plasma membrane integrity in the presence of compound **23**. The cancer cell-specific property of compound **23** shown in cell culture stands *in vivo* test, this compound can be an excellent lead for effective and safe anticancer drug.

## Introduction

**Figure 1.**
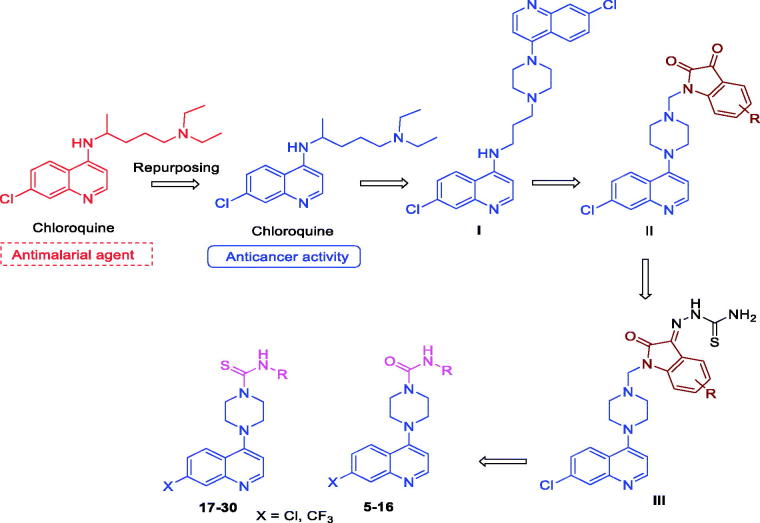
The structures of 4-aminoquinolines reported for anticancer activity reported from this laboratory by a pharmacophore hybridization approach.

Chloroquine (CQ), which contains a 4-aminoquinoline scaffold (Figure 1), is a well-known antimalarial drug. Based on repurposing concept, we previously demonstrated that the combination of CQ with radiation or Akt inhibitors not only significantly increases antigrowth/cell-killing effects but also enhances the selectivity towards cancer over non-cancer cells^1–3^. CQ is well known for their lysosomotropic property and accrued in the lysosomes and elevates intra-lysosomal pH; and inhibits with autophagosome degradation in the lysosomes. This unique characteristic of CQ and its analogs may be imperative for the enhancement of cell-killing by cancer therapeutic agents in different tumor models^4^^,^^5^. Based on this interesting note, we synthesised several 4-aminoquinoline analogs (Figure 1, **I**) and examined their cytotoxic effects on breast cancer cell lines. We found that some of these compounds are very effective and show selective cytotoxic effects on cancer cells^6^^,^^7^. In continuation of our efforts to develop more effective CQ analogs (Figure 1, **II** and **III**) by merging 4-piperazinylquinoline ring structure with an isatin ring by a hybrid approach, and found that 4-piperazinylquinoline exhibited promising anti-breast cancer activity^8–10^.

**Figure 2.**
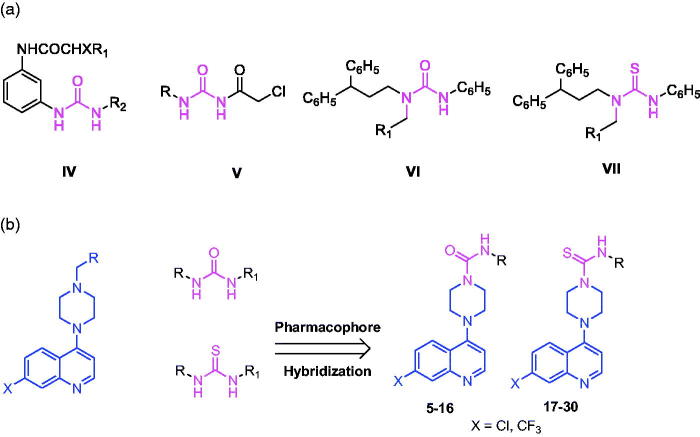
(a) Urea and thiourea analogs with anticancer activity. (b) The design and synthesis of hybrid 4-piperazinylquinoline analogs.

Small heterocyclic molecules have enrich potential for the discovery of drug candidates, among which urea and thiourea groups are privileged pharmacophores found in many medicinally active compounds. They have been shown to have promise against cancer cells, HIV-1 protease, hypercholesteromia and atherosclerosis^11^^,^^12^. The current literatures evident that molecules containing urea and thiourea pharmacophores (Figure 2(a), **IV–VII**) are potent inhibitors of human DNA-topoisomerase II and active against various cancer cells^11^^,^^13–18^. Based on these prior annotations, we surmised that appropriate hybridization on these pharmacophores (i.e. 4-piperazinylquinoline and urea/thiourea) could possess an effective anticancer activity. Similar approaches of combining two molecules have previously been exploited with very good results^19–24^. Encouraged by these results, we designed and synthesised hybrid compounds by linking the main structural unit of the 4-piperazinylquinoline ring system with the urea/thiourea functionality, and examined their cytotoxic effects on three human breast tumor and two matching non-cancer cell lines (Figure 2(b), Scheme 1). For the most desirable compound, we carried out cell-based experiments to gain the mechanism of function.

**Figure:**
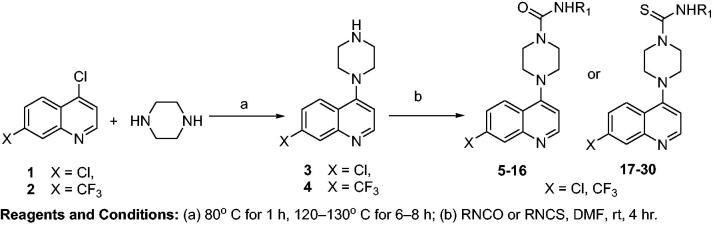
**Scheme 1**. Synthesis of 4-piperazinyl quinoline derived urea (**5–16**) and thiourea (**17–30**) analogues.

## Materials and methods

### Chemistry

Melting points (mp) were taken in open capillaries on the Complab melting point apparatus. Elemental analysis was performed on a Perkin-Elmer 2400 C, H, N analyzer and values were within the acceptable limits of the calculated values. The ^1^H NMR spectra were recorded on a DPX-500 MHz Bruker FT-NMR spectrometer using deuterated chloroform (CDCl_3_) and dimethyl sulfoxide (DMSO)-d_6_ as solvent. The chemical shifts were reported as parts per million (δ ppm) tetramethylsilane (TMS) as an internal standard. Mass spectra were obtained on a JEOL-SX-102 instrument using fast atom bombardment (FAB positive). The progress of the reaction was monitored on readymade silica-gel plates (Merck) using chloroform-methanol (9:1) as solvent. Iodine was used as a developing agent or by spraying with the Dragendorff’s reagent. Chromatographic purification was performed over a silica gel (100–200 mesh). All chemicals and reagents obtained from Sigma-Aldrich (USA) were used without further purification.

### General synthetic procedure for 7-substituted-4-piperazin-1-yl-quinoline

A mixture of 7-substituted-4-chloro-quinoline (10.10 mmol), piperazine (2.61 g, 30.30 mmol) and triethylamine (1.4 ml, 10.10 mmol) were heated slowly to 80 °C over 1 h while stirring. The temperature was then increased to 130-140 °C for 6 h where it was kept for while stirring continuously. The reaction mixture was taken up in dichloromethane after cooled to room temperature. The organic layer was washed with 5% aq. NaHCO_3_, followed by washing with water and then with brine. The organic layer was dried over anhydrous Na_2_SO_4_ and solvent was removed under reduced pressure, and the residue was then precipitated by addition of mixture of solvent hexane: chloroform (8:2).

### 7 -Chloro-4-piperazin-1-yl-quinoline (3)

This compound was obtained as a pale white solid in 86% yield; ^1^H NMR (500 MHz, CDCl_3_): δ 2.31 (br s, 1H, N*H*), 3.15 (s, 4H, N(C*H*_2_CH_2_)_2_NAr), 3.18 (s, 4H, N(CH_2_C*H*_2_)_2_NAr), 6.80–6.81 (d, *J* = 5.0 Hz, 1H, Ar–*H*), 7.46–7.47 (d, *J* = 5.0 Hz, 1H, Ar–*H*), 7.92–7.94 (d, *J* = 10.0 Hz, 1H, Ar–*H*), 8.01 (s, 1H, Ar–*H*), 8.68–8.69 (d, *J* = 5.0 Hz, 1H, Ar–*H*); ^13^C NMR (500 MHz, CDCl_3_): δ 46.10 (2C), 53.58 (2C), 108.97, 121.97, 125.24, 126.09, 128.91, 134.84, 150.22, 151.99, 157.38; electron spray mass spectroscopy (ES-MS) *m/z* 248 [M + H]^+^; Anal.Calcd for C_13_H_14_ClN_3_: C, 63.03; H, 5.70; N, 16.96; found: C, 63.01; H, 5.73; N, 16.99.

### 4 -Piperazin-1-yl-7-trifluoromethyl-quinoline (4)

This compound was obtained as a pale yellowish white solid in 78% yield; ^1^H NMR (500 MHz, CDCl_3_): δ 1.78 (br s, 1H, N*H*), 3.18 (s, 4H, N(C*H*_2_CH_2_)_2_NAr), 3.24 (s, 4H, N(CH_2_C*H*_2_)_2_NAr), 7.07–7.08 (d, *J* = 5.0 Hz, 1H, Ar–*H*), 7.46–7.47 (d, *J* = 5.0 Hz, 1H, Ar–*H*), 7.63–7.65 (d, *J* = 10.0 Hz, 1H, Ar–*H*), 8.13–8.14 (d, *J* = 5.0 Hz, 1H, Ar–*H*), 8.34 (s, 1H, Ar–*H*), 8.81–8.82 (d, *J* = 5.0 Hz, 1H, Ar–*H*); ^13^C NMR (500 MHz, CDCl_3_): δ 52.15 (2C), 53.48 (2C), 110.64, 120.79, 125.14, 125.22, 127.76, 130.70, 130.96, 148.73, 152.20, 157.19; ES-MS *m/z* 282 [M + H]^+^; Anal.Calcd for C_14_H_14_F_3_N_3_: C, 59.78; H, 5.02; N, 14.94; found: C, 59.75; H, 4.98; N, 14.97.

### General synthetic procedure for urea (1–2) and thiourea analogs of 4-aminoquinoline (5–30)

A mixture 7-substituted-4-piperazin-1-yl-quinoline (3.33 mmol), triethylamine (0.5 ml, 3.33 mmol) and appropriate isocynate or isothiocynate (3.33 mmol) in anhydrous DMF were stirred at room temperature until the reaction was complete. This reaction mixture was poured into statured NaCl solution and products get precipitated out. In all cases, the desired urea or thiourea product precipitated from NaCl solution. The precipitate was collected via vacuum filtration and dried *in vacuo* and recrystallised from mixture of solvent hexane: dichloromethane (3:7).

### 4 -(7-Chloro-quinolin-4-yl)-piperazine-1-carboxylic acid phenylamide (5)

This compound was obtained as a pale yellowish white solid in 98% yield; M.p: 142–143 °C; IR (Potassium bromide, KBr) ν_max_ C=O 1645 cm^−1^; ^1^H NMR (500 MHz, CDCl_3_): δ 3.29 (s, 4H, N(CH_2_C*H*_2_)_2_NAr), 3.79 (s, 4H, N(C*H*_2_CH_2_)_2_NAr), 6.63 (br s, 1H, N*H*), 6.87–6.88 (d, *J* = 5.0 Hz, 1H, Ar–*H*), 7.08–7.09 (d, *J* = 5.0 Hz, 1H, Ar–*H*), 7.32–7.33 (d, *J* = 5.0 Hz, 1H, Ar–*H*), 7.29 (s, 1H, Ar–*H*), 7.35–7.37 (d, *J* = 10.0 Hz, 1H, Ar–*H*), 7.39–7.41 (d, *J* = 10.0 Hz, 1H, Ar–*H*), 7.48–7.50 (d, *J* = 10.0 Hz, 1H, Ar–*H*), 7.99–8.01 (d, *J* = 10.0 Hz, 1H, Ar–*H*), 8.03 (s, 1H, Ar–*H*), 8.78–8.79 (d, *J* = 5.0 Hz, 1H, Ar–*H*); ^13^C NMR (125 MHz, CDCl_3_): δ 44.21 (2C), 52.20 (2C), 109.58, 118.53, 120.41, 121.92, 122.04, 122.40, 125.61, 126.24, 128.48, 128.64, 134.54, 140.37, 150.10, 152.12, 155.71, 156.65; ES-MS *m/z* 366 [M + H]^+^; Anal.Calcd for C_20_H_19_ClN_4_O: C, 65.48; H, 5.22; N, 15.27; found: C, 65.45; H, 5.27; N, 15.22.

### 4 -(7-Chloro-quinolin-4-yl)-piperazine-1-carboxylic acid (2,5-dimethyl-phenyl)-amide (6)

This compound was obtained as a yellowish pale white solid in 76% yield; M.p: 118–119 °C; IR (KBr) ν_max_ C = O 1638 cm^−1^; ^1^H NMR (500 MHz, CDCl_3_): δ 2.16 (m, 3H, C*H*_3_), 2.36 (m, 3H, C*H*_3_), 3.34 (s, 4H, N(CH_2_C*H*_2_)_2_NAr), 3.74 (s, 4H, N(C*H*_2_CH_2_)_2_NAr), 6.17 (br s, 1H, N*H*), 6.81–6.82 (d, *J* = 5.0 Hz, 1H, Ar–*H*), 6.96–6.97 (d, *J* = 5.0 Hz, 1H, Ar–*H*), 6.99–7.01 (d, *J* = 10.0 Hz, 1H, Ar–*H*), 7.11 7.61–7.64 (m, 2H, Ar–*H*), 8.14–8.16 (d, *J* = 10.0 Hz, 1H, Ar–*H*), 8.25 (s, 1H, Ar–*H*), 8.76–8.77 (d, *J* = 5.0 Hz, 1H, Ar–*H*); ^13^C NMR (125 MHz, CDCl_3_): δ 15.53, 25.23, 48.38 (2C), 51.61 (2C), 109.25, 121.73, 124.78, 127.12, 127.74, 129.27 (4C), 129.34, 135.81, 136.78, 137.12, 150.21, 151.92, 156.06; ES-MS *m/z* 395 [M + H]^+^; Anal.Calcd for C_22_H_23_ClN_4_O: C, 66.91; H, 5.87; N, 14.19; found: C, 66.96; H, 5.91; N, 14.15.

### 4 -(7-Chloro-quinolin-4-yl)-piperazine-1-carboxylic acid (4-trifluoromethyl-phenyl)-amide (7)

This compound was obtained as a pale yellowish white solid in 69% yield; M.p: 175–176 °C; IR (KBr) ν_max_ C = O 1625 cm^−1^; ^1^H NMR (500 MHz, CDCl_3_): δ 3.29 (s, 4H, N(C*H*_2_CH_2_)_2_NAr), 3.83 (s, 4H, N(CH_2_C*H*_2_)_2_NAr), 6.87–6.88 (d, *J* = 5.0 Hz, 1H, Ar–*H*), 6.97 (s, 1H, Ar–*H*), 7.48–7.50 (d, *J* = 10.0 Hz, 1H, Ar–*H*), 7.50–7.54 (m, 4H, Ar–*H*), 7.96–7.98 (d, *J* = 10.0 Hz, 1H, Ar–*H*), 8.09–8.10 (d, *J* = 5.0 Hz, 1H, Ar–*H*), 8.80 (br s, 1H, N*H*); ^13^C NMR (125 MHz, CDCl_3_): δ 44.22 (2C), 51.91 (2C), 119.31 (3C), 121.75, 124.77, 125.30, 126.19, 126.12, 126.22, 128.89, 135.39, 142.09, 149.98, 151.78, 151.78, 154.53, 156.44; ES-MS *m/z* 435 [M + H]^+^; Anal.Calcd for C_21_H_18_ClF_3_N_4_O: C, 58.00; H, 4.17; N, 12.88; found: C, 57.96; H, 4.21; N, 12.92.

### 4 -(7-Chloro-quinolin-4-yl)-piperazine-1-carboxylic acid (2,4,6-trichloro-phenyl)-amide (8)

This compound was obtained as a pale white solid in 76% yield; M.p: 168–169 °C; IR (KBr) ν_max_ C = O 1627 cm^−1^; ^1^H NMR (500 MHz, CDCl_3_): δ 3.27 (s, 4H, N(CH_2_C*H*_2_)_2_NAr), 3.79 (s, 4H, N(C*H*_2_CH_2_)_2_NAr), 6.95–6.96 (d, *J* = 5.0 Hz, 1H, Ar–*H*), 7.47 (s, 2H, Ar–*H*), 7.49 (br s, 1H, N*H*), 7.93 (s, 1H, Ar–*H*), 8.05–8.07 (d, *J* = 10.0 Hz, 1H, Ar–*H*), 8.53 (s, 1H, Ar–*H*), 8.68–8.69 (d, *J* = 5.0 Hz, 1H, Ar–*H*); ^13^C NMR (500 MHz, CDCl_3_): δ 44.64 (2C), 52.24 (2C), 110.72, 121.44, 124.85, 125.34, 128.05 (2C), 128.41 (2C), 133.25, 132.28, 132.45, 133.19, 148.76, 152.24, 154.39, 156.55; ES-MS *m/z* 471 [M + H]^+^; Anal.Calcd for C_20_H_16_Cl_4_N_4_O: C, 51.09; H, 3.43; N, 11.92; found: C, 51.13; H, 3.47; N, 11.96.

### 4 -(7-Chloro-quinolin-4-yl)-piperazine-1-carboxylic acid naphthalen-1-ylamide (9)

This compound was obtained as a pale creamy white solid in 72% yield; M.p: 138–139 °C; IR (KBr) ν_max_ C = O 1622 cm^−1^; ^1^H NMR (500 MHz, CDCl_3_): δ 3.29 (s, 4H, N(CH_2_C*H*_2_)_2_NAr), 3.86 (s, 4H, N(C*H*_2_CH_2_)_2_NAr), 6.92 (br s, 1H, N*H*), 7.39–7.40 (d, *J* = 5.0 Hz, 1H, Ar–*H*), 7.43–7.45 (d, *J* = 10.0 Hz, 1H, Ar–*H*), 7.53–7.55 (d, *J* = 10.0 Hz, 1H, Ar–*H*), 7.63–7.65 (d, *J* = 10.0 Hz, 1H, Ar–*H*), 7.68–7.75 (m, 4H, Ar–*H*), 7.93–8.03 (m, 2H, Ar–*H*), 8.53 (s, 1H, Ar–*H*), 8.70–8.71 (d, *J* = 5.0 Hz, 1H, Ar–*H*); ^13^C NMR (125 MHz, CDCl_3_): δ 44.23 (2C), 52.18 (2C), 110.71, 120.93, 123.14, 123.24, 125.24, 125.59, 125.62, 125.79 (2C), 125.91 (2C), 127.66, 128.18, 129.79, 130.81, 133.22, 134.93, 148.75, 152.21, 156.64, 156.77; ES-MS *m/z* 417 [M + H]^+^; Anal.Calcd for C_24_H_21_ClN_4_O: C, 69.14; H, 5.08; N, 13.44; found: C, 69.17; H, 5.05; N, 13.39.

### 4 -(7-Chloro-quinolin-4-yl)-piperazine-1-carboxylic acid cyclohexylamide (10)

This compound was obtained as a pale creamy white solid in 68% yield; M.p: 112–113 °C; IR (KBr) ν_max_ C = O 1620 cm^−1^; ^1^H NMR (500 MHz, CDCl_3_): δ 1.74 (m, 6H, C*H*_2_ cyclohexyl), 1.96–2.02 (m, 4H, C*H*_2_ cyclohexyl), 3.23 (s, 4H, N(CH_2_C*H*_2_)_2_NAr), 3.62 (s, 4H, N(C*H*_2_CH_2_)_2_NAr), 3.65–3.69 (m, 1H, C*H* cyclohexyl), 4.40 (br s, 1H, N*H*), 6.86–6.87 (d, *J* = 5.0 Hz, 1H, Ar–*H*), 7.47–7.48 (d, *J* = 5.0 Hz, 1H, Ar–*H*), 7.96–7.98 (d, *J* = 10.0 Hz, 1H, Ar–*H*), 8.08 (s, 1H, Ar–*H*), 8.81–8.82 (d, *J* = 5.0 Hz, 1H, Ar–*H*); ^13^C NMR (125 MHz, CDCl_3_): δ 20.34 (2C), 26.84, 31.56 (2C), 44.21 (2C), 47.67, 52.20 (2C), 109.58, 118.53, 121.92, 122.04, 126.24, 128.48, 140.37, 150.10, 153.12, 156.65; ES-MS *m/z* 373 [M + H]^+^; Anal.Calcd for C_20_H_25_ClN_4_O: C, 64.42; H, 6.76; N, 15.03; found: C, 64.40; H, 6.72; N, 15.07.

### 4 -(7-Trifluoromethyl-quinolin-4-yl)-piperazine-1-carboxylic acid phenylamide (11)

This compound was obtained as a pale yellowish white solid in 92% yield; M.p: 146–147 °C; IR (KBr) ν_max_ C = O 1640 cm^−1^; ^1^H NMR (500 MHz, CDCl_3_): δ 3.31 (s, 4H, N(CH_2_C*H*_2_)_2_NAr), 3.84 (s, 4H, N(C*H*_2_CH_2_)_2_NAr), 6.76 (br s, 1H, N*H*), 6.78–6.79 (d, *J* = 5.0 Hz, 1H, Ar–*H*), 7.09–7.10 (d, *J* = 5.0 Hz, 1H, Ar–*H*), 7.29–7.33 (m, 2H, Ar–*H*), 7.34–7.40 (m, 2H, Ar–*H*), 7.70–7.72 (d, *J* = 10.0 Hz, 1H, Ar–*H*), 8.15–8.17 (d, *J* = 10.0 Hz, 1H, Ar–*H*), 8.39 (s, 1H, Ar–*H*), 8.85–8.86 (d, *J* = 5.0 Hz, 1H, Ar–*H*); ^13^C NMR (125 MHz, CDCl_3_): δ 44.32 (2C), 51.90 (2C), 109.48, 118.63, 120.56, 121.89, 122.04, 122.53, 124.35, 125.61, 126.24, 128.48, 128.64, 132.34, 140.57, 150.18, 152.17, 155.71, 156.58; ES-MS *m/z* 400 [M + H]^+^; Anal.Calcd for C_21_H_19_F_3_N_4_O: C, 62.99; H, 4.78; N, 13.99; found: C, 63.02; H, 4.82; N, 14.05.

### 4 -(7-Trifluoromethyl-quinolin-4-yl)-piperazine-1-carboxylic acid (2,5-dimethyl-phenyl)-amide (12)

This compound was obtained as a pale yellowish white solid in 74% yield; M.p: 108–109 °C; IR (KBr) ν_max_ C = O 1633 cm^−1^; ^1^H NMR (500 MHz, CDCl_3_): δ 2.15 (m, 3H, C*H*_3_), 2.22 (m, 3H, C*H*_3_), 3.28 (s, 4H, N(CH_2_C*H*_2_)_2_NAr), 3.77 (s, 4H, N(C*H*_2_CH_2_)_2_NAr), 6.81–6.82 (d, *J* = 5.0 Hz, 1H, Ar–*H*), 6.96–6.97 (d, *J* = 5.0 Hz, 1H, Ar–*H*), 6.99–7.01 (d, *J* = 10.0 Hz, 1H, Ar–*H*), 7.11 (br s, 1H, N*H*), 7.61–7.64 (m, 2H, Ar–*H*), 8.14–8.16 (d, *J* = 10.0 Hz, 1H, Ar–*H*), 8.25 (s, 1H, Ar–*H*), 8.76–8.77 (d, *J* = 5.0 Hz, 1H, Ar–*H*); ^13^C NMR (125 MHz, CDCl_3_): δ 44.21 (2C), 52.12 (2C), 109.41, 118.47, 120.37, 121.77, 122.34, 122.56, 123.67, 124.31, 125.75, 126.24, 128.55, 128.67, 134.48, 140.37, 147.25, 150.10, 152.12, 155.71, 156.58; ES-MS *m/z* 429 [M + H]^+^; Anal.Calcd for C_23_H_23_F_3_N_4_O: C, 64.48; H, 5.41; N, 13.08; found: C, 64.42; H, 5.39; N, 13.12.

### 4 -(7-Trifluoromethyl-quinolin-4-yl)-piperazine-1-carboxylic acid (4-trifluoromethyl-phenyl)-amide (13)

This compound was obtained as a pale yellowish white solid in 73% yield; M.p: 154–155 °C; IR (KBr) ν_max_ C = O 1620 cm^−1^; ^1^H NMR (500 MHz, CDCl_3_): δ 3.31 (s, 4H, N(CH_2_C*H*_2_)_2_NAr), 3.84 (s, 4H, N(C*H*_2_CH_2_)_2_NAr), 6.69 (br s, 1H, N*H*), 6.98–6.99 (d, *J* = 5.0 Hz, 1H, Ar–*H*), 7.41–7.53 (m, 4H, Ar–*H*), 7.71–7.73 (d, *J* = 10.0 Hz, 1H, Ar–*H*), 8.16–8.18 (d, *J* = 10.0 Hz, 1H, Ar–*H*), 8.41 (s, 1H, Ar–*H*), 8.84–8.85 (d, *J* = 5.0 Hz, 1H, Ar–*H*); ^13^C NMR (125 MHz, CDCl_3_): δ 44.15 (2C), 51.78 (2C), 119.27 (3C), 121.65, 124.27, 124.65, 125.23, 126.25, 126.16, 126.24, 128.75, 135.41, 142.14, 149.87, 151.78, 151.78, 154.41, 156.35; ES-MS *m/z* 469 [M + H]^+^; Anal.Calcd for C_22_H_18_F_6_N_4_O: C, 56.41; H, 3.87; N, 11.96; found: C, 56.38; H, 3.91; N, 11.93.

### 4 -(7-Trifluoromethyl-quinolin-4-yl)-piperazine-1-carboxylic acid (2,4,6-trichloro-phenyl)-amide (14)

This compound was obtained as a pale yellowish white solid in 71% yield; M.p: 142–143 °C; IR (KBr) ν_max_ C = O 1622 cm^−1^; ^1^H NMR (500 MHz, CDCl_3_): δ 3.23 (s, 4H, N(CH_2_C*H*_2_)_2_NAr), 3.82 (s, 4H, N(C*H*_2_CH_2_)_2_NAr), 5.33 (br s, 1H, N*H*), 6.96–6.97 (d, *J* = 5.0 Hz, 1H, Ar–*H*), 7.33 (s, 2H, Ar–*H*), 7.62–7.64 (d, *J* = 10.0 Hz, 1H, Ar–*H*), 8.15–8.17 (d, *J* = 10.0 Hz, 1H, Ar–*H*), 8.22–8.24 (d, *J* = 10.0 Hz, 1H, Ar–*H*), 8.76–8.77 (d, *J* = 5.0 Hz, 1H, Ar–*H*); ^13^C NMR (125 MHz, CDCl_3_): δ 44.56 (2C), 52.01 (2C), 110.66, 121.33, 124.67 (2C), 125.11, 128.05 (2C), 128.41 (2C), 133.15, 132.20, 132.44, 133.15, 148.80, 152.24, 154.39, 156.25; ES-MS *m/z* 505 [M + H]^+^; Anal.Calcd for C_21_H_16_Cl_3_F_3_N_4_O: C, 50.07; H, 3.20; N, 11.12; found: C, 50.01; H, 3.25; N, 11.09.

### 4 -(7-Trifluoromethyl-quinolin-4-yl)-piperazine-1-carboxylic acid naphthalen-1-ylamide (15)

This compound was obtained as a pale yellowish white solid in 70% yield; M.p: 127–128 °C; IR (KBr) ν_max_ C = O 1623 cm^−1^; ^1^H NMR (500 MHz, CDCl_3_): δ 3.31 (s, 4H, N(CH_2_C*H*_2_)_2_NAr), 3.87 (s, 4H, N(C*H*_2_CH_2_)_2_NAr), 6.71 (br s, 1H, N*H*), 6.99–7.00 (d, *J* = 5.0 Hz, 1H, Ar–*H*), 7.49–7.58 (m, 3H, Ar–*H*), 7.69–7.73 (m, 3H, Ar–*H*), 7.85–7.94 (m, 2H, Ar–*H*), 8.14–8.16 (d, *J* = 10.0 Hz, 1H, Ar–*H*), 8.47 (s, 1H, Ar–*H*), 8.87–8.88 (d, *J* = 5.0 Hz, 1H, Ar–*H*); ^13^C NMR (125 MHz, CDCl_3_): δ 44.20 (2C), 52.14 (2C), 110.64, 120.93, 123.14, 123.24, 125.24, 125.59, 125.62, 125.79 (2C), 125.89 (2C), 127.66, 128.18, 129.77, 130.77, 134.19, 134.89, 148.66, 152.32, 156.54, 156.81; ES-MS *m/z* 451 [M + H]^+^; Anal.Calcd for C_25_H_21_F_3_N_4_O: C, 66.66; H, 4.70; N, 12.44; found: C, 66.62; H, 4.66; N, 12.41.

### 4 -(7-Trifluoromethyl-quinolin-4-yl)-piperazine-1-carboxylic acid cyclohexylamide (16)

This compound was obtained as a pale yellowish white solid in 60% yield; M.p: 132–133 °C; IR (KBr) ν_max_ C = O 1635 cm^−1^; ^1^H NMR (500 MHz, CDCl_3_): δ 1.73 (m, 6H, C*H*_2_ cyclohexyl), 1.96–2.02 (m, 4H, C*H*_2_ cyclohexyl), 3.24 (s, 4H, N(CH_2_C*H*_2_)_2_NAr), 3.67 (s, 4H, N(C*H*_2_CH_2_)_2_NAr), 3.69–3.72 (m, 1H, C*H* cyclohexyl), 4.42 (br s, 1H, N*H*), 6.96–6.97 (d, *J* = 5.0 Hz, 1H, Ar–*H*), 7.68–7.70 (d, *J* = 10.0 Hz, 1H, Ar–*H*), 8.15–8.16 (d, *J* = 5.0 Hz, 1H, Ar–*H*), 8.42 (s, 1H, Ar–*H*), 8.84–8.85 (d, *J* = 5.0 Hz, 1H, Ar–*H*); ^13^C NMR (125 MHz, CDCl_3_): δ 20.27 (2C), 26.74, 31.58 (2C), 44.24 (2C), 47.71, 52.24 (2C), 109.61, 118.55, 121.95, 122.41, 122.42, 126.41, 128.46, 140.33, 150.12, 153.14, 156.64; ES-MS *m/z* 406 [M + H]^+^; Anal.Calcd for C_21_H_25_F_3_N_4_O: C, 62.06; H, 6.20; N, 13.78; found: C, 62.10; H, 6.23; N, 13.82.

### 4 -(7-Chloro-quinolin-4-yl)-piperazine-1-carbothioic acid phenylamide (17)

This compound was obtained as a pale yellowish white solid in 98% yield; M.p: 171–172 °C; IR (KBr) ν_max_ C = S 1367 cm^−1^; ^1^H NMR (500 MHz, CDCl_3_): δ 3.37 (s, 4H, N(CH_2_C*H*_2_)_2_NAr), 4.17 (s, 4H, N(C*H*_2_CH_2_)_2_NAr), 6.86 (br s, 1H, N*H*), 7.16–7.29 (m, 3H, Ar–*H*), 7.45–7.50 (m, 2H, Ar–*H*), 7.59 (s, 1H, Ar–*H*), 7.93–7.95 (d, *J* = 10.0 Hz, 1H, Ar–*H*), 8.01 (s, 1H, Ar–*H*), 8.76–8.77 (d, *J* = 5.0 Hz, 1H, Ar–*H*); ^13^C NMR (125 MHz, CDCl_3_): δ 44.17 (2C), 52.14 (2C), 109.61, 118.56, 120.54, 121.91, 122.07, 122.45, 125.66, 126.27, 128.42, 128.66, 134.56, 140.39, 150.12, 151.84, 156.65, 182.89; ES-MS *m/z* 383 [M + H]^+^; Anal.Calcd for C_20_H_19_ClN_4_S: C, 62.73; H, 5.00; N, 14.63; found: C, 62.69; H, 4.97; N, 14.59.

### 4 -(7-Chloro-quinolin-4-yl)-piperazine-1-carbothioic acid (2,6-dimethyl-phenyl)-amide (18)

This compound was obtained as a pale yellowish white solid in 88% yield; M.p: 138–139 °C; IR (KBr) ν_max_ C = S 1355 cm^−1^; ^1^H NMR (500 MHz, CDCl_3_): δ 2.16 (m, 3H, C*H*_3_), 2.23 (m, 3H, C*H*_3_), 3.27 (s, 4H, N(CH_2_C*H*_2_)_2_NAr), 4.24 (s, 4H, N(C*H*_2_CH_2_)_2_NAr), 6.92–6.93 (d, *J* = 5.0 Hz, 1H, Ar–*H*), 7.03 (s, 3H, Ar–*H*), 7.44–7.46 (d, *J* = 10.0 Hz, 1H, Ar–*H*), 7.93 (s, 1H, Ar–*H*), 8.03–8.04 (d, *J* = 5.0 Hz, 1H, Ar–*H*), 8.67–8.68 (d, *J* = 5.0 Hz, 1H, Ar–*H*), 8.98 (br s, 1H, N*H*); ^13^C NMR (125 MHz, CDCl_3_): δ 18.53 (2C), 48.43 (2C), 51.68 (2C), 109.22, 121.71, 124.76, 126.66, 127.74, 128.57 (4C), 129.07, 135.78, 137.00, 150.16, 151.92, 156.06, 183.72; ES-MS *m/z* 411 [M + H]^+^; Anal.Calcd for C_22_H_23_ClN_4_S: C, 64.30; H, 5.64; N, 13.63; found: C, 64.26; H, 5.60; N, 13.59.

### 4 -(7-Chloro-quinolin-4-yl)-piperazine-1-carbothioic acid (4-trifluoromethyl-phenyl)-amide (19)

This compound was obtained as a pale yellowish white solid in 82% yield; M.p: 112–113 °C; IR (KBr) ν_max_ C = S 1350 cm^−1^; ^1^H NMR (500 MHz, CDCl_3_): δ 3.28 (s, 4H, N(CH_2_C*H*_2_)_2_NAr), 4.21 (s, 4H, N(C*H*_2_CH_2_)_2_NAr), 6.87–6.88 (d, *J* = 5.0 Hz, 1H, Ar–*H*), 7.42–7.44 (d, *J* = 10.0 Hz, 1H, Ar–*H*), 7.51–7.56 (m, 4H, Ar–*H*), 7.93 (s, 1H, Ar–*H*), 7.98–7.99 (d, *J* = 5.0 Hz, 1H, Ar–*H*), 8.67–8.68 (d, *J* = 5.0 Hz, 1H, Ar–*H*), 9.51 (br s, 1H, N*H*); ^13^C NMR (125 MHz, CDCl_3_): δ 49.47 (2C), 51.48 (2C), 109.30, 121.68, 122.04, 124.66, 126.43, 126.46, 126.58, 126.77, 126.84, 127.11, 129.01, 135.35, 142.97, 150.09, 151.90, 155.94, 183.62; ES-MS *m/z* 451 [M + H]^+^; Anal.Calcd for C_21_H_18_ClF_3_N_4_S: C, 55.94; H, 4.02; N, 12.43; found: C, 55.91; H, 4.06; N, 12.39.

### 4 -(7-Chloro-quinolin-4-yl)-piperazine-1-carbothioic acid (4-chloro-phenyl)-amide (20)

This compound was obtained as a pale yellowish white solid in 76% yield; M.p: 145–146 °C; IR (KBr) ν_max_ C = S 1358 cm^−1^; ^1^H NMR (500 MHz, CDCl_3_): δ 3.28 (s, 4H, N(CH_2_C*H*_2_)_2_NAr), 4.23 (s, 4H, N(C*H*_2_CH_2_)_2_NAr), 6.89–6.90 (d, *J* = 5.0 Hz, 1H, Ar–*H*), 7.22–7.30 (m, 4H, Ar–*H*), 7.43–7.45 (d, *J* = 10.0 Hz, 1H, Ar–*H*), 7.92–7.93 (d, *J* = 5.0 Hz, 1H, Ar–*H*), 8.00–8.02 (d, *J* = 10.0 Hz, 1H, Ar–*H*), 8.67–8.68 (d, *J* = 5.0 Hz, 1H, Ar–*H*), 9.37 (br s, 1H, N*H*); ^13^C NMR (125 MHz, CDCl_3_): δ 48.56 (2C), 51.51 (2C), 109.35, 121.71, 122.13, 126.52, 126.46, 126.61, 126.77, 126.86, 127.11, 129.01, 135.35, 142.97, 150.11, 151.93, 155.98, 183.47; ES-MS *m/z* 417 [M + H]^+^; Anal.Calcd for C_20_H_18_Cl_2_N_4_S: C, 57.56; H, 4.35; N, 13.42; found: C, 57.61; H, 4.30; N, 13.46.

### 4 -(7-Chloro-quinolin-4-yl)-piperazine-1-carbothioic acid (2,4,6-trichloro-phenyl)-amide (21)

This compound was obtained as a pale yellowish white solid in 74% yield; M.p: 132–133 °C; IR (KBr) ν_max_ C = S 1348 cm^−1^; ^1^H NMR (500 MHz, CDCl_3_): δ 3.27 (s, 4H, N(CH_2_C*H*_2_)_2_NAr), 4.27 (s, 4H, N(C*H*_2_CH_2_)_2_NAr), 6.87–6.88 (d, *J* = 5.0 Hz, 1H, Ar–*H*), 7.38 (s, 2H, Ar–*H*), 7.42–7.44 (d, *J* = 10.0 Hz, 1H, Ar–*H*), 7.93 (s, 1H, Ar–*H*), 7.97–7.99 (d, *J* = 10.0 Hz, 1H, Ar–*H*), 8.67–8.68 (d, *J* = 5.0 Hz, 1H, Ar–*H*), 9.21 (br s, 1H, N*H*); ^13^C NMR (125 MHz, CDCl_3_): δ 48.53 (2C), 51.82 (2C), 109.43, 121.79, 125.29, 126.39, 128.25, 128.74 (2C), 133.01 (2C), 134.79, 135.34, 136.16, 150.09, 152.01, 156.22, 182.46; ES-MS *m/z* 486 [M + H]^+^; Anal.Calcd for C_20_H_16_Cl_4_N_4_S: C, 49.40; H, 3.32; N, 11.52; found: C, 49.34; H, 3.29; N, 11.56.

### 4 -(7-Chloro-quinolin-4-yl)-piperazine-1-carbothioic acid naphthalen-1-ylamide (22)

This compound was obtained as a pale yellowish white solid in 55% yield; M.p: 107–108 °C; IR (KBr) ν_max_ C = S 1360 cm^−1^; ^1^H NMR (500 MHz, CDCl_3_): δ 3.27 (s, 4H, N(CH_2_C*H*_2_)_2_NAr), 4.11 (s, 4H, N(C*H*_2_CH_2_)_2_NAr), 6.81–6.82 (d, *J* = 5.0 Hz, 1H, Ar–*H*), 7.29 (br s, 1H, N*H*), 7.34–7.36 (d, *J* = 10.0 Hz, 1H, Ar–*H*), 7.42–7.44 (d, *J* = 10.0 Hz, 1H, Ar–*H*), 7.47–7.49 (d, *J* = 10.0 Hz, 1H, Ar–*H*), 7.50–7.61 (m, 2H, Ar–*H*), 7.77–7.78 (d, *J* = 5.0 Hz, 1H, Ar–*H*), 7.88–7.90 (d, *J* = 10.0 Hz, 1H, Ar–*H*), 8.00–8.09 (m, 2H, Ar–*H*), 8.12 (s, 1H, Ar–*H*), 8.73–8.74 (d, *J* = 5.0 Hz, 1H, Ar–*H*); ^13^C NMR (125 MHz, CDCl_3_): δ 49.66 (2C), 51.42 (2C), 109.19, 121.36, 121.66, 121.86, 124.67, 125.60, 125.83, 126.89, 126.98, 128.16, 128.64, 129.09, 134.51, 135.19, 136.12, 136.12, 150.15, 151.93, 155.97, 185.78; ES-MS *m/z* 433 [M + H]^+^; Anal.Calcd for C_24_H_21_ClN_4_S: C, 66.58; H, 4.89; N, 12.94; found: C, 66.61; H, 4.84; N, 12.97.

### 4 -(7-Chloro-quinolin-4-yl)-piperazine-1-carbothioic acid (2-morpholin-4-yl-ethyl)-amide (23)

This compound was obtained as a pale yellowish white solid in 70% yield; M.p: 114–115 °C; IR (KBr) ν_max_ C = S 1362 cm^−1^; ^1^H NMR (500 MHz, CDCl_3_): δ 2.53 (s, 4H, N(C*H*_2_CH_2_)_2_O), 2.65–2.68 (t, 2H, C*H*_2_), 3.31 (s, 4H, N(CH_2_C*H*_2_)_2_NAr), 3.75 (s, 4H, N(CH_2_C*H*_2_)_2_O), 3.77–3.87 (m, 2H, C*H*_2_), 4.22 (s, 4H, N(C*H*_2_CH_2_)_2_NAr), 6.63 (br s, 1H, N*H*), 6.86–6.87 (d, *J* = 5.0 Hz, 1H, Ar–*H*), 7.47–7.48 (d, *J* = 5.0 Hz, 1H, Ar–*H*), 7.97–7.99 (d, *J* = 10.0 Hz, 1H, Ar–*H*), 8.08 (s, 1H, Ar–*H*), 8.79–8.80 (d, *J* = 5.0 Hz, 1H, Ar–*H*); ^13^C NMR (125 MHz, CDCl_3_): δ 43.47, 49.38 (2C), 51.45 (2C), 51.87, 52.78 (2C), 65.67 (2C), 109.30, 121.78, 127.11, 129.12, 135.57, 142.97, 150.09, 151.90, 155.94, 183.71; ES-MS *m/z* 420 [M + H]^+^;Anal.Calcd for C_20_H_26_ClN_5_OS: C, 57.20; H, 6.24; N, 16.68; found: C, 57.12; H, 6.21; N, 16.70.

### 4 -(7-Trifluoromethyl-quinolin-4-yl)-piperazine-1-carbothioic acid phenylamide (24)

This compound was obtained as a pale yellowish white solid in 96% yield; M.p: 187–188 °C; IR (KBr) ν_max_ C = S 1367 cm^−1^; ^1^H NMR (500 MHz, CDCl_3_): δ 3.37 (s, 4H, N(CH_2_C*H*_2_)_2_NAr), 4.12 (s, 4H, N(C*H*_2_CH_2_)_2_NAr), 6.95–6.96 (d, *J* = 5.0 Hz, 1H, Ar–*H*), 7.12 (br s, 1H, N*H*), 7.13v7.25 (m, 2H, Ar–*H*), 7.31–7.37 (m, 2H, Ar–*H*), 7.65–7.69 (m, 2H, Ar–*H*), 8.11–8.13 (d, *J* = 10.0 Hz, 1H, Ar–*H*), 8.42 (s, 1H, Ar–*H*), 8.83–8.84 (d, *J* = 5.0 Hz, 1H, Ar–*H*); ^13^C NMR (125 MHz, CDCl_3_): δ 44.19 (2C), 52.18(2C), 109.63, 118.54, 120.58, 121.89, 122.27, 122.45, 124.35, 125.66, 126.27, 128.42, 128.66, 134.56, 140.41, 150.12, 151.84, 156.65, 183.21; ES-MS *m/z* 417 [M + H]^+^; Anal.Calcd for C_21_H_19_F_3_N_4_S: C, 60.56; H, 4.60; N, 13.45; found: C, 60.60; H, 4.55; N, 13.48.

### 4 -(7-Trifluoromethyl-quinolin-4-yl)-piperazine-1-carbothioic acid (2,6-dimethyl-phenyl)-amide (25)

This compound was obtained as a pale yellowish white solid in 85% yield; M.p: 105–106 °C; IR (KBr) ν_max_ C = S 1350 cm^−1^; ^1^H NMR (500 MHz, CDCl_3_): δ 2.31 (m, 6H, C*H*_3_), 3.43 (s, 4H, N(CH_2_C*H*_2_)_2_NAr), 4.32 (s, 4H, N(C*H*_2_CH_2_)_2_NAr), 6.98–6.99 (d, *J* = 5.0 Hz, 1H, Ar–*H*), 7.02 (br s, 1H, N*H*), 7.13–7.18 (m, 3H, Ar–*H*), 7.70–7.72 (d, *J* = 10.0 Hz, 1H, Ar–*H*), 8.15–8.16 (d, *J* = 5.0 Hz, 1H, Ar–*H*), 8.38 (s, 1H, Ar–*H*), 8.87–8.88 (d, *J* = 5.0 Hz, 1H, Ar–*H*); ^13^C NMR (125 MHz, CDCl_3_): δ 18.55 (2C), 48.46 (2C), 51.72 (2C), 109.26, 121.75, 123.87, 124.79, 126.68, 127.78, 128.61 (4C), 129.17, 135.82, 137.05, 150.16, 151.92, 156.16, 183.69; ES-MS *m/z* 445 [M + H]^+^; Anal.Calcd for C_23_H_23_F_3_N_4_S: C, 62.15; H, 5.22; N, 12.60; found: C, 62.11; H, 5.19; N, 12.57.

### 4 -(7-Trifluoromethyl-quinolin-4-yl)-piperazine-1-carbothioic acid (4-trifluoromethyl-phenyl)-amide (26)

This compound was obtained as a pale yellowish white solid in 80% yield; M.p: 123–124 °C; IR (KBr) ν_max_ C = S 1353 cm^−1^; ^1^H NMR (500 MHz, CDCl_3_): δ 3.37 (s, 4H, N(CH_2_C*H*_2_)_2_NAr), 4.13 (s, 4H, N(C*H*_2_CH_2_)_2_NAr), 6.99–7.00 (d, *J* = 5.0 Hz, 1H, Ar–*H*), 7.29–7.31 (m, 3H, Ar–*H*), 7.46 (br s, 1H, N*H*), 7.64–7.66 (d, *J* = 10.0 Hz, 1H, Ar–*H*), 7.70–7.72 (d, *J* = 10.0 Hz, 1H, Ar–*H*), 8.14–8.15 (d, *J* = 5.0 Hz, 1H, Ar–*H*), 8.41 (s, 1H, Ar–*H*), 8.88–8.89 (d, *J* = 5.0 Hz, 1H, Ar–*H*); ^13^C NMR (125 MHz, CDCl_3_): δ 49.51 (2C), 51.38 (2C), 109.45, 121.71, 122.44, 123.78, 124.67, 126.48, 126.51, 126.59, 126.79, 126.88, 127.15, 129.21, 135.36, 142.99, 150.29, 151.90, 155.89, 183.62; ES-MS *m/z* 485 [M + H]^+^; Anal.Calcd for C_22_H_18_F_6_N_4_S: C, 54.54; H, 3.74; N, 11.56; found: C, 54.51; H, 3.70; N, 11.59.

### 4 -(7-Trifluoromethyl-quinolin-4-yl)-piperazine-1-carbothioic acid (4-chloro-phenyl)-amide (27)

This compound was obtained as a pale yellowish white solid in 74% yield; M.p: 165–166 °C; IR (KBr) ν_max_ C = S 1356 cm^−1^; ^1^H NMR (500 MHz, CDCl_3_): δ 3.35 (s, 4H, N(CH_2_C*H*_2_)_2_NAr), 4.12 (s, 4H, N(C*H*_2_CH_2_)_2_NAr), 5.32 (br s, 1H, N*H*), 6.98–6.99 (d, *J* = 5.0 Hz, 1H, Ar–*H*), 7.16–7.18 (m, 2H, Ar–*H*), 7.35–7.37 (m, 2H, Ar–*H*), 7.70–7.72 (d, *J* = 10.0 Hz, 1H, Ar–*H*), 8.13–8.15 (d, *J* = 10.0 Hz, 1H, Ar–*H*), 8.41 (s, 1H, Ar–*H*), 8.87–8.88 (d, *J* = 5.0 Hz, 1H, Ar–*H*); ^13^C NMR (500 MHz, CDCl_3_): δ 48.34 (2C), 51.71 (2C), 110.70, 120.97, 125.10, 125.29 (2C), 127.08 (2C), 127.66, 128.36 (2C), 130.10 (2C), 139.65, 148.65, 152.33, 156.03, 182.83; ES-MS *m/z* 451 [M + H]^+^; Anal.Calcd for C_21_H_18_ClF_3_N_4_S: C, 55.94; H, 4.02; N, 12.43; found: C, 55.90; H, 3.99; N, 12.39.

### 4 -(7-Trifluoromethyl-quinolin-4-yl)-piperazine-1-carbothioic acid (2,4,6-trichloro-phenyl)-amide (28)

This compound was obtained as a pale yellowish white solid in 70% yield; M.p: 112–114 °C; IR (KBr) ν_max_ C = S 1349 cm^−1^; ^1^H NMR (500 MHz, CDCl_3_): δ 3.32 (s, 4H, N(CH_2_C*H*_2_)_2_NAr), 4.27 (s, 4H, N(C*H*_2_CH_2_)_2_NAr), 5.37 (br s, 1H, N*H*), 7.01–7.02 (d, *J* = 5.0 Hz, 1H, Ar–*H*), 7.44 (s, 2H, Ar–*H*), 7.65–7.67 (d, *J* = 10.0 Hz, 1H, Ar–*H*), 8.19–8.21 (d, *J* = 10.0 Hz, 1H, Ar–*H*), 8.24 (s, 1H, Ar–*H*), 8.77–8.78 (d, *J* = 5.0 Hz, 1H, Ar–*H*); ^13^C NMR (125 MHz, CDCl_3_): δ 48.49 (2C), 51.79 (2C), 109.39, 121.68, 123.71, 125.23, 126.45, 128.25, 128.74 (2C), 133.01 (2C), 134.79, 135.34, 136.16, 150.12, 152.11, 156.22, 183.25; ES-MS *m/z* 521 [M + H]^+^; Anal.Calcd for C_21_H_16_Cl_3_F_3_N_4_S: C, 48.52; H, 3.10; N, 10.78; found: C, 48.48; H, 3.14; N, 10.81.

### 4 -(7-Trifluoromethyl-quinolin-4-yl)-piperazine-1-carbothioic acid naphthalen-1-ylamide (29)

This compound was obtained as a pale yellowish white solid in 70% yield; M.p: 137–138 °C; IR (KBr) ν_max_ C = S 1350 cm^−1^; ^1^H NMR (500 MHz, CDCl_3_): δ 3.22 (s, 4H, N(CH_2_C*H*_2_)_2_NAr), 4.04 (s, 4H, N(C*H*_2_CH_2_)_2_NAr), 7.33–7.34 (d, *J* = 5.0 Hz, 1H, Ar–*H*), 7.47 (br s, 1H, N*H*), 7.50–7.58 (m, 2H, Ar–*H*), 7.64–7.65 (d, *J* = 5.0 Hz, 1H, Ar–*H*), 7.77–7.88 (m, 3H, Ar–*H*), 7.98–8.06 (m, 3H, Ar–*H*), 8.41 (s, 1H, Ar–*H*), 8.82–8.83 (d, *J* = 5.0 Hz, 1H, Ar–*H*); ^13^C NMR (125 MHz, CDCl_3_): δ 49.68 (2C), 51.45(2C), 109.21, 121.35, 121.68, 121.88, 123.78, 124.69, 125.62, 125.85, 126.91, 126.98, 128.18, 128.64, 129.09, 134.51, 135.19, 136.12, 136.12, 150.17, 151.93, 155.99, 184.88; ES-MS *m/z* 467 [M + H]^+^; Anal.Calcd for C_25_H_21_F_3_N_4_S: C, 64.36; H, 4.54; N, 12.01; found: C, 64.39; H, 4.58; N, 11.98.

### 4 -(7-Trifluoromethyl-quinolin-4-yl)-piperazine-1-carbothioic acid (2-morpholin-4-yl-ethyl)-amide (30)

This compound was obtained as a pale yellowish white solid in 68% yield; M.p: 134–135 °C; IR (KBr) ν_max_ C = S 1360 cm^−1^; ^1^H NMR (500 MHz, CDCl_3_): δ 2.51 (s, 4H, N(C*H*_2_CH_2_)_2_O), 2.60–2.64 (m, 2H, C*H*_2_), 3.34 (s, 4H, N(CH_2_C*H*_2_)_2_NAr), 3.71 (s, 4H, N(CH_2_C*H*_2_)_2_O), 3.75–3.78 (m, 2H, C*H*_2_), 4.13 (s, 4H, N(C*H*_2_CH_2_)_2_NAr), 6.69 (br s, 1H, N*H*), 6.94–6.95 (d, *J* = 5.0 Hz, 1H, Ar–*H*), 7.68–7.69 (d, *J* = 5.0 Hz, 1H, Ar–*H*), 8.14–8.16 (d, *J* = 10.0 Hz, 1H, Ar–*H*), 8.37 (s, 1H, Ar–*H*), 8.82–8.83 (d, *J* = 5.0 Hz, 1H, Ar–*H*); ^13^C NMR (125 MHz, CDCl_3_): δ 43.45, 49.35 (2C), 51.47 (2C), 51.89, 52.81 (2C), 65.71 (2C), 109.30, 121.78, 124.23, 127.11, 129.12, 135.57, 142.97, 150.09, 151.90, 155.94, 183.68;; ES-MS *m/z* 454 [M + H]^+^; Anal.Calcd for C_21_H_26_F_3_N_5_OS: C, 55.61; H, 5.78; N, 15.44; found: C, 55.59; H, 5.82; N, 15.41.

### Materials and methods for *in vitro* cytotoxicity screening

#### Cell lines

The human breast cancer cell lines MDA-MB468, MDA-MB231 and MCF-7 were purchased from ATCC and maintained in RPMI 1640 medium supplemented with 10% fetal bovine serum (Hyclone, Logan UT) and 2 mM L-glutamine. MCF10A and 184B5 immortalised breast cells were purchased from ATCC and maintained in mammary epithelial basal medium supplemented with an MEGM mammary epithelial singlequot kit (Cambrex). Cells were grown at 37 °C with 5% CO_2_, 95% air under the humidified conditions. Cell line authentication was carried out by Genetica DNA Laboratories (Burlington, NC) using a short tandem repeat profiling method (March 2015; July 2015; September 2016).

#### SRB assay

Cell cytotoxic effects were determined by a SRB-based protocol^2^^,^^25^.

#### Cell staining procedure

Lysosomal and AO staining were carried out as described previously^26^. Mitochondrial staining with MitoTracker was carried out as described previously^26^. Briefly, cells were plated on a poly-d-lysine pre-coated 1.8-cm sterile glass coverslip that had been placed in a well of a 6-well clustered dish. Cells were then incubated with 50 nM MitoTracker Red (ThermoFisher) for 30 min in a cell culture incubator. Plasma membrane staining was carried out with lipid-specific CellMask (Molecular probes, Life Technologies), according to the manufacturer’s instruction. For actin staining, cells fixed with 4% formaldehyde were incubated with phalloidin conjugated with Fitc (green) for 30 min. For nuclear staining, cells were incubated with 5 µM of DRAQ5 (blue) for 5 min.

## Results and discussion

### Chemistry

Twenty-six novel hybrid compounds (**5–30**) were synthesised in the present study as outlined in Scheme 1. The intermediate compounds of 7-substituted-4-piperazin-1-yl-quinoline (**3** and **4**) were prepared by aromatic nucleophilic substitution on 7-substituted-4-chloro-quinoline (**1** and **2**) with excess of piperazine in the presence of triethylamine, and the products were obtained with a simple standard workup procedure in excellent yields. Substituted-4-piperazinylquinoline derived urea (**5–16**) or thiourea (**17–30**) analogs were obtained from a “one pot-two component protocol” in which an appropriate secondary amine, substituted isocynate or thiocynate in the presence of triethylamine in anhydrous dimethyl formamide (DMF) at room temperature. The electrophilic character of the carbonylic carbon (**5–16**) is stronger than that of the thiocarbonylic carbon (**17–30**), thus facilitating the nucleophilic attack of the secondary amines. The products were purified by recrystallization with ethanol, and yields were in the range of 60–98% for 4-piperazinylquinoline derived urea analogs (**5–16**) and 55–98% for the 4-piperazinylquinoline derived thiourea analogs (**17–30**). The higher yield was observed for 4–(7-chloro-quinolin-4-yl)-piperazine-1-carboxylic acid phenylamide (**5**) due to the higher reactivity of phenylisocyanate.

The infrared (IR) spectra for the 4-piperazinylquinoline derived urea and thiourea analogs showed the absorption stretching band values in the range of 1645–1620 cm^−1^ and 1367–1348 cm^−1^ for C = O and C = S groups, respectively. The values obtained in the ^1^H NMR chemical shifts permitted the characterization of the hydrogens showing similar values for both urea and thiourea analogs with the same substituents. However, deshield effects on H-1 and H-α NMR chemical shift values of thiourea analogs were observed (c.a. *δ* 0.2–0.7) due to the heavy atom effect of sulfur. The signals of aromatic hydrogens occurred as multiplets in the characteristic region. The ^1^H NMR chemical shift of the N–H in the urea and thiourea analogs were presented a strong deshielding when compared with the N–H values of the amines, for example, *δ* 2.31 (**3**) and *δ* 6.63 (**5**), where X = Cl. ^13^C NMR spectral analysis of the urea and thiourea analogs showed the typical absorptions for aliphatic carbons in the expected region, such as the signals for the carbonylic and thiocarbonylic carbons, in the range of *δ* 154.39–156.34 and *δ* 182.46–185.78, respectively. The compounds reported in this study have also been thoroughly characterised by elemental analysis and mass spectral data.

### Antiproliferative effects of the compounds on cancer and non-cancer cells

The newly synthesised 4-piperazinylquinoline compounds (**5–30**) were evaluated for their antiproliferative effects using three breast cancer cell lines, for which the compounds were diluted to achieve seven different concentrations ranging from 100 to 1.625 µM. Followed by 48 h incubation with the compound, cells were treated with sulforhodamine B (SRB) to measure their growth/viability (% of the untreated control) by spectrophotometer^25^. The total cellular macromolecules levels known to accurately reflect by UV reading of SRB staining. The 50% growth inhibition (GI_50_) concentration for each derivative was calculated with reference to a standard curve (control cells), which represents the concentration that results in a 50% decrease in cell growth after 48 h of incubation. The resultant data are shown in Table 1. The reference compounds CQ and cisplatin were included for the comparison.

**Table 1.** Antiproliferative activity of 4-piperazinylquinoline derivatives on breast cancer and non-cancer breast cell lines.^a^

C. No	Lab code	-X	-R	GI_50_ (μM)^a^^,^^b^					Log P^c^
				MDA-MB231	MDA-MB468	MCF7	184B5	MCF10A	
**5**	RL-1	Cl	Phenyl	5.3 ± 0.5	4.7 ± 0.5	6.6 ± 0.6	14.1 ± 0.9	11.8 ± 1.4	3.2
**6**	RL-25	Cl	2,5-Dimethyl-phenyl	50.6 ± 3.2	35.5 ± 1.3	28.9 ± 1.4	19.8 ± 1.0	18.8 ± 1.3	3.7
**7**	RL-26	Cl	4-Trifluoromethyl-phenyl	21.8 ± 1.5	19.8 ± 0.8	16.6 ± 1.2	4.7 ± 0.3	4.6 ± 0.3	4.6
**8**	RL-14	Cl	2,4,6-Trichloro-phenyl	29.1 ± 1.7	30.4 ± 2.0	23.7 ± 2.4	22.3 ± 1.7	23.7 ± 1.0	4.7
**9**	RL-2	Cl	Naphthalen-1-yl	9.1 ± 1.0	7.1 ± 044	9.4 ± 0.8	21.8 ± 1.0	21.8 ± 1.0	4.4
**10**	RL-4	Cl	Cyclohexyl	20.8 ± 1.3	10.9 ± 0.8	6.0 ± 0.5	16.3 ± 1.0	15.7 ± 1.3	4.2
**11**	RL-6	CF_3_	Phenyl	20.6 ± 1.4	57.6 ± 3.2	11.0 ± 0.6	12.2 ± 1.3	21.1 ± 1.6	3.4
**12**	RL-23	CF_3_	2,5-Dimethyl-phenyl	39.6 ± 2.1	21.7 ± 1.0	22.5 ± 1.4	7.6 ± 0.7	8.8 ± 0.9	3.9
**13**	RL-24	CF_3_	4-Trifluoromethyl-phenyl	15.3 ± 1.3	2.7 ± 0.1	2.0 ± 0.1	1.1 ± 0.1	1.0 ± 0.1	4.8
**14**	RL-20	CF_3_	2,4,6-Trichloro-phenyl	21.1 ± 1.3	22.5 ± 0.2	12.6 ± 1.2	33.2 ± 2.1	17.4 ± 0.5	4.9
**15**	RL-7	CF_3_	Naphthalen-1-yl	17.8 ± 1.0	9.6 ± 0.9	7.5 ± 0.6	27.9 ± 1.7	11.1 ± 0.9	5.7
**16**	RL-9	CF_3_	Cyclohexyl	20.8 ± 2.3	12.7 ± 0.5	4.9 ± 0.3	20.8 ± 1.4	20.9 ± 1.0	4.4
**17**	RL-5	Cl	Phenyl	4.8 ± 0.2	11.8 ± 0.6	4.9 ± 0.3	19.2 ± 1.6	13.9 ± 1.3	4.3
**18**	RL-16	Cl	2,6-Dimethyl-phenyl	9.6 ± 0.5	7.8 ± 0.8	7.3 ± 0.5	16.7 ± 1.0	52.3 ± 2.9	5.3
**19**	RL-12	Cl	4-Trifluoromethyl-phenyl	14.9 ± 1.1	7.8 ± 0.8	4.9 ± 0.4	3.7 ± 0.1	3.1 ± 0.1	5.2
**20**	RL-11	Cl	4-Chloro-phenyl	22.6 ± 1.4	33.1 ± 1.5	20.4 ± 0.9	14.4 ± 0.6	8.8 ± 0.7	5.1
**21**	RL-13	Cl	2,4,6-Trichloro-phenyl	7.7 ± 0.5	5.1 ± 0.2	4.0 ± 0.3	4.7 ± 0.3	6.8 ± 0.6	6.5
**22**	RL-3	Cl	Naphthalen-1-yl	19.8 ± 1.1	10.0 ± 0.9	30.1 ± 1.2	17.8 ± 0.6	13.3 ± 1.0	5.5
**23**	RL-15	Cl	2-Morpholin-4-yl-ethyl	3.0 ± 0.1	4.6 ± 0.6	4.5 ± 0.1	34.4 ± 0.6	32.3 ± 0.8	2.8
**24**	RL-10	CF_3_	Phenyl	13.8 ± 0.8	14.1 ± 0.9	7.5 ± 0.6	24.4 ± 2.0	34.2 ± 1.3	4.6
**25**	RL-22	CF_3_	2,6-Dimethyl-phenyl	22.2 ± 1.7	13.3 ± 0.3	5.2 ± 0.5	21.39 ± 1.3	14.1 ± 1.5	5.6
**26**	RL-17	CF_3_	4-Trifluoromethyl-phenyl	16.5 ± 1.2	23.4 ± 0.9	3.8 ± 0.3	8.8 ± 0.6	10.7 ± 2.0	5.5
**27**	RL-18	CF_3_	4-Chloro-phenyl	18.7 ± 2.3	11.8 ± 1.6	7.0 ± 0.7	7.9 ± 0.6	9.2 ± 1.0	5.3
**28**	RL-19	CF_3_	2,4,6-Trichloro-phenyl	12.2 ± 0.9	23.4 ± 1.9	11.8 ± 1.0	5.5 ± 0.5	8.2 ± 0.7	6.7
**29**	RL-8	CF_3_	Naphthalen-1-yl	9.8 ± 1.0	9.4 ± 0.8	6.4 ± 0.7	50.6 ± 3.8	69.4 ± 3.7	4.6
**30**	RL-21	CF_3_	2-Morpholin-4-yl-ethyl	11.8 ± 0.9	14.2 ± 0.6	6.2 ± 0.7	17.4 ± 1.0	18.9 ± 1.3	3.1
	CQ			22.5 ± 1.4	28.6 ± 0.2	38.4 ± 0.3	76.1 ± 0.2	81.3 ± 1.4	5.1
	Cisplatin			23.6 ± 0.2	31.0 ± 0.3	25.8 ± 0.7	25.5 ± 0.6	51.5 ± 0.6	1.7

a Sigmoidal dose response curves (variable slope) were generated GraphPad Prism V. 4.02 (GraphPad Software Inc.)

b Values are the mean of triplicates of at least two independent experiments.

c Log *p* were calculated using ChemDraw Ultra V.8.0 (CambridgeSoft Corporation).

Among the 26 novel compounds synthesised and examined, sixteen compounds showed GI_50_ in the range of 3.0–19.8 µM, 11 compounds at 20.6–50.6 µM against MDA-MB231 triple-negative breast cancer cells. In the case of MDA-MB468 cells, 18 compounds showed GI_50_ range between 4.6 and 19.8 µM, eight compounds between 21.7 and 57.6 µM. As for MCF-7 cells, 21 compounds showed GI_50_ in the range of 4.0–18.6 µM, five compounds at 20.4–30.2 µM. The differences in the GI_50_ values may be attributable to a variety of factors such as nature of the seventh substitution at the 4-piperazinylquinoline ring system, the nature of urea/thiourea substitution and the genetic and biochemical background of the cell lines.

Compounds derived from the 7-chloro-4-piperazinylquinoline ring system hybridised with urea pharmacophore having a phenyl (**5**) or naphthyl (**9**) substitution showed increased antiproliferative effects on MDA-MB231, MDA-MB468 and MCF7 cells in comparison with those derived from the 7-trifluoro substituted 4-piperazinylquinoline ring system linked with urea pharmacophore having a phenyl (**11**) or naphthyl (**15**) substitution. Compounds derived from the 7-trifluoro-4-piperazinylquinoline ring system hybridised with a 2,5-dialkyl phenyl (**12**) or a 4-trifluoromethyl phenyl (**13**) urea analog showed increases of antiproliferative activity in all of the three breast cancer cell lines examined, compared to those derived from the 7-chloro substituted on the 4-piperazinylquinoline ring system with a 2,5-dialkyl phenyl (**6**) or a 4-trifluoromethyl phenyl (**7**) urea analog. Similarly, compounds derived from the 7-trifluoro-4-piperazinylquinoline ring system hybridised with a 2,4,6-trichloro phenyl (**14**) or a cyclohexyl (**16**) substituted urea analog showed increased antiproliferative activities on all three breast cancer cell lines, compared to those derived from the 7-chloro substituted 4-piperazinylquinoline ring system with a 2,4,6-trichloro phenyl (**8**) or a cyclohexyl (**10**) urea substitution. However, all of these compounds showed lower antiproliferative activity than compound **5**. Structure-activity relationship studies indicates that the introduction of small to bulky groups of liphohphilic substitution such as dialkyl (**6**), halogenated (**7** and **8**) poly aromatic hydrocarbon (naphthyl **9**) or a cyclic alkyl (cycloalkyl **10**) moiety leads to an increase in lipophilicity and decrease in the antiproliferative activity, compared to the unsubstituted phenyl compound (**6**). This further indicates that the 7-chloro-4-piperazinylquinoline ring system is favorable for antiproliferative activity. It is also clear that the replacement of the 7-chloro group with their bioisoteric functional group of the 7-trifluoromethyl substitution leads to the decrease in the antiproliferative activity on breast cancer cells^7^. The bioisoteric replacement of the chloro atom with stronger electron-withdrawing group of trifluoromethyl resulted in less potent analogs for the anticancer activity^7^^,^^27–29^.

Compounds derived from the 7-chloro-4-piperazinylquinoline ring system hybridised with thiourea pharmacophore containing phenyl (**17**), 2,6-dimethyl-phenyl (**21**), 4-trifluoromethyl-phenyl (**19**), 2,4,6-trichloro-phenyl (**21**) or 2-morpholin-4-yl-ethyl (**23**) showed better antiproliferative activity on all three breast cancer cell lines, compared to compounds **24**, **25**, **26**, **28** and **30**, all of which are derived from the 7-trifluoro substituted 4-piperazinylquinoline ring system. Conversely, compounds derived from the 7-trifluoro substituted 4-piperazinylquinoline ring system hybridised with 4-chloro-phenyl (**27**) or naphthyl (**29**) substituted thiourea analogs showed better antiproliferative activity on all three breast cancer cell lines examined, compared to those derived from the 7-chloro-4- piperazinylquinoline ring system with 4-chloro-phenyl (**20**) or naphthyl (**22**) substituted thiourea. However, all of these compounds showed lower antiproliferative activity than compound **23** (RL-15), which contains morpholinyl ethyl substitution with Log *p* values of 2.9, as it exhibited GI_50_ values of 5.3, 4.7 and 4.5 µM against MDA-MB231, MDA-MB468 and MCF7 cells, respectively. Our results clearly demonstrated that the 7-chloro-4-piperazinylquinoline ring system is more favorable for antiproliferative activity on breast cancer cells than other substitutes, and directly correlated with our earlier studies^7^.

Among the series, thiourea compounds **19**, **21**, **24**, **28** and **29** showed stronger activities against all three different breast cancer cell lines. In this case, the more lipophilic property of thiourea may play an important role. For example, the Log *p* values of the thiourea compounds **28**, which has a higher Log *p* values (6.7), is more effective than the corresponding urea compound **10**, which has lower Log *p* values (4.2). In fact, all of the thiourea containing compounds with higher Log *p* values are more active, compared to the corresponding urea analogs. It may be due, at least in part, to the differences in electronegativitiy of sulfur and oxygen atoms on thiourea and urea analogs.

Compounds **5**, **9, 23** and **29** show 4-8-fold higher activity than the reference compounds cisplatin and CQ. Among them, compound **23** is the most effective as its GI_50_ values are 3.0, 4.6 and 4.5 µM against MDA-MB231, MDA-MB468 and MCF7 cells, respectively.

**Figure 3.**
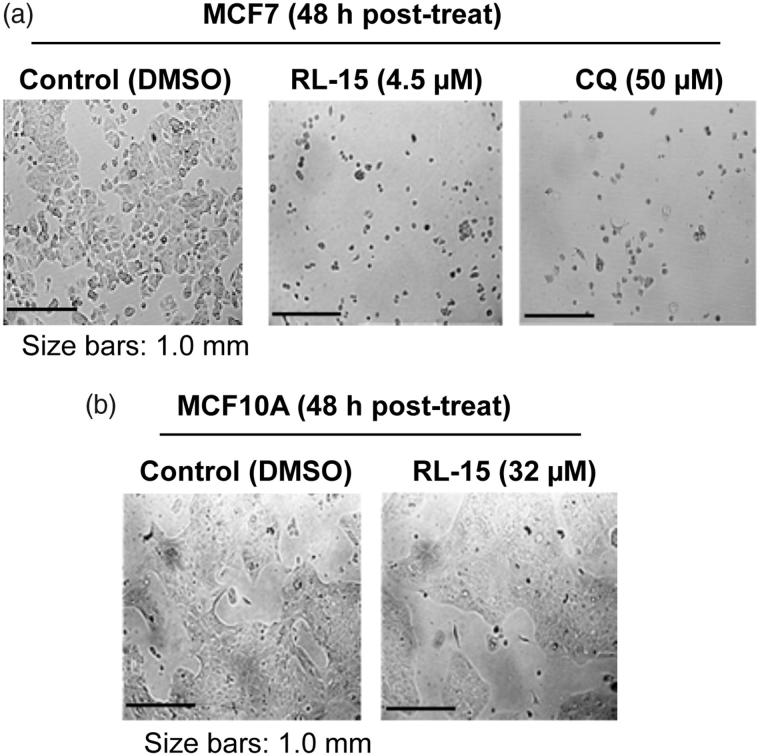
MCF7 breast cancer cells are much more sensitive to compound **23** (RL-15) than MCF10A non-cancer breast cells. CQ is a reference compound as compound **23** and CQ share the basic chemical scaffold.

We further assessed their antiproliferative effects on two non-cancer immortalised cell lines (184B5 and MCF10A). As shown in Table 1, the antigrowth effects of compounds **8**, **10**, **11**, **14**, **15**, **16**, **21**, **22**, **25**, **26** and **27** are comparable against cancer and non-cancer cells, although some of them are more active against certain cell lines (e.g. compounds **8** and **10**). Remarkably, the following compounds show higher activity against non-cancer cells than cancer cells: **6**, **7**, **12**, **13**, **19**, **20** and **28**. In contrast, compounds **5**, **9, 17**, **18**, **23**, **24**, **29** and **30** showed much stronger activities against cancer than non-cancer cells. Among these compounds, compound **23** (RL-15) is the most desirable since its GI_50_ values against non-cancer cell lines are 34.4 μM (184B5) and 32.3 µM (MCF10A) (Table 1; Figure 3). The antiproliferative effects of compound **23** on cancer cells are thus 7-fold (MDA-MB468 *vs* MCF10A) to 11-fold (MDA-MB231 *vs* 184B5) higher than matching non-cancer breast cells, which may indicate that compound **23** could potentially be safer than other compounds. Therefore, we examined it further to gain insights into its molecular mechanism of action.

### Cancer cells lose plasma membrane integrity in the presence of compound 23 (RL-15)

**Figure 4.**
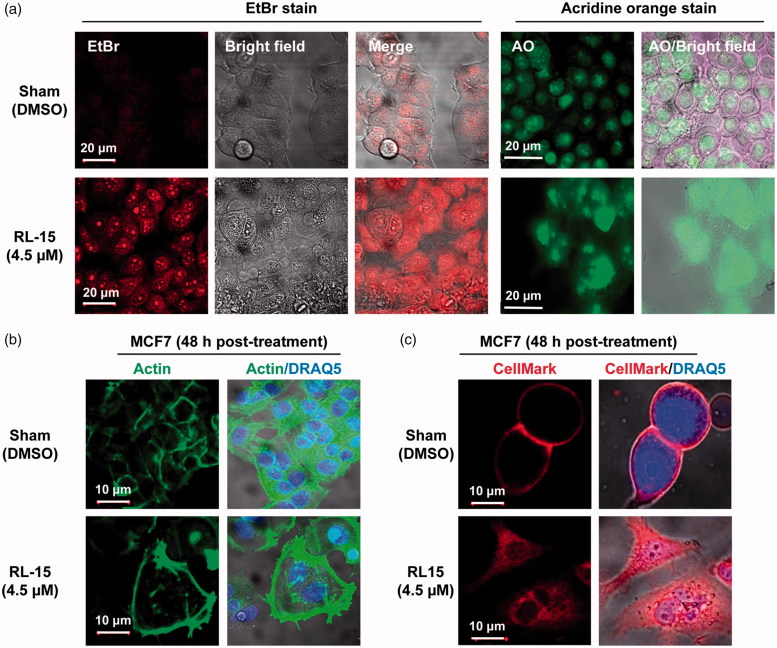
The treatment of cancer cells with compound **23** resulted in the loss of plasma membrane integrity. (a) MCF7 cell morphology changes in the presence of compound **23**. MCF7 cells were sham treated or treated with compound **23** for 48 h, followed by staining with either EtBr. Cell samples were mixed with staining solution immediately prior to microscopy. (b) The structure of actin filaments on the plasma membrane appears to be intact in the presence of compound **23**. (c) MCF7 cells were stained with the lipid-specific CellMask.

In contrast to the Sham control, MCF7 breast cancer cells treated with compound **23** readily uptake ethidium bromide (EtBr), suggesting that the plasma membrane may have been compromised (Figure 4(a), left panels). This conclusion is further strengthened by data obtained from acridine orange (AO) staining, which also showed abnormal (swollen) cell morphology (Figure 4(a), right panels). However, data from phalloidin stained MCF7 cells indicated that the structure of actin filaments at the plasma membrane may largely be intact, even though some cells are apparently enlarged (Figure 4(b)). Interestingly, the entire cytoplasm was stained by the lipid-specific CellMask in the cells treated with compound **23** (Figure 4(c)). Thus, these data are consistent with the notion that the lipid structure on the plasma membrane has been compromised in response to compound **23** (Figure 4(c)). However, it is currently unclear how lipid molecules are found throughout the entire cytoplasm. We found that 42.4 ± 4.8% of 500 cells examined showed compromised plasma membrane phenotypes by 24-h post treatment. In addition, cells treated with compound **23** are often enlarged and flattened, and contained multi-nuclei. In addition, many small vacuoles are found in the cytoplasm of these cells (Figure 4(a–c)). All of these phenomena are likely leading to eventual cancer cell demise in the presence of compound **23**.

### The mitochondria and lysosome are increased in volumes and disorganised

**Figure 5.**
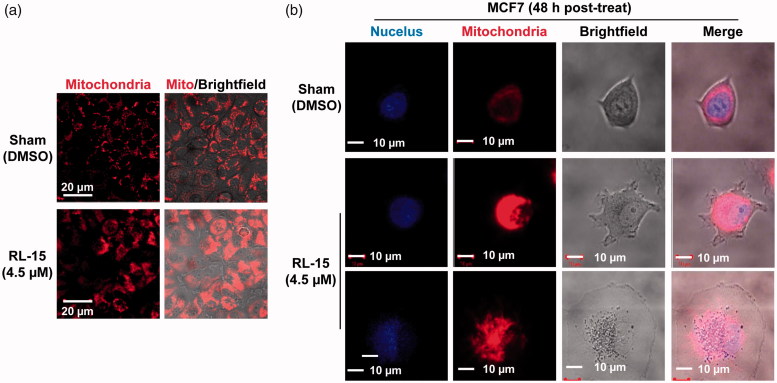
Mitochondrial membrane may be compromised in the presence of compound **23**. (a) The mitochondrial volumes are substantially increased. (b) Mitochondria are aggregated and disorganized in the presence of compound **23**. MCF7 mitochondria and nuclei were stained with MitoTracker and DRAQ5, respectively, as descried in Materials and methods.

**Figure 6.**
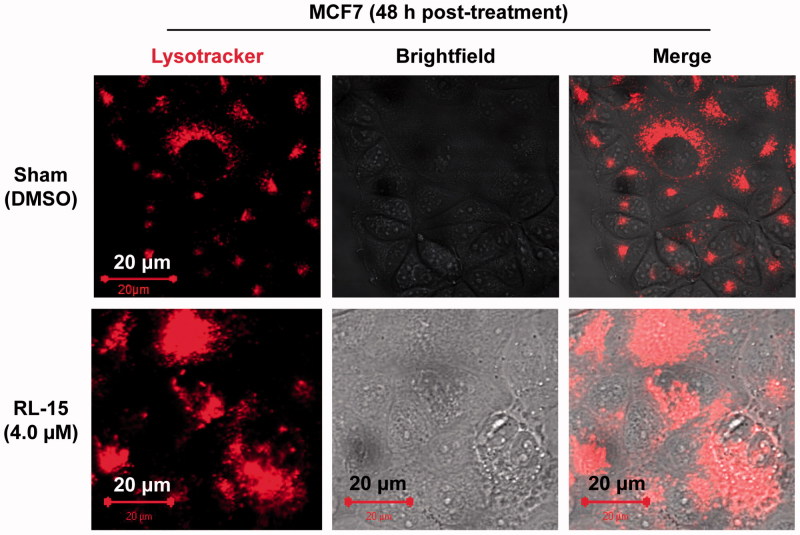
Lysosomes are increased in volume and often heavily aggregated in the presence of compound **23**. MCF7 cells were stained with LysoTracker.

The MCF7 cells treated with compound **23** showed substantial increases in mitochondrial and lysosomal volumes, often in a disorganised form (Figures 5 and 6). It should be noted that these abnormalities could occur if the membranes of these organelles are compromised. Thus, the molecular target of compound **23** is likely a common element found in the membranes of cytoplasm, mitochondria and lysosomes, but not in the nuclear membrane as it is not compromised in the presence of compound **23** (Figures 4–6). Finally, our data also show that nucleus is often pushed to one side (Figure 5(b)), probably due to the increase in volumes and disorganization of mitochondria and lysosomes.

## Conclusion

We here describe the hybrid pharmacophore design and synthesis of a new series of 4-piperazinylquinoline derivatives. Data from an *in vitro* study showed that most of the compounds derived from 4-piperazinylquinoline exhibited promising anticancer activity against human breast cancer cells. Among the 26 novel compounds, compounds **5**, **9**, **17**, **18**, **23** and **29** showed significantly improved antigrowth/antiproliferative activity against MDA-MB231, MDA-MB468 and MCF7 breast cancer cells. Comparing those hybrid compounds containing thiourea or urea, the former is generally more active than the latter as compounds **19**, **21**, **24**, **28** and **29** showed stronger activities than their corresponding urea analog compounds **7**, **8**, **11**, **14** and **15** against all three different breast cancer cell lines. Since all of these compounds required higher drug concentration to achieve the same GI_50_ value against the 184B5 and MCF10A non-cancer breast cell lines, they could be safer than other compounds. Among them, compound **23** (4-(7-chloro-quinolin-4-yl)-piperazine-1-carbothioic acid (2-morpholin-4-yl-ethyl)-amide) is considered to be the most desirable, since its antiproliferative activity is 7–11 fold higher on cancer than non-cancer cells. Data from cell morphology study demonstrated that the membrane integrity of the cytoplasm, mitochondria and lysosome is compromised in the cells treated with compound **23**, perhaps through the disruption of the lipid structure in these membranes. However, the nuclear membrane is not compromised, suggesting that the target molecule of compound **23** may be a common element found on the membranes of the cytoplasm, mitochondria and lysosome, but not in the nuclear membrane. Overall, our data suggest that hybrid compounds containing the 4-piperazinylquinoline pharmacophore and thiourea can be promising leads, and this new hybrid approach can be an excellent way of developing effective anti-breast cancer agents.

## Supplementary Material

Supplemental Material
