# Supplementary material for: Design and synthesis of 4-piperazinyl quinoline derived urea/thioureas for anti-breast cancer activity by a hybrid pharmacophore approach
Source: J Enzyme Inhib Med Chem. 2019 Feb 6;34(1):620–30. doi: 10.1080/14756366.2019.1571055 (PMC6366420; doi:10.1080/14756366.2019.1571055)
Supplement: Supplemental Material [file IENZ_A_1571055_SM9531.pdf]

Supplementary information

Design and synthesis of 4-piperazinylquinoline derived urea/thioureas for anti-breast cancer activity by hybrid pharmacophore approach

V. Raja Solomon*, Sheetal Pundir, and Hoyun Lee*

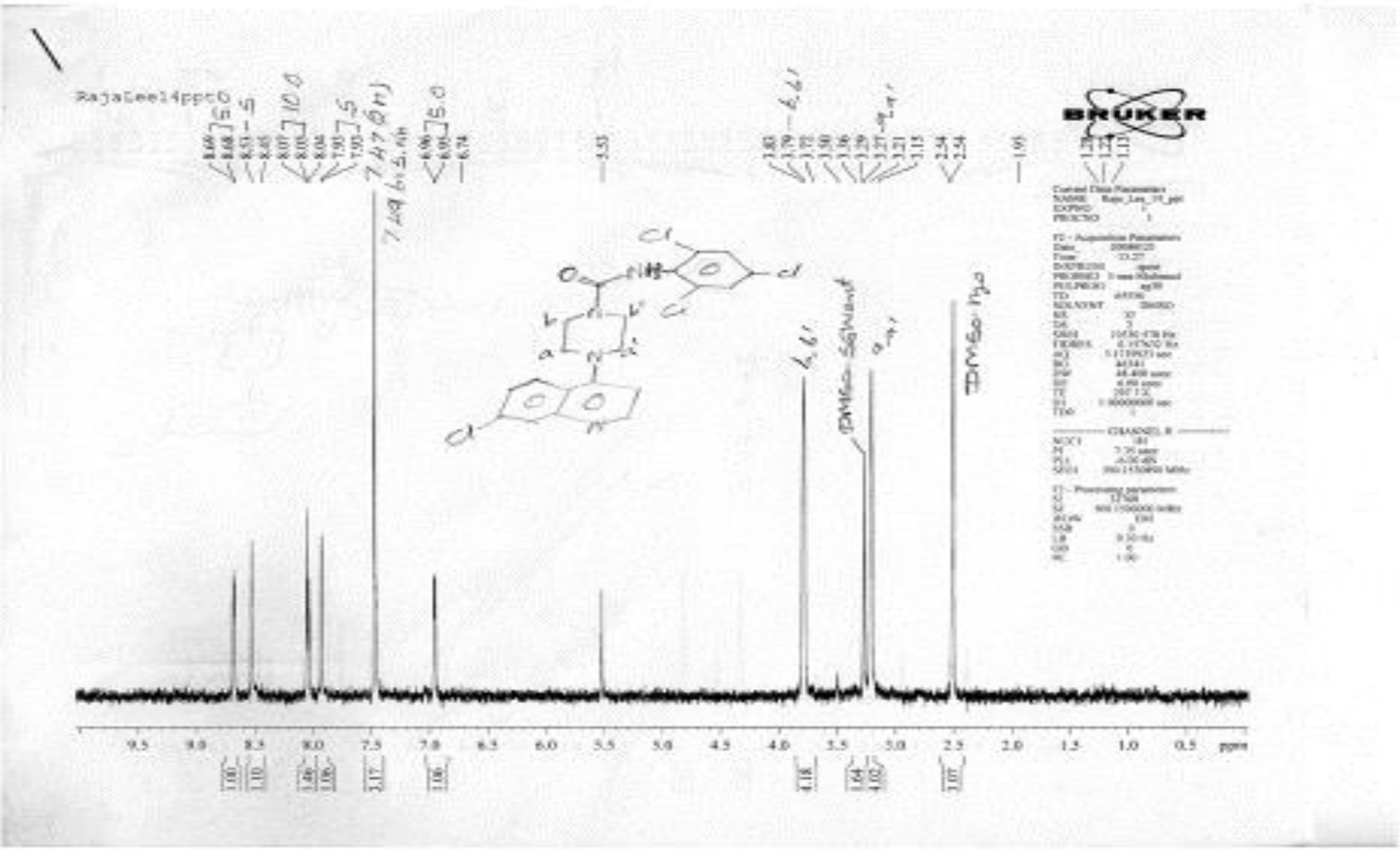

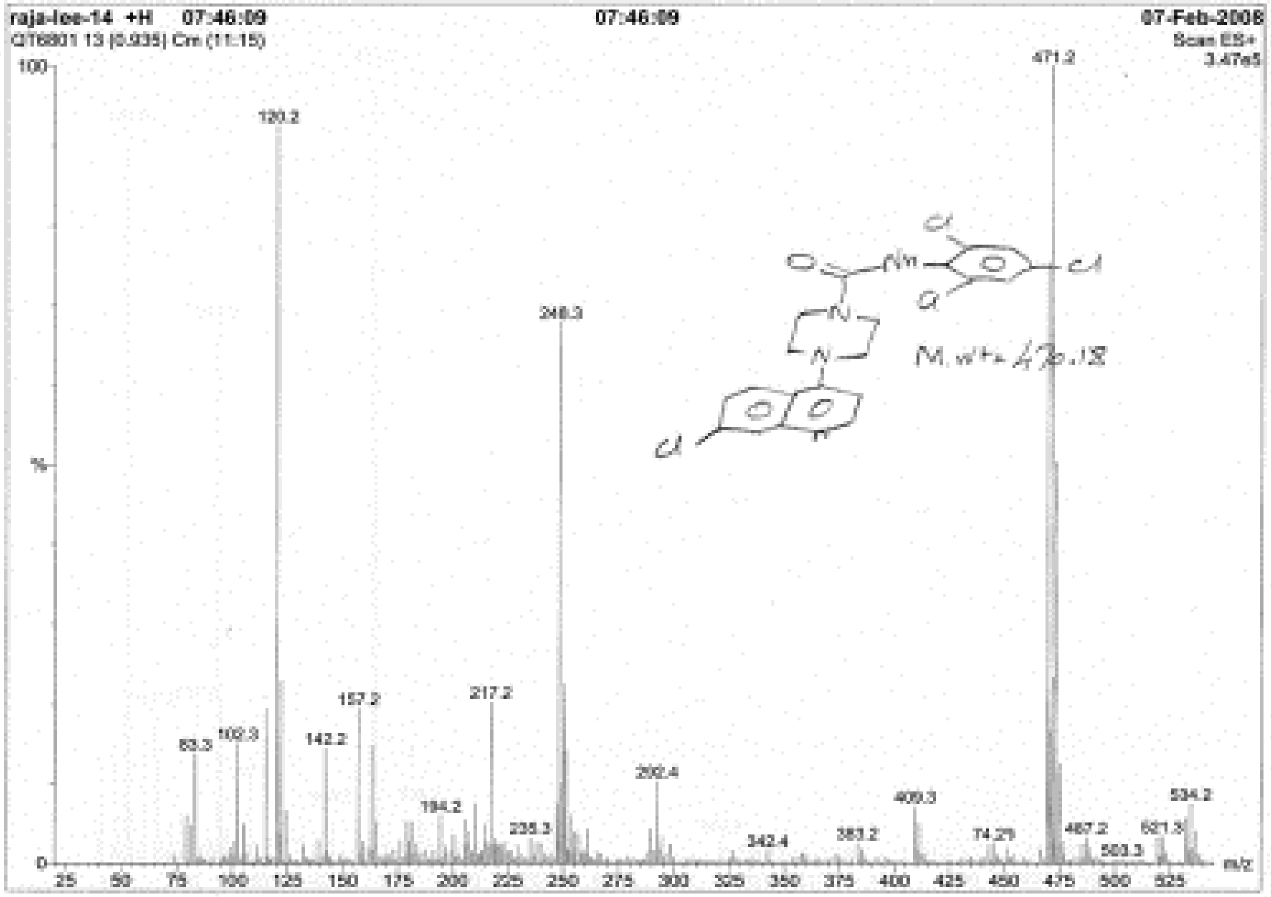
raja-lee-14 +H 07:46:09
07:46:09
07-Feb-2008
QT6801 13 (0.935) Cm (11:15)
Scan ES+
3.47e5
471.2
120.2
248.3
M. wt= 470.18
217.2
157.2
102.3
83.3
142.2
292.4
194.2
409.3
534.2
235.3
342.4
383.2
487.2
521.3
503.3
m/z

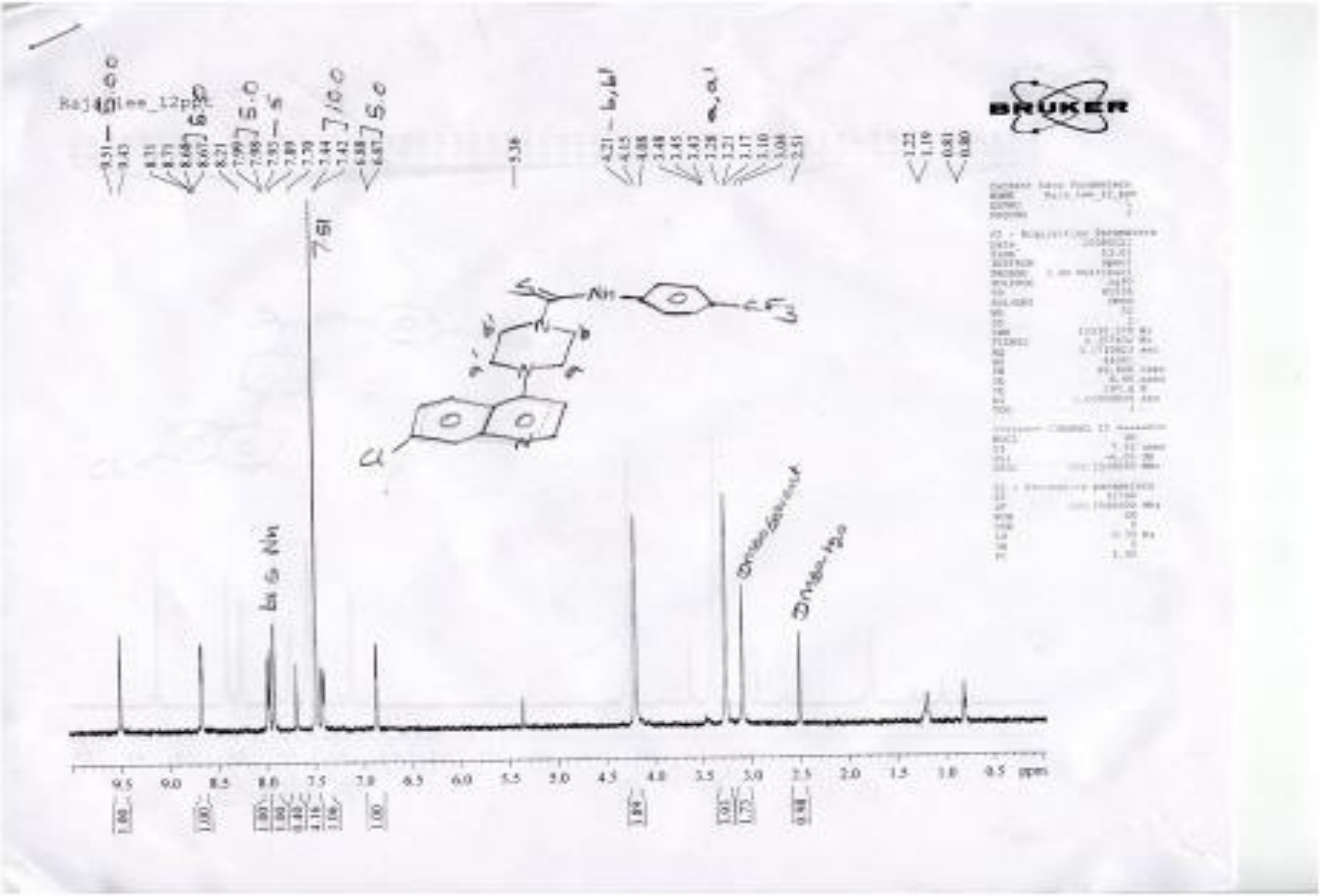

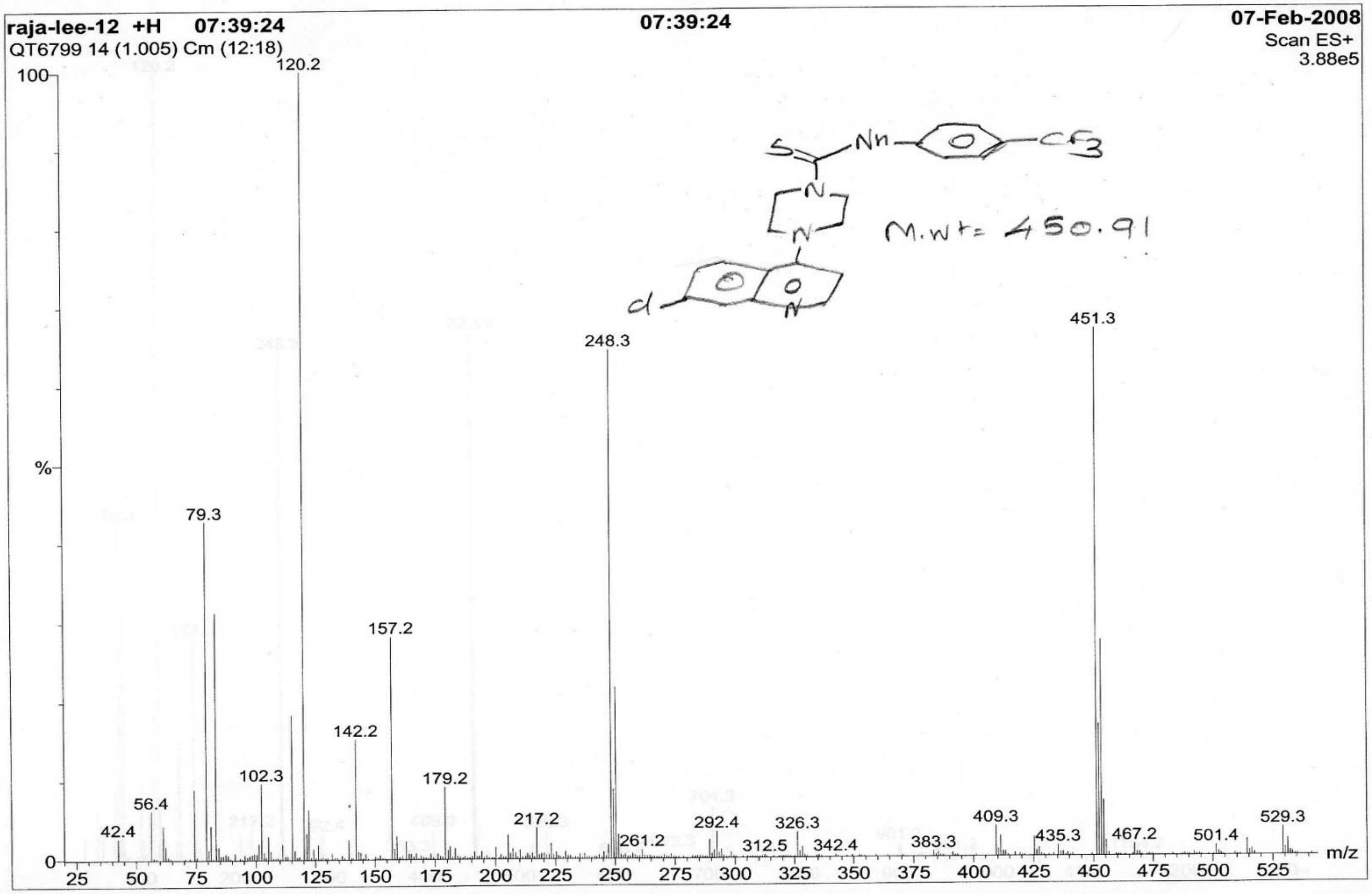
raja-lee-12 +H 07:39:24
QT6799 14 (1.005) Cm (12:18)
07:39:24
07-Feb-2008
Scan ES+
3.88e5
100
%
0
120.2
248.3
451.3
79.3
157.2
142.2
102.3
179.2
56.4
42.4
217.2
261.2
292.4
312.5
326.3
342.4
383.3
409.3
435.3
467.2
501.4
529.3
25 50 75 100 125 150 175 200 225 250 275 300 325 350 375 400 425 450 475 500 525
m/z
M.wt= 450.91

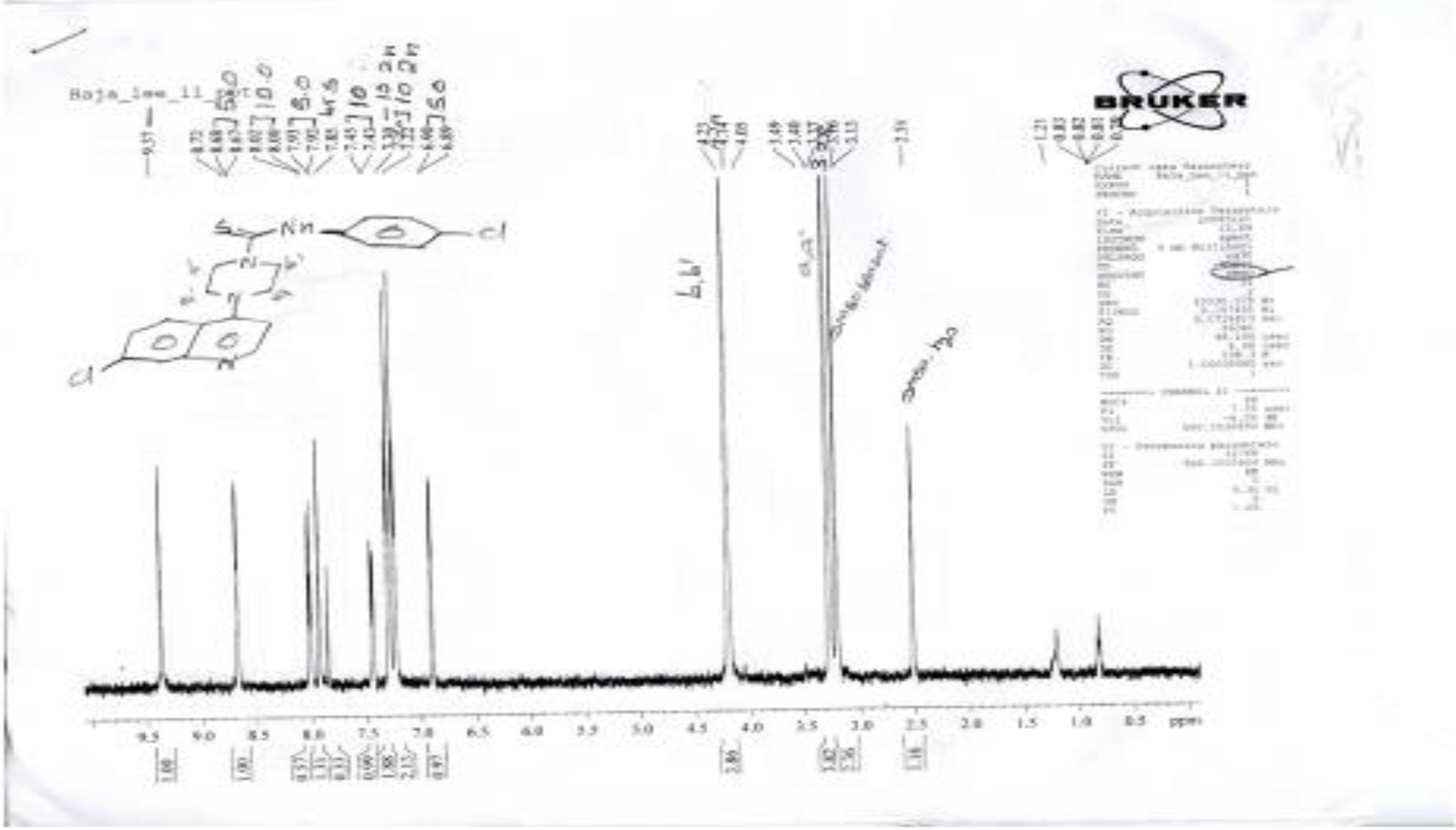
BRUKER

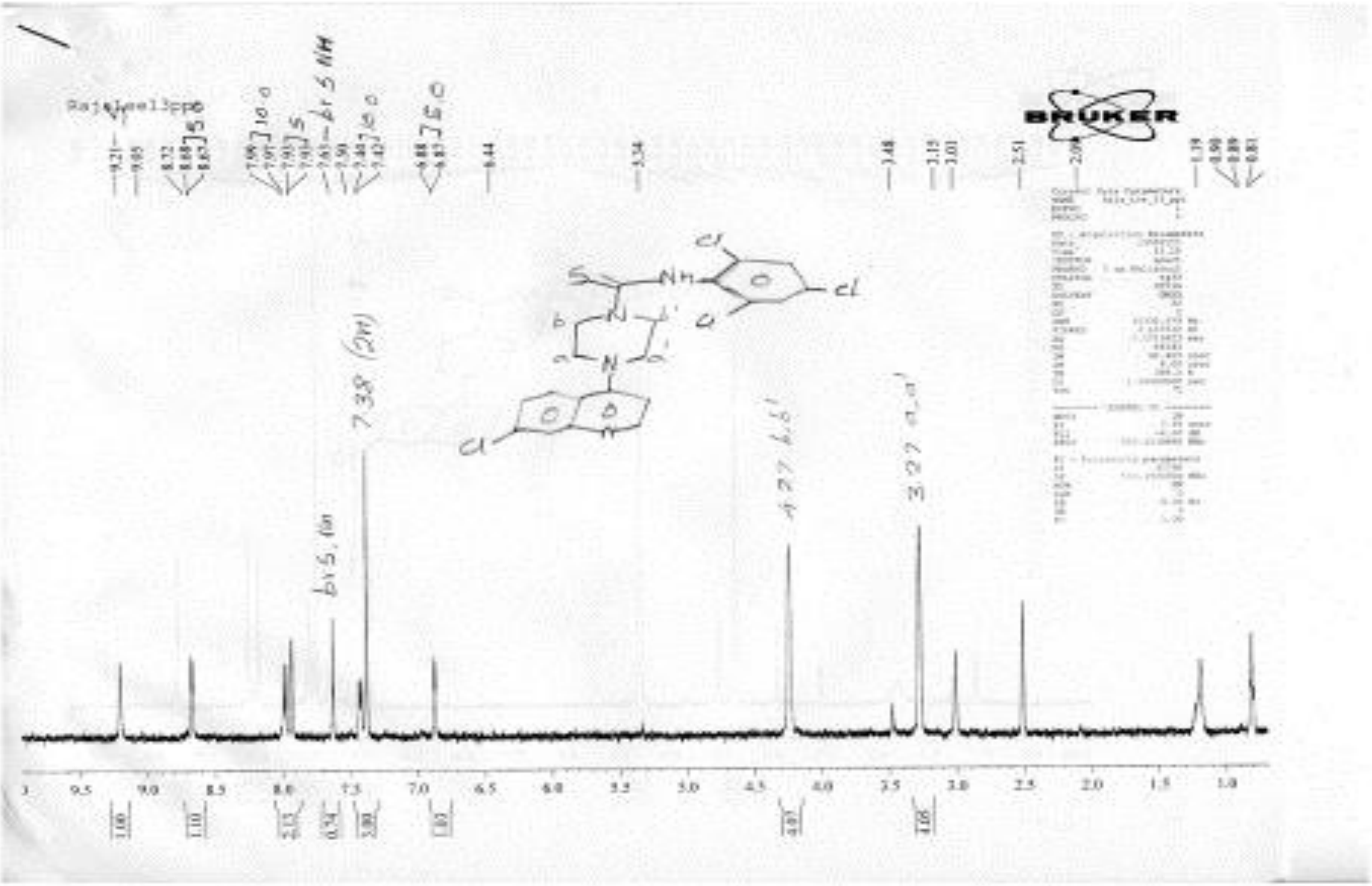

BRUKER
7.38 (2H)
4.27 b,b'
3.27 a,a'

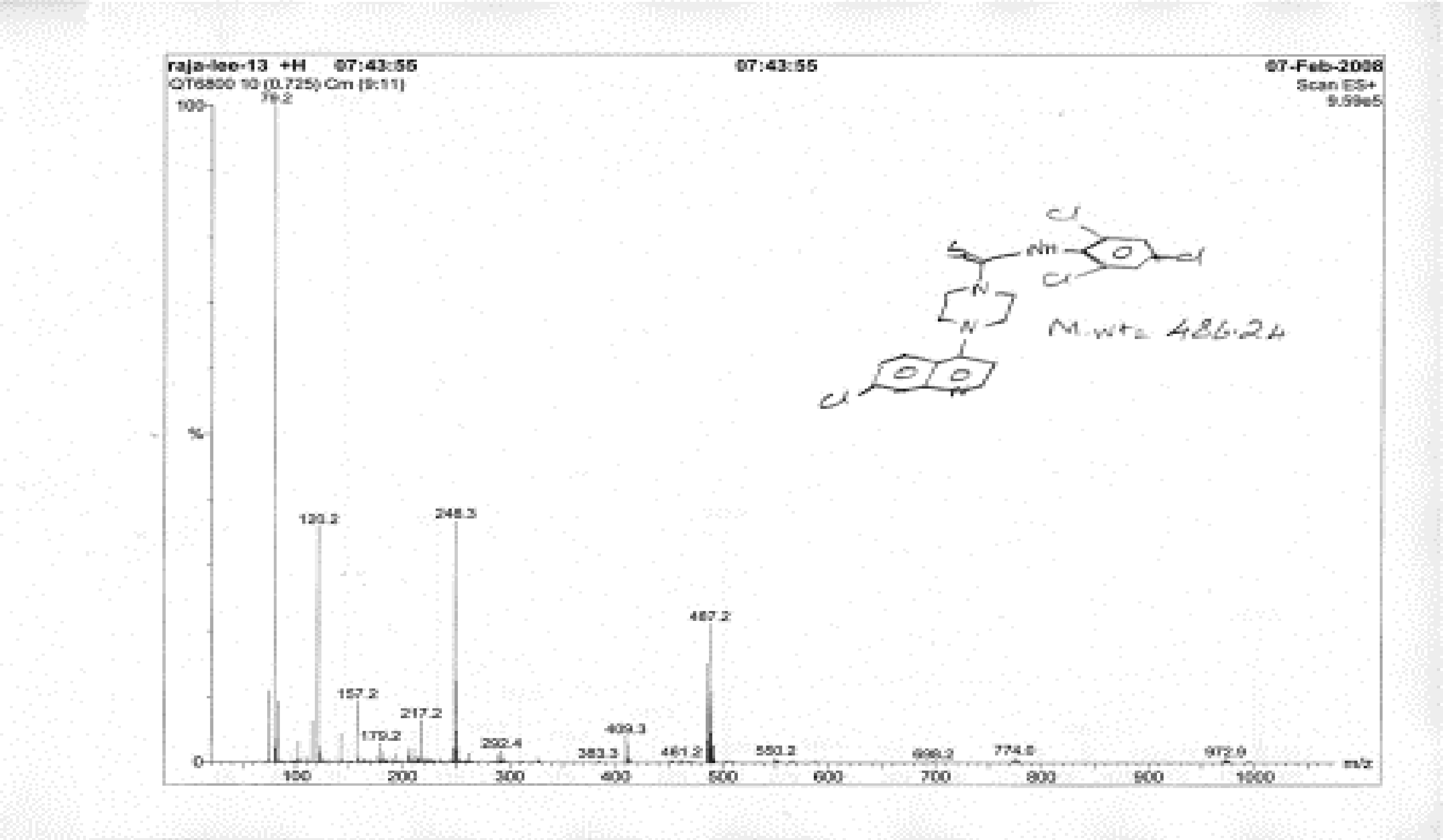
raja-lee-13 +H 07:43:55
07:43:55
07-Feb-2008
QT6800 10 (0.725) Cm (9:11)
Scan ES+
9.59e5
100
%
0
79.2
120.2
248.3
157.2
217.2
179.2
292.4
409.3
383.3
461.2
487.2
550.2
774.0
972.0
100
200
300
400
500
600
700
800
900
1000
m/z
M.wt= 486.24

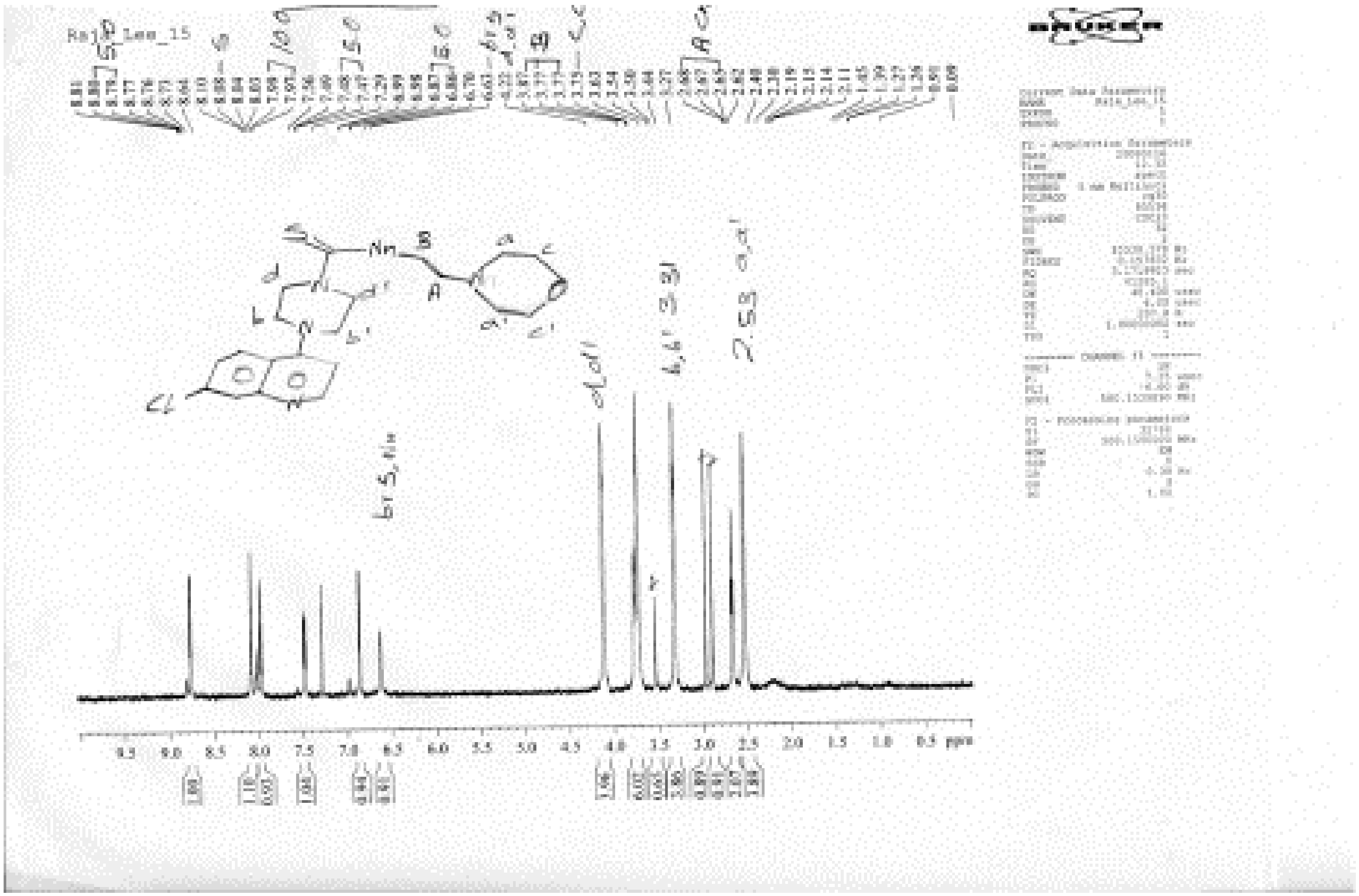

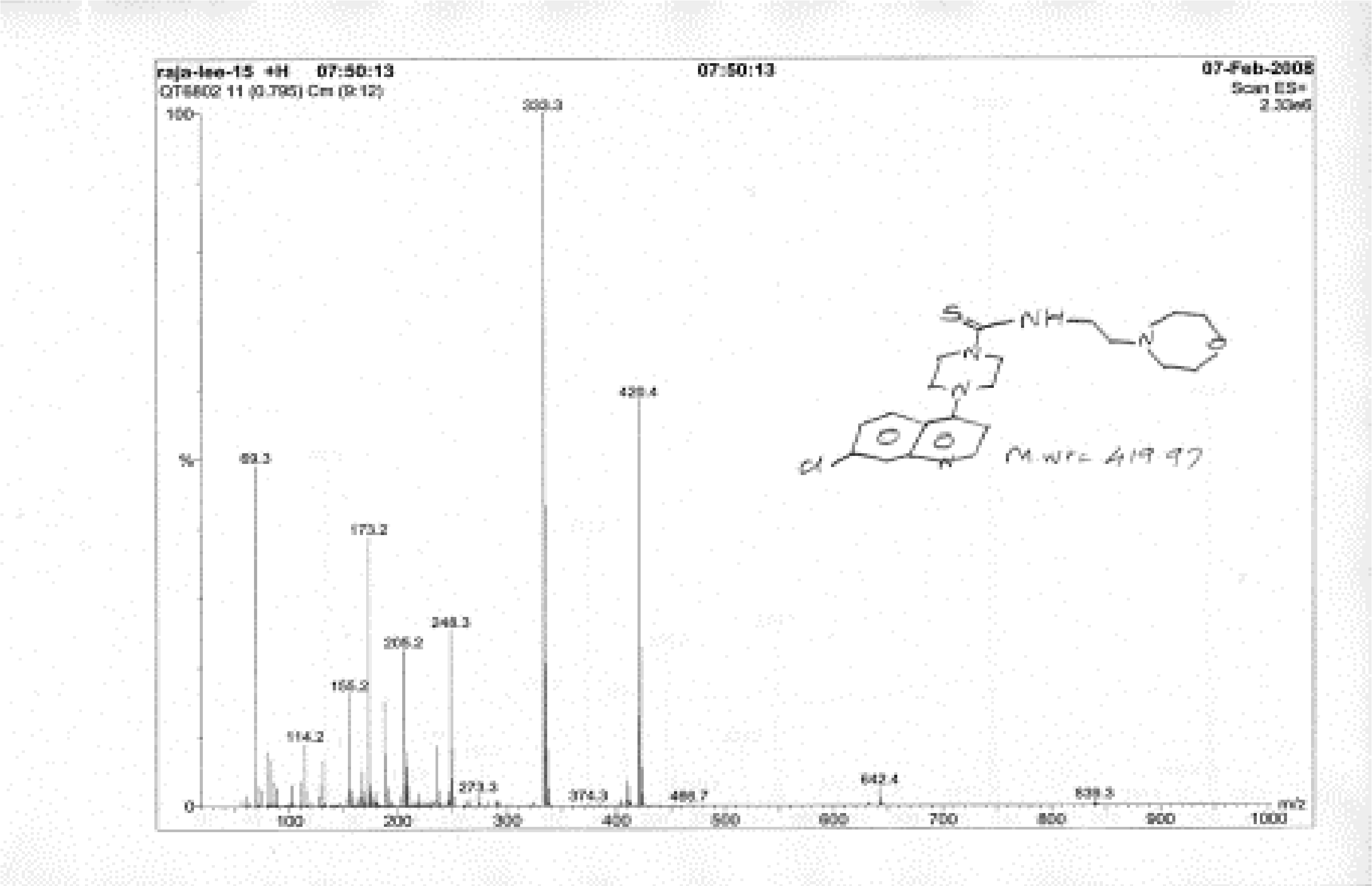
raja-lee-15 +H 07:50:13
07:50:13
07-Feb-2008
2.33e6
100
%
0
173.2
248.3
205.2
156.2
114.2
273.3
420.4
374.3
466.7
642.4
839.3
100
200
300
400
500
600
700
800
900
1000
m/z

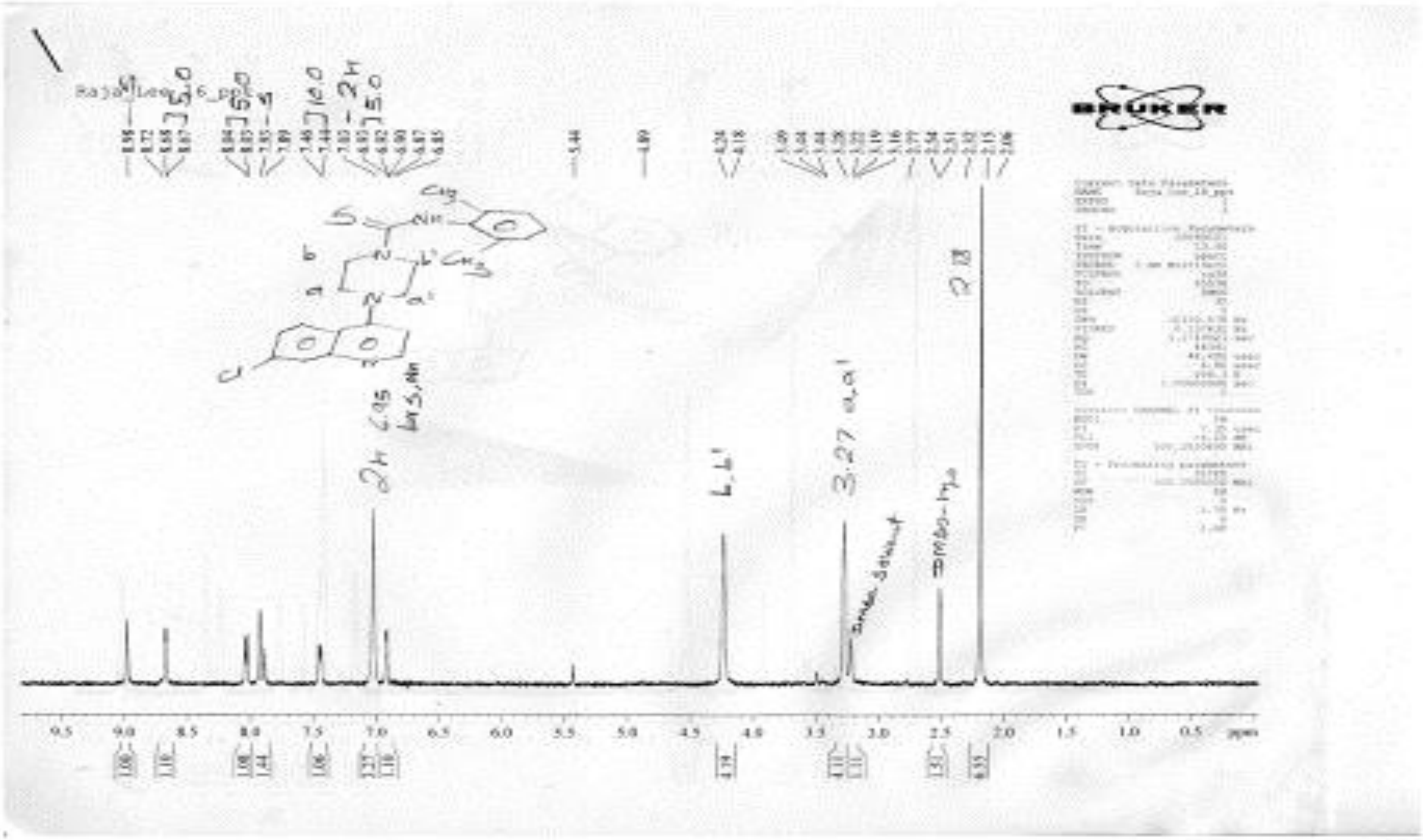

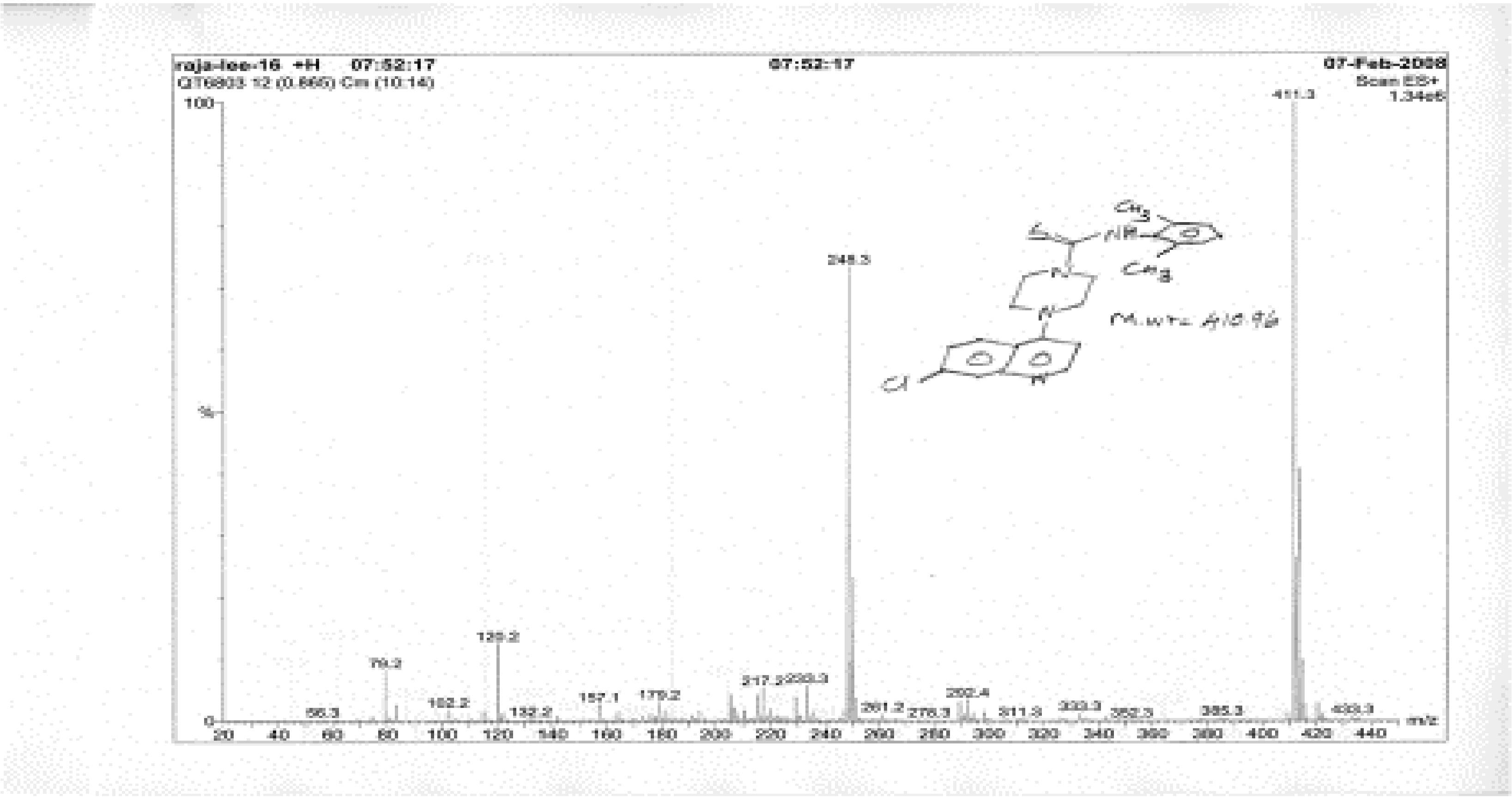

raja-lee-16 +H 07:52:17
07:52:17
07-Feb-2008
Scan ES+
1.34e6
411.3
248.3
129.2
79.2
M.wt= 410.96
m/z

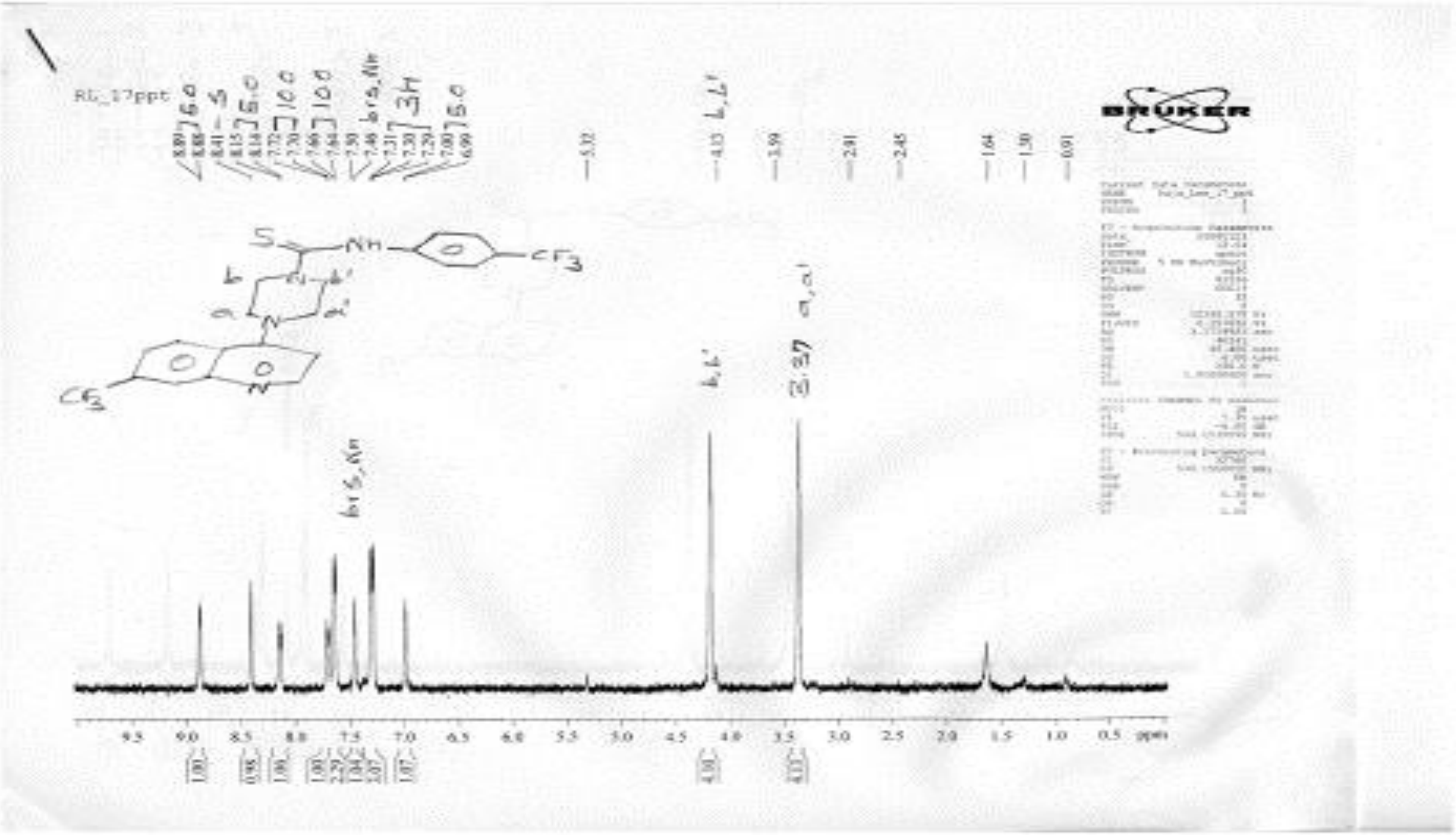
RL_17ppt
BRUKER
5.32
4.15
3.39
2.91
2.45
1.64
1.30
0.91
9.5 9.0 8.5 8.0 7.5 7.0 6.5 6.0 5.5 5.0 4.5 4.0 3.5 3.0 2.5 2.0 1.5 1.0 0.5 ppm

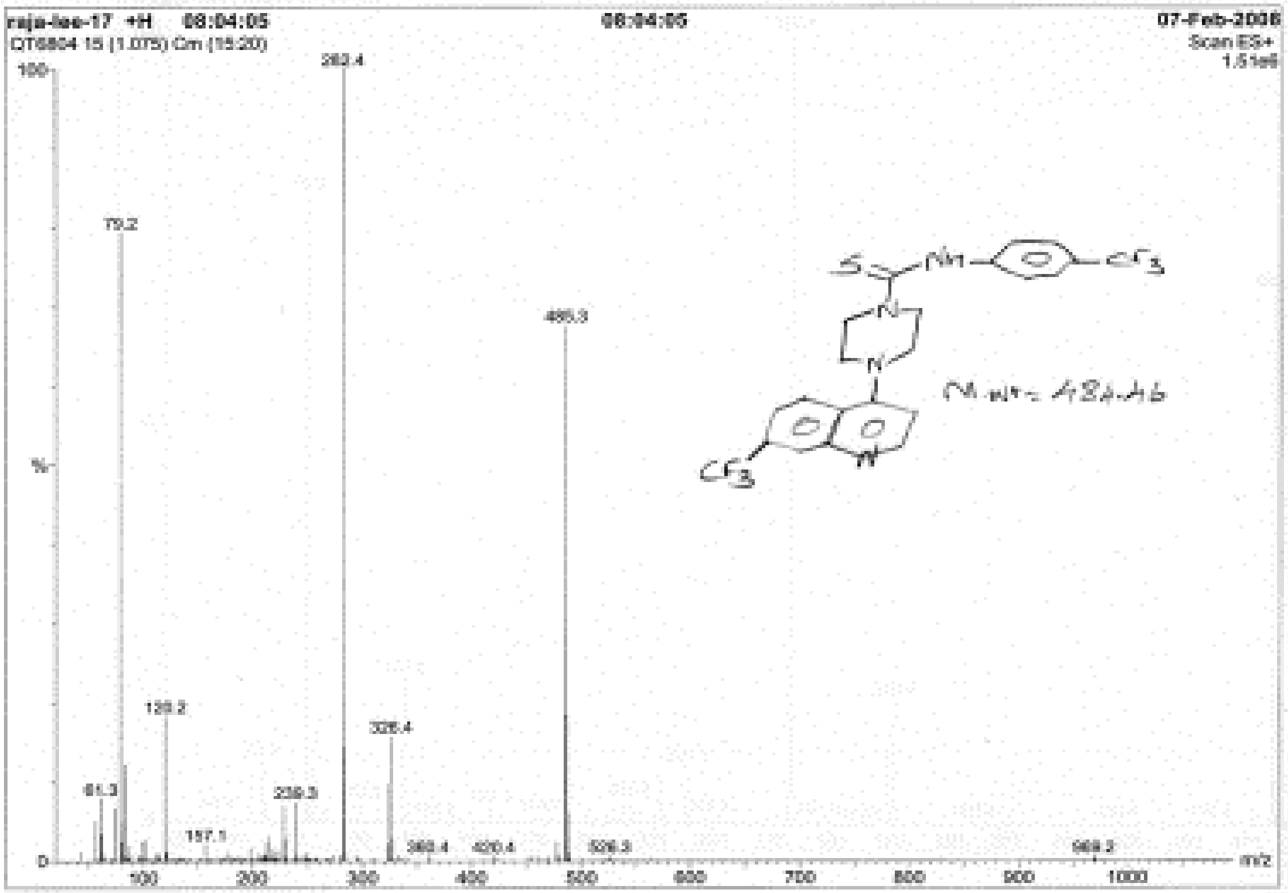
raja-lee-17 +H 08:04:05
08:04:05
07-Feb-2006
QT6804 15 (1.075) Cm (15:20)
Scan ES+
1.51e6
100
%
0
282.4
79.2
485.3
120.2
326.4
81.3
239.3
157.1
360.4
420.4
526.3
969.2
100
200
300
400
500
600
700
800
900
1000
m/z

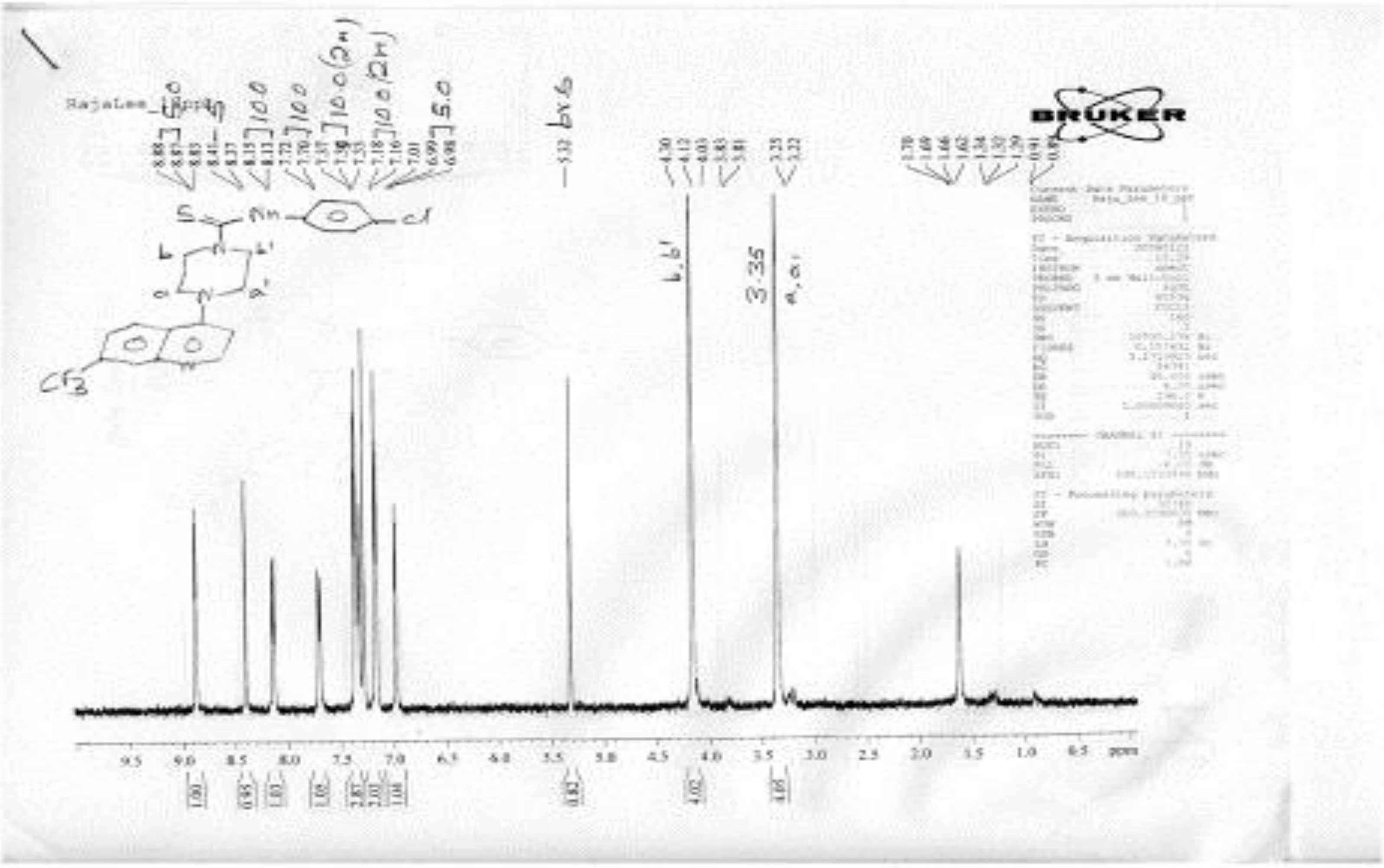

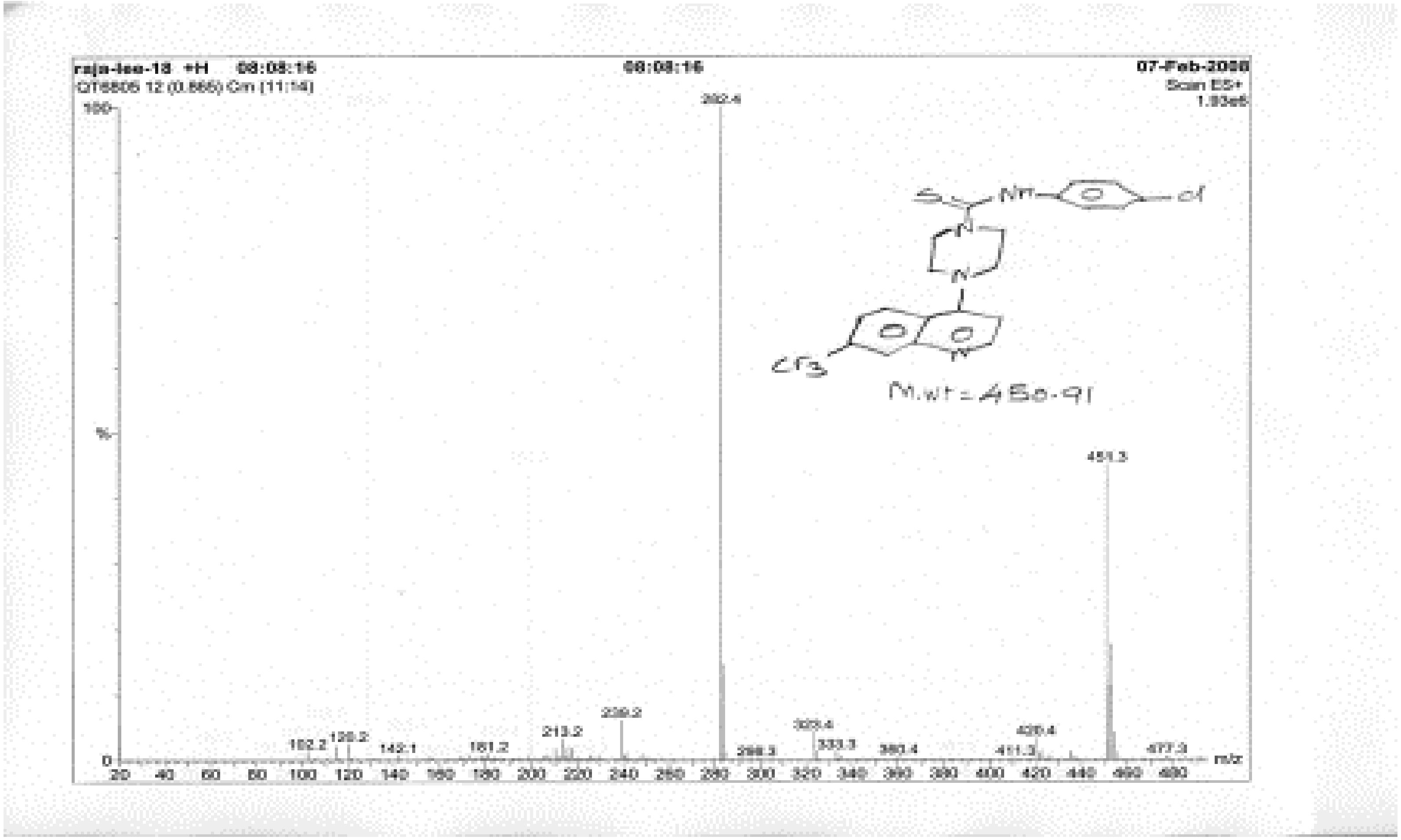
raja-lee-18 +H 08:08:16
08:08:16
07-Feb-2008
Scan ES+
1.93e6
100
%
0
282.4
451.3
239.2
213.2
323.4
420.4
102.2
120.2
142.1
181.2
298.3
333.3
363.4
411.3
477.3
m/z
M.wt = 450.91

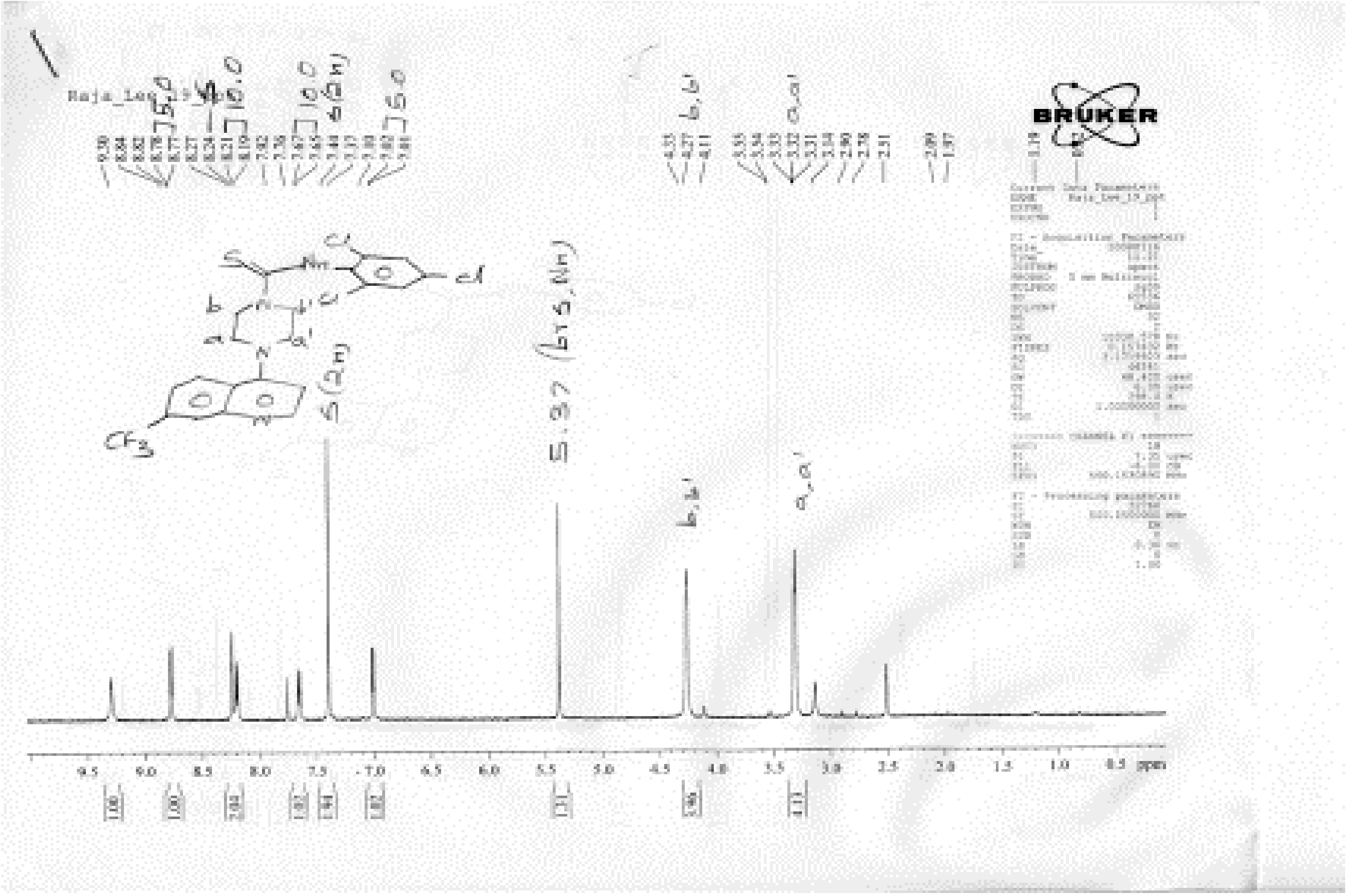

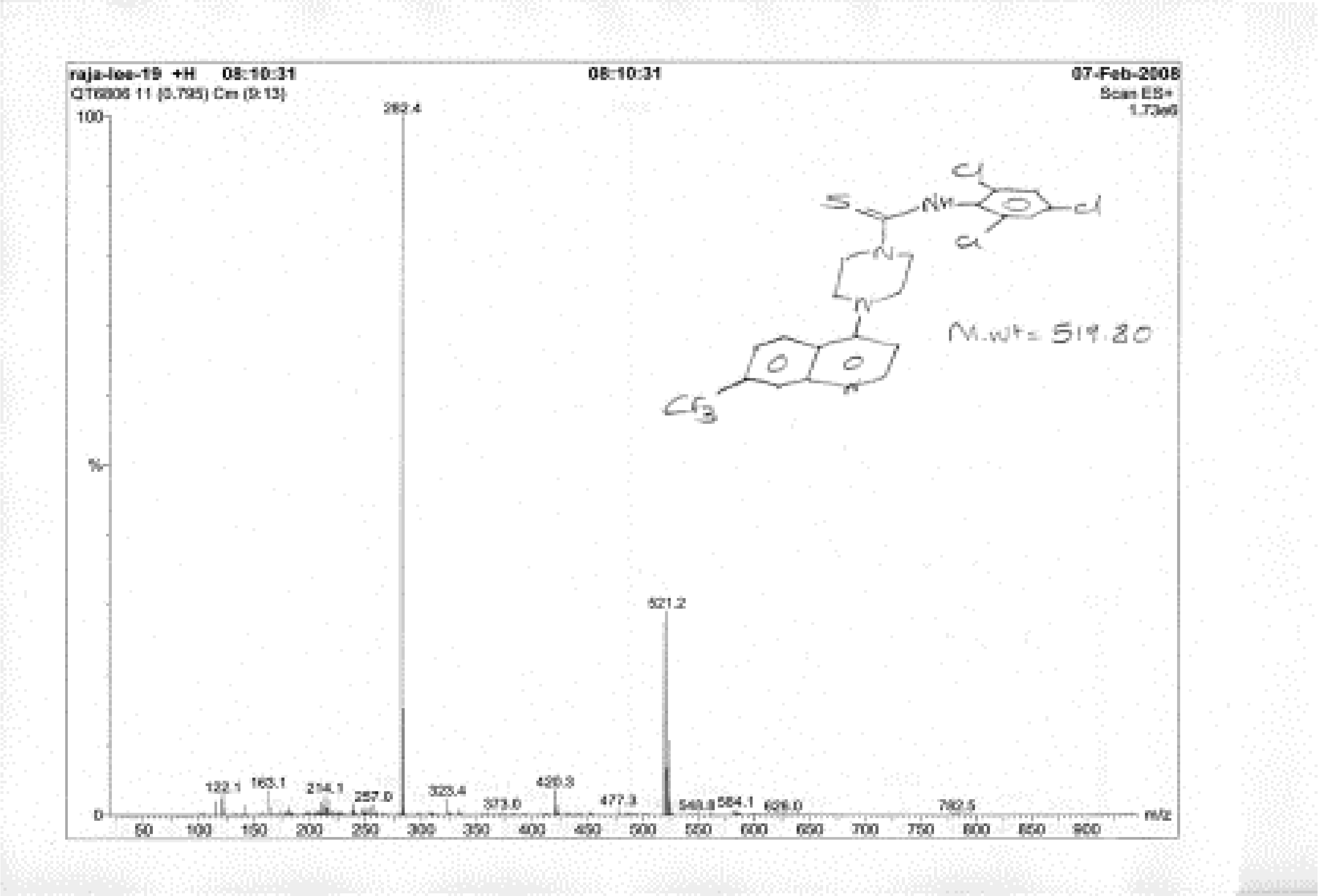

raja-lee-19 +H 08:10:31
08:10:31
07-Feb-2008
QT6806 11 (0.795) Cm (9:13)
Scan ES+
1.73e6
100
%
0
282.4
521.2
132.1
163.1
214.1
257.0
323.4
373.0
420.3
477.3
540.8
584.1
626.0
792.5
m/z
50 100 150 200 250 300 350 400 450 500 550 600 650 700 750 800 850 900
M.wt= 519.80

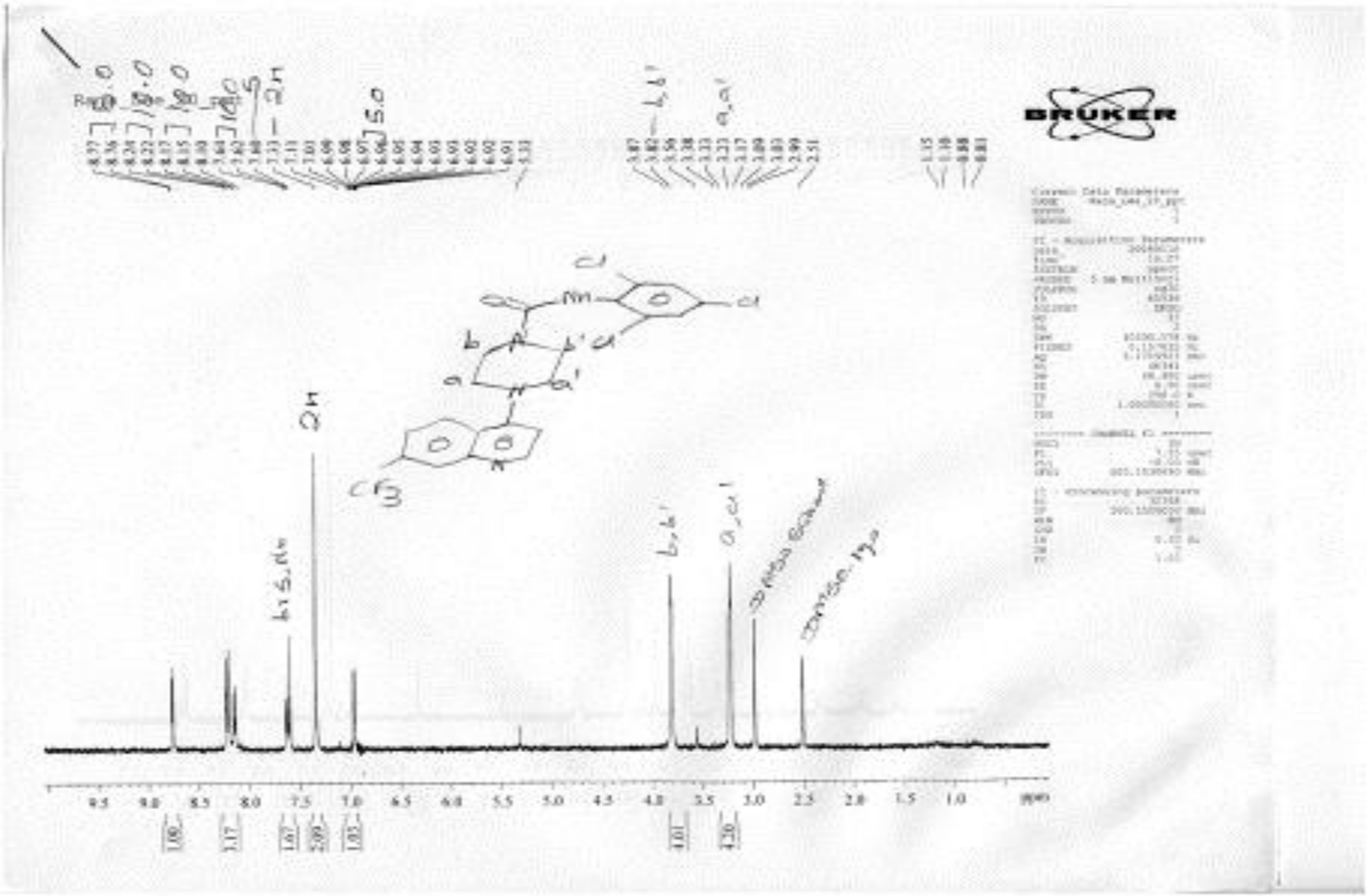

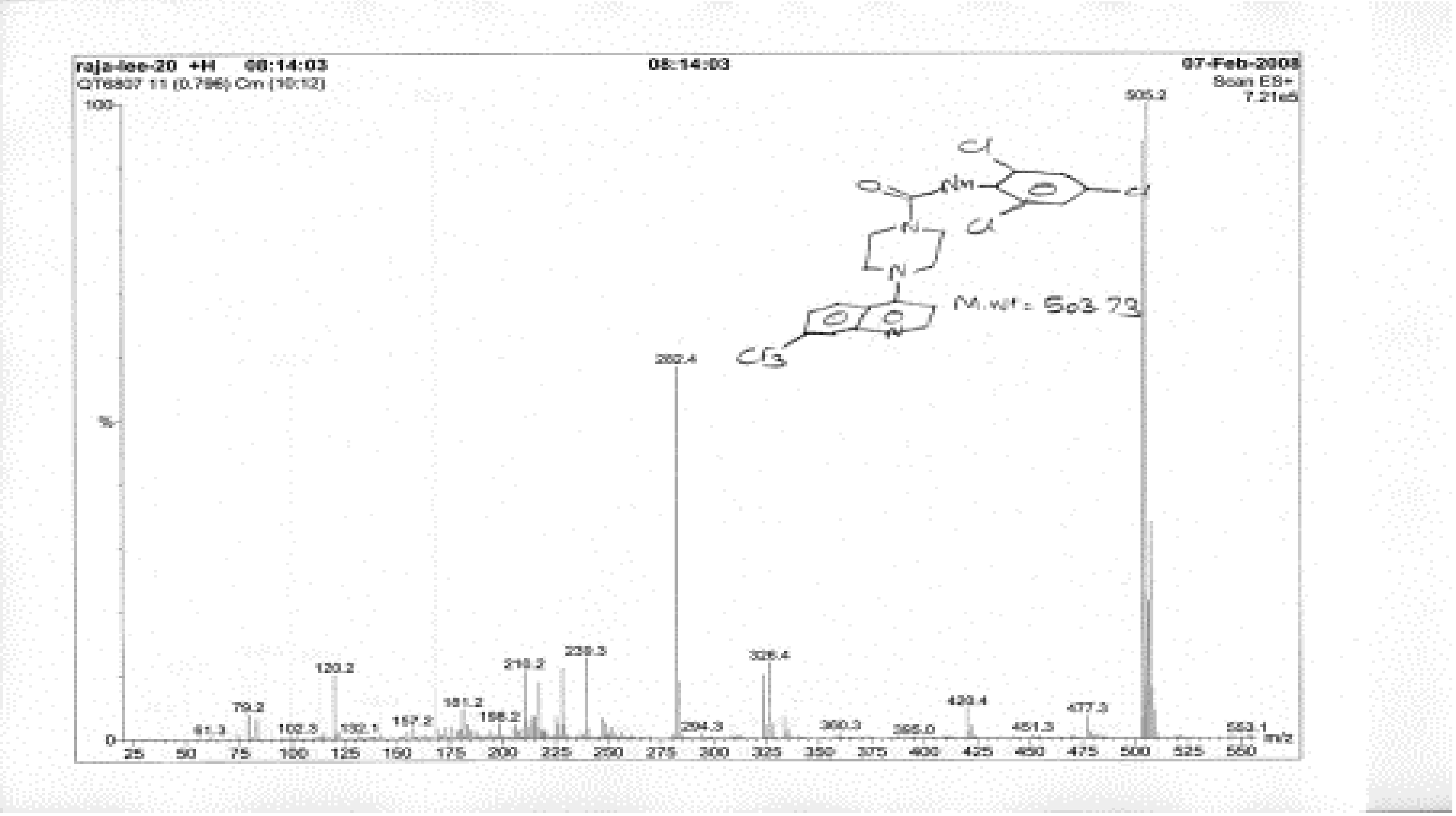

raja-lee-20 +H 08:14:03
08:14:03
07-Feb-2008
Scan ES+
7.21e5
100
%
0
505.2
282.4
239.3
326.4
120.2
210.2
79.2
181.2
420.4
477.3
157.2
198.2
61.3
102.3
132.1
294.3
360.3
396.0
451.3
553.1
m/z
M.wt = 503.73
CF3

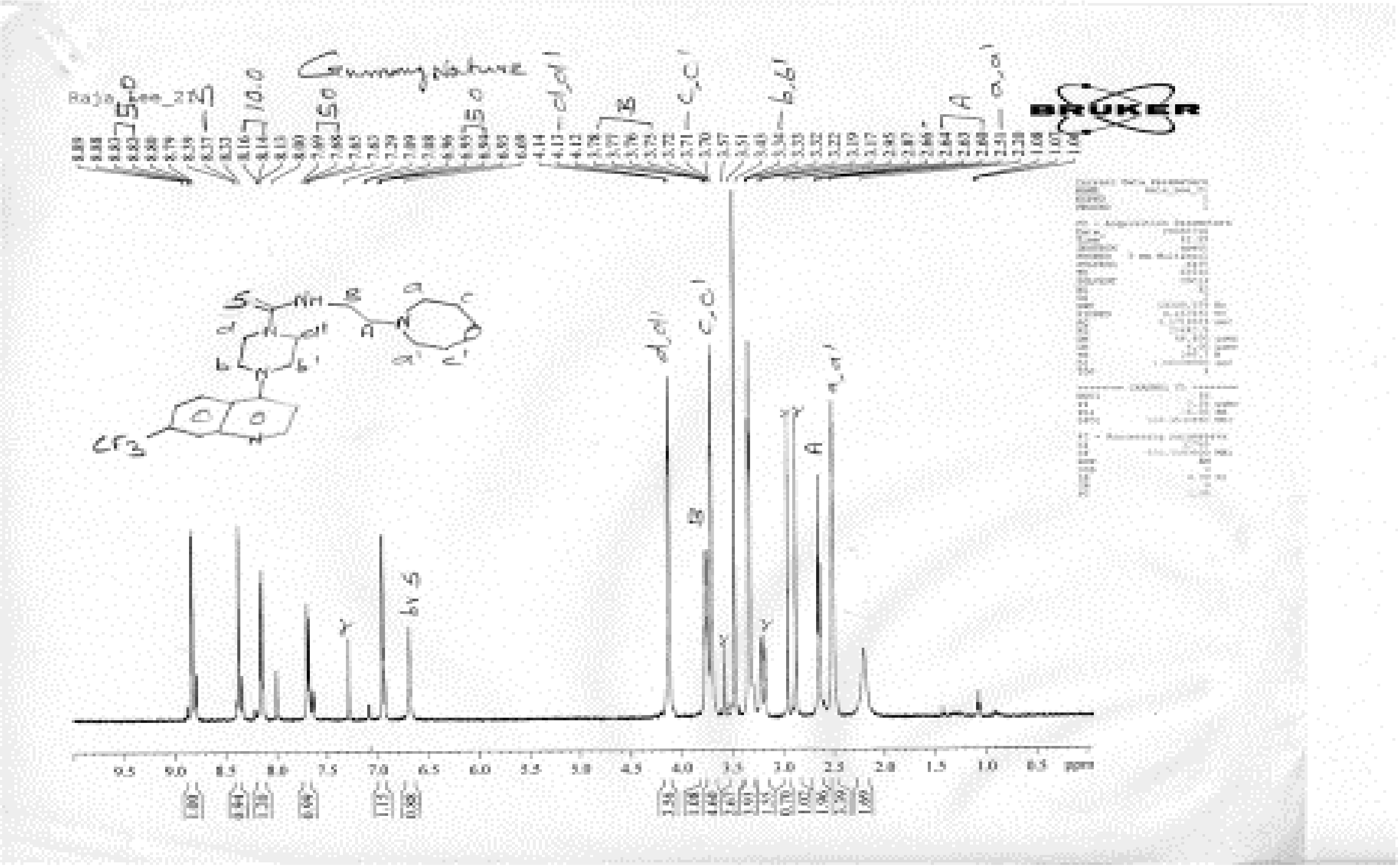

Gummy Nature
BRUKER

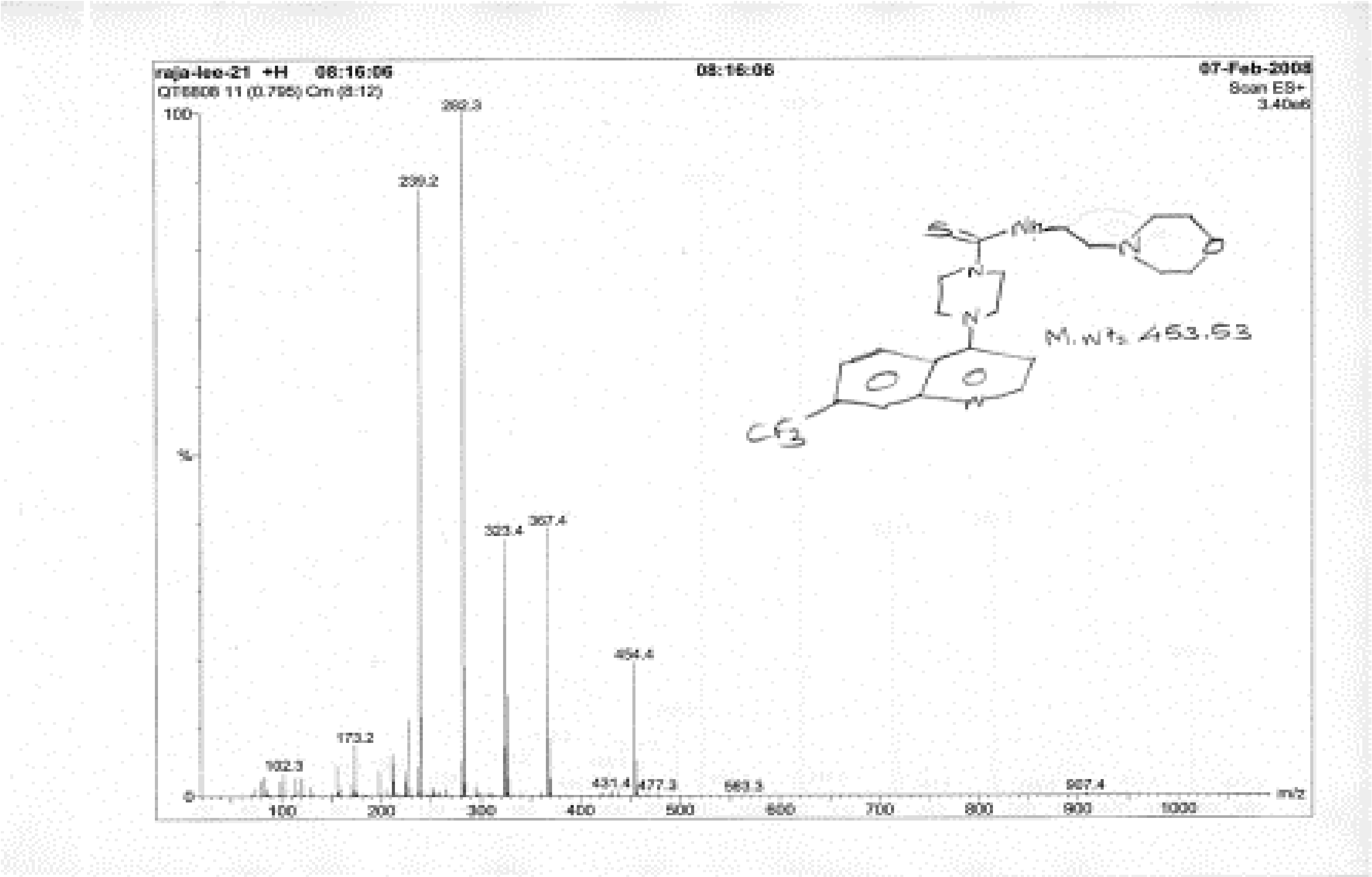
raja-lee-21 +H 08:16:06
08:16:06
07-Feb-2008
QT6808 11 (0.795) Cm (8:12)
Scan ES+
3.40e6
100
%
0
292.3
239.2
323.4
367.4
454.4
173.2
102.3
431.4
477.3
563.3
907.4
100
200
300
400
500
600
700
800
900
1000
m/z
M. Wts 453.53

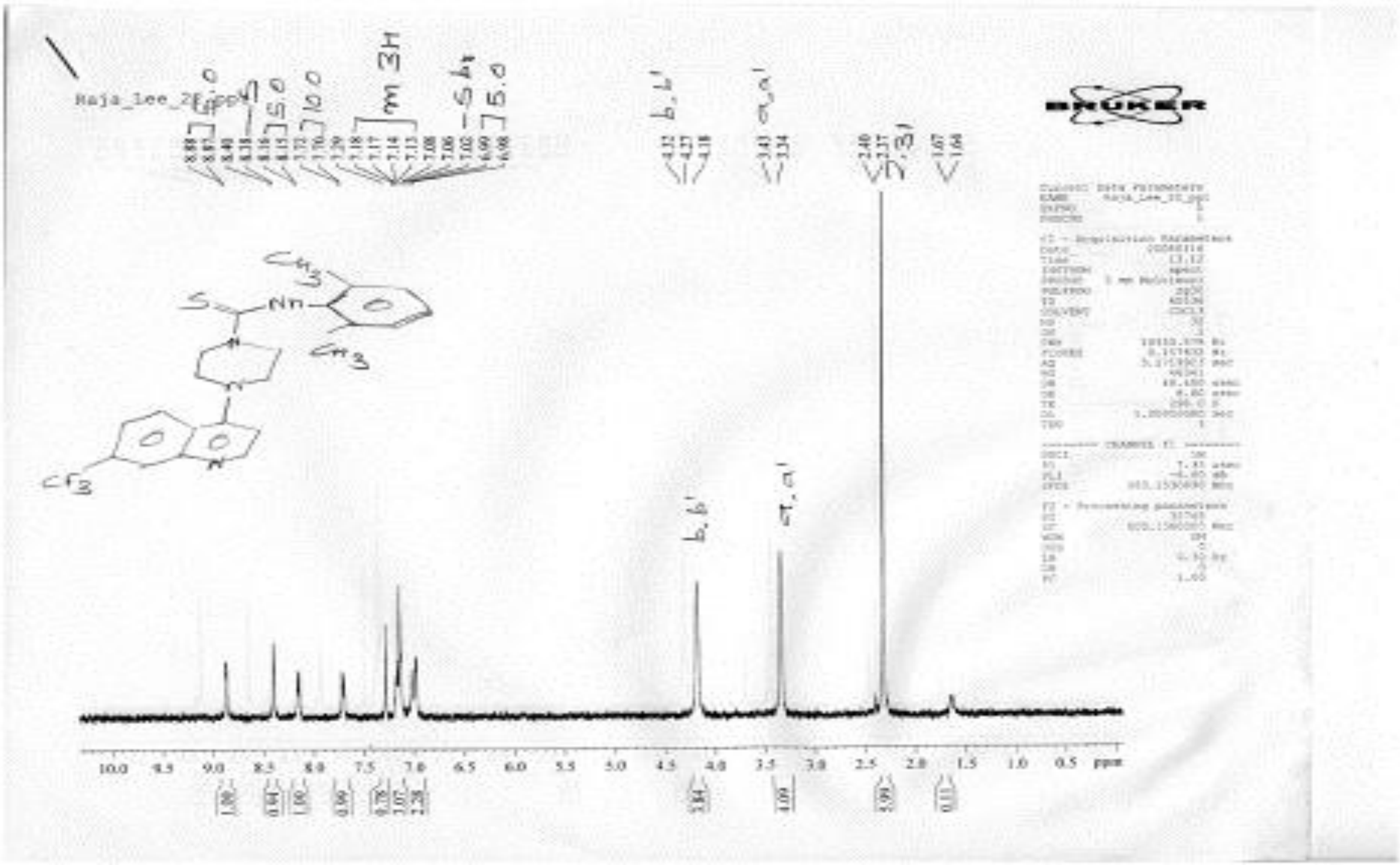

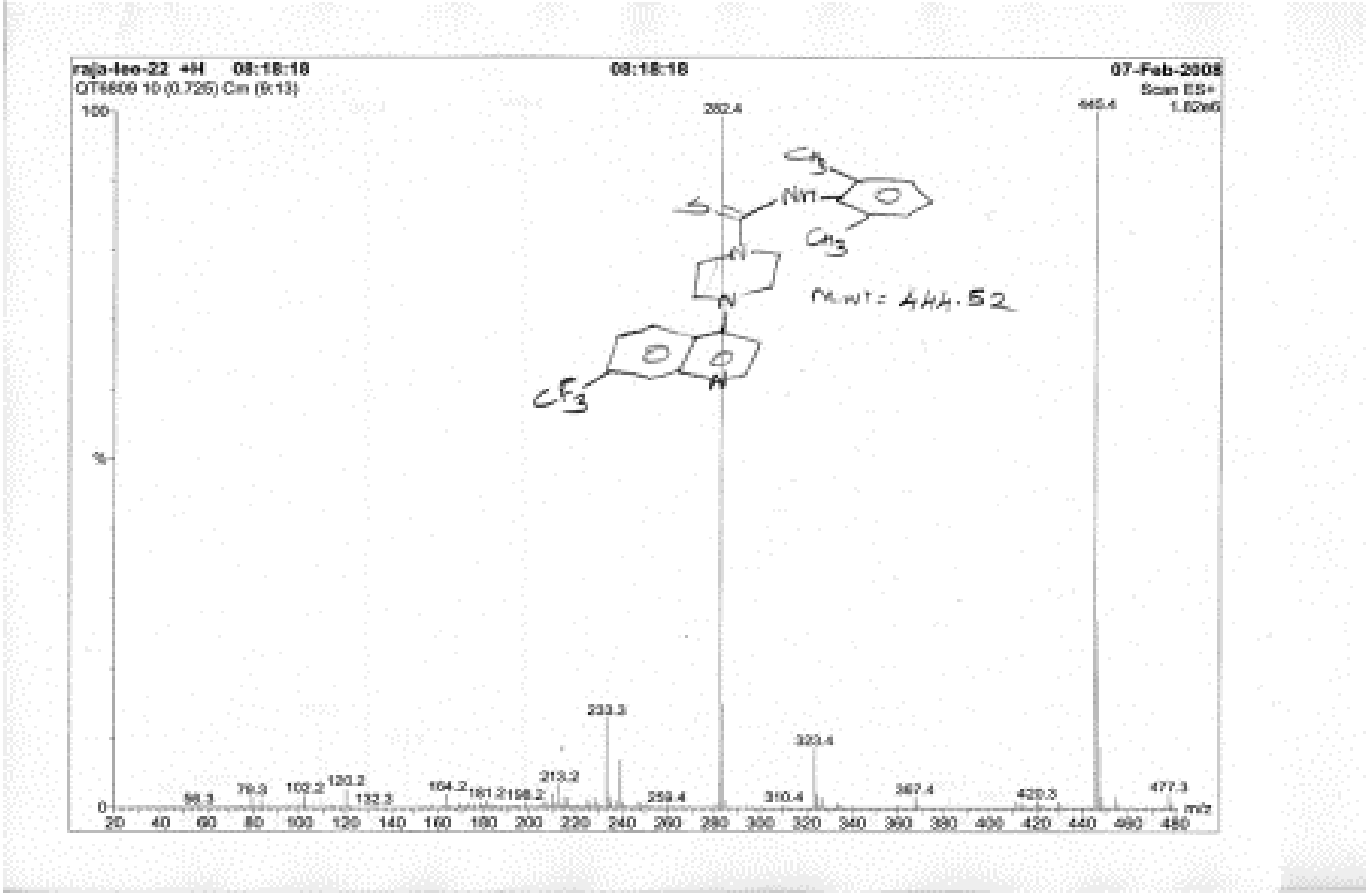
raja-leo-22 +H 08:18:18
08:18:18
07-Feb-2008
QT6809 10 (0.725) Cm (9:13)
Scan ES+
1.82e6
100
%
0
282.4
445.4
233.3
323.4
213.2
120.2
102.2
79.3
56.3
132.3
164.2
181.2
198.2
259.4
310.4
367.4
420.3
477.3
m/z
20 40 60 80 100 120 140 160 180 200 220 240 260 280 300 320 340 360 380 400 420 440 460 480
M.WT: 444.52

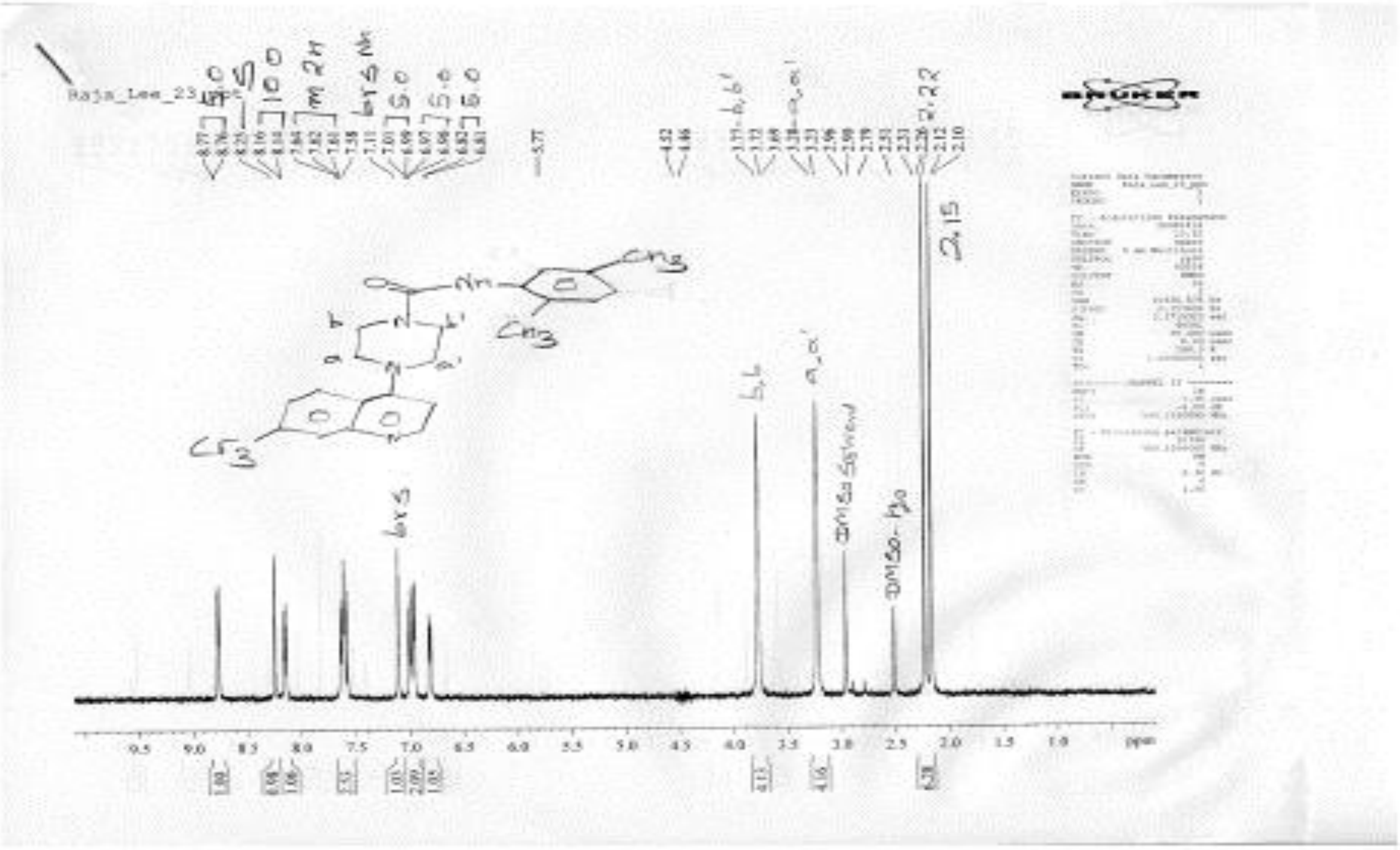
9.5 9.0 8.5 8.0 7.5 7.0 6.5 6.0 5.5 5.0 4.5 4.0 3.5 3.0 2.5 2.0 1.5 1.0 ppm

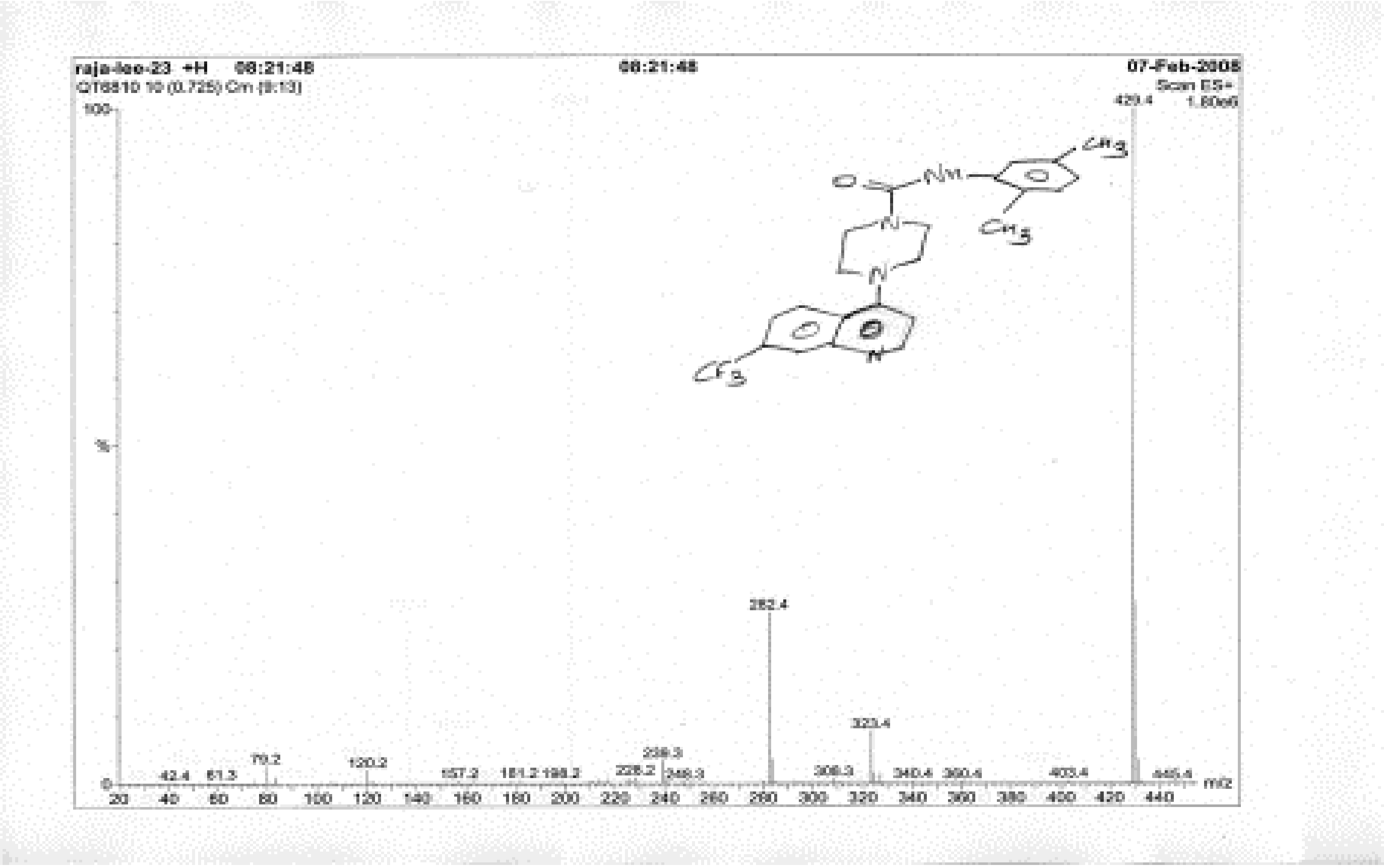

raja-lee-23 +H 08:21:48
08:21:48
07-Feb-2008
QT6810 10 (0.725) Cm (9:13)
Scan ES+
429.4
1.80e6
100
%
0
282.4
323.4
79.2
120.2
239.3
42.4
61.3
157.2
181.2
198.2
228.2
248.3
308.3
340.4
360.4
403.4
445.4
m/z

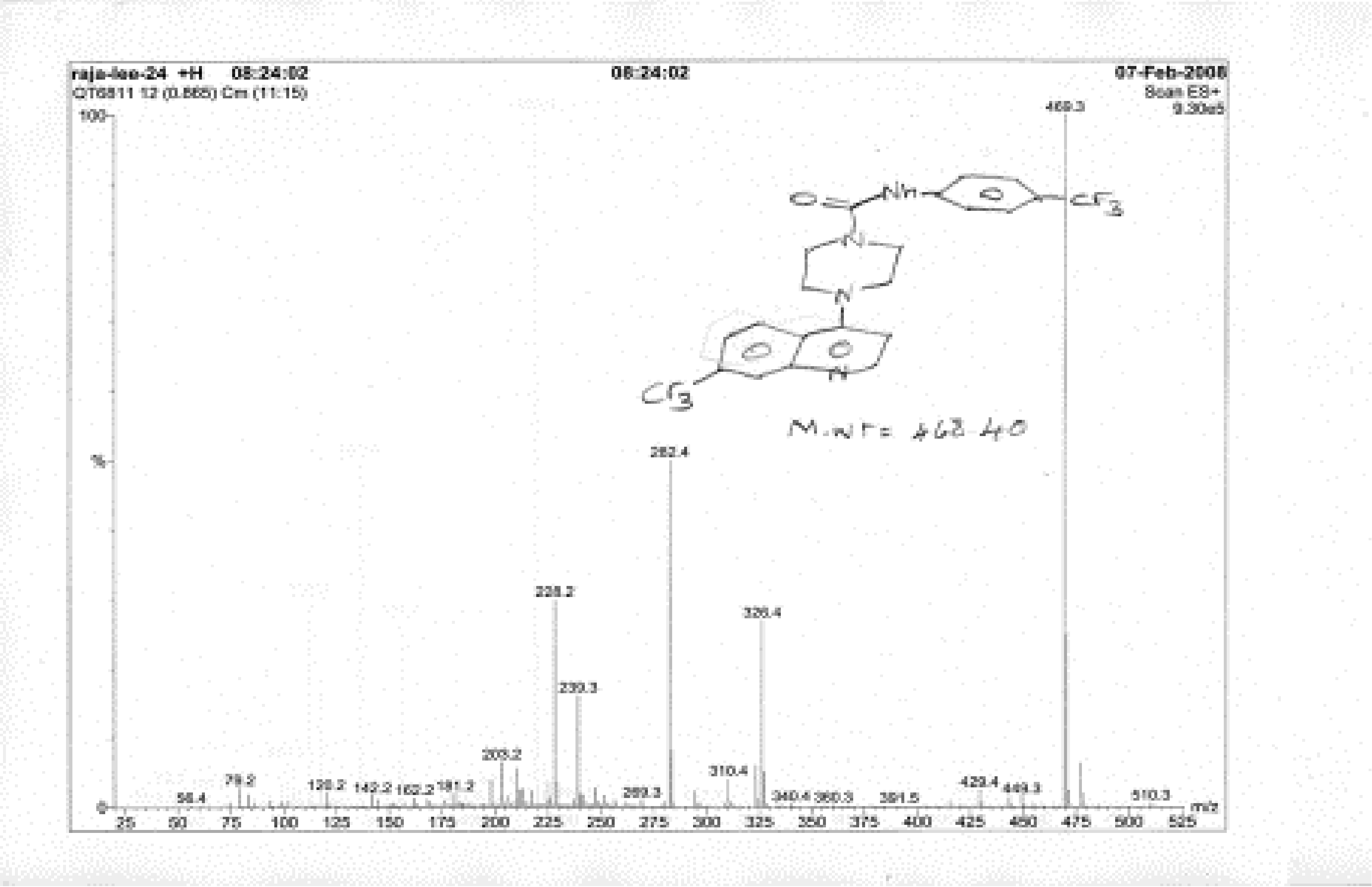
raja-lee-24 +H 08:24:02
08:24:02
07-Feb-2008
Scan ES+
M.wt= 468.40
m/z

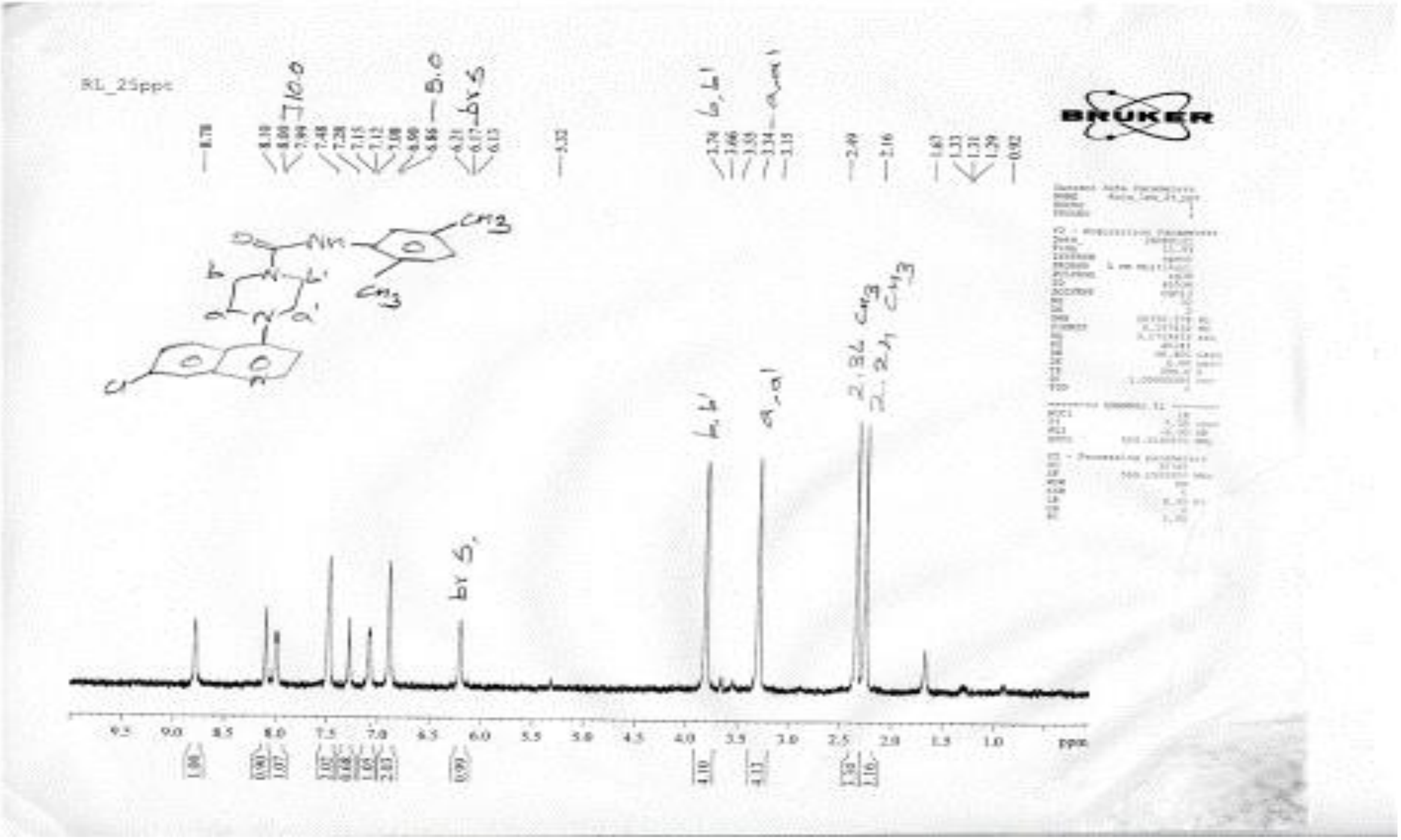

BL_25ppc
BRUKER
ppm

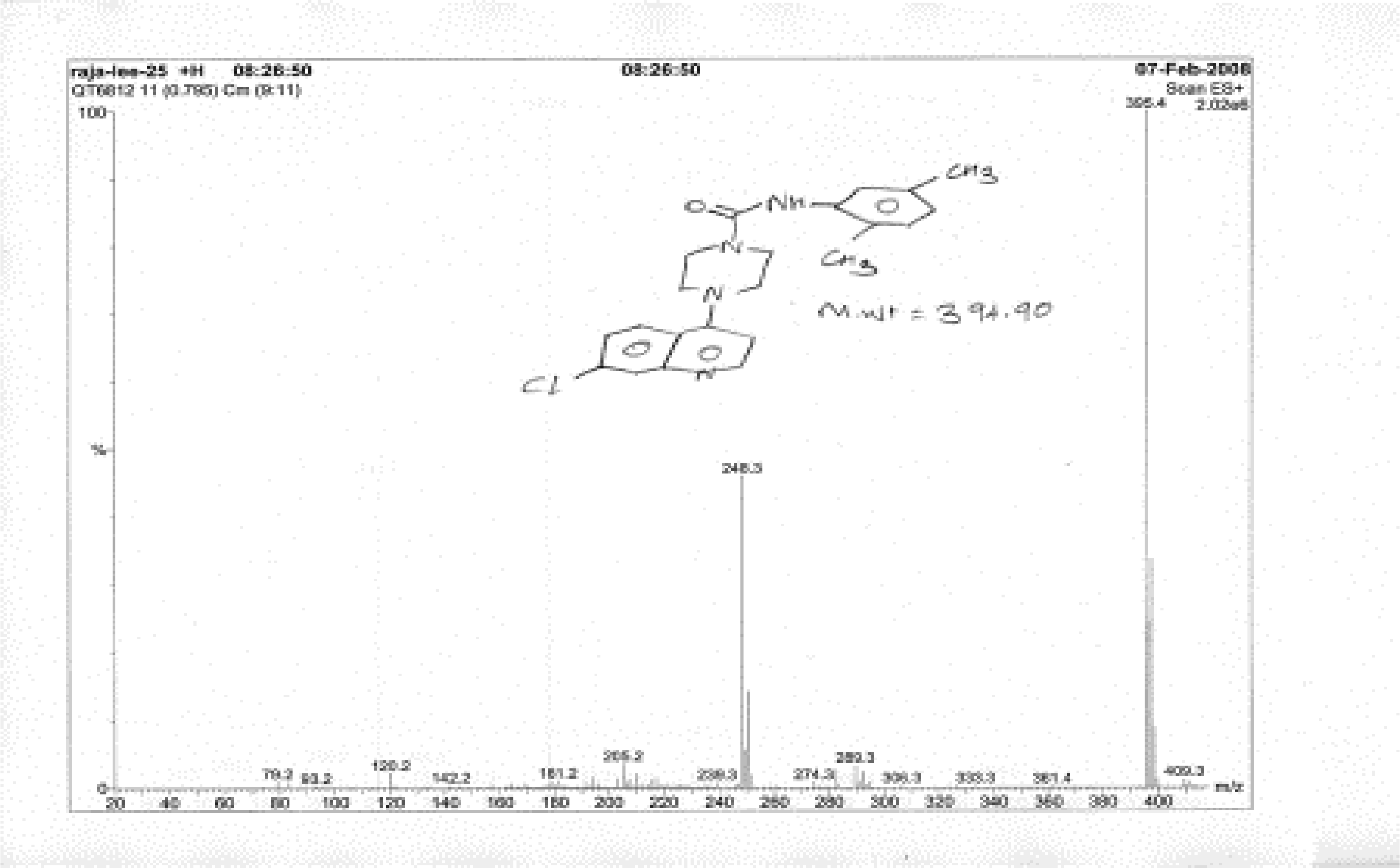

raja-lee-25 +H 08:26:50
08:26:50
07-Feb-2008
QT6812 11 (0.785) Cm (9:11)
Scan ES+
2.02e6
395.4
248.3
M.wt = 394.90
%
m/z

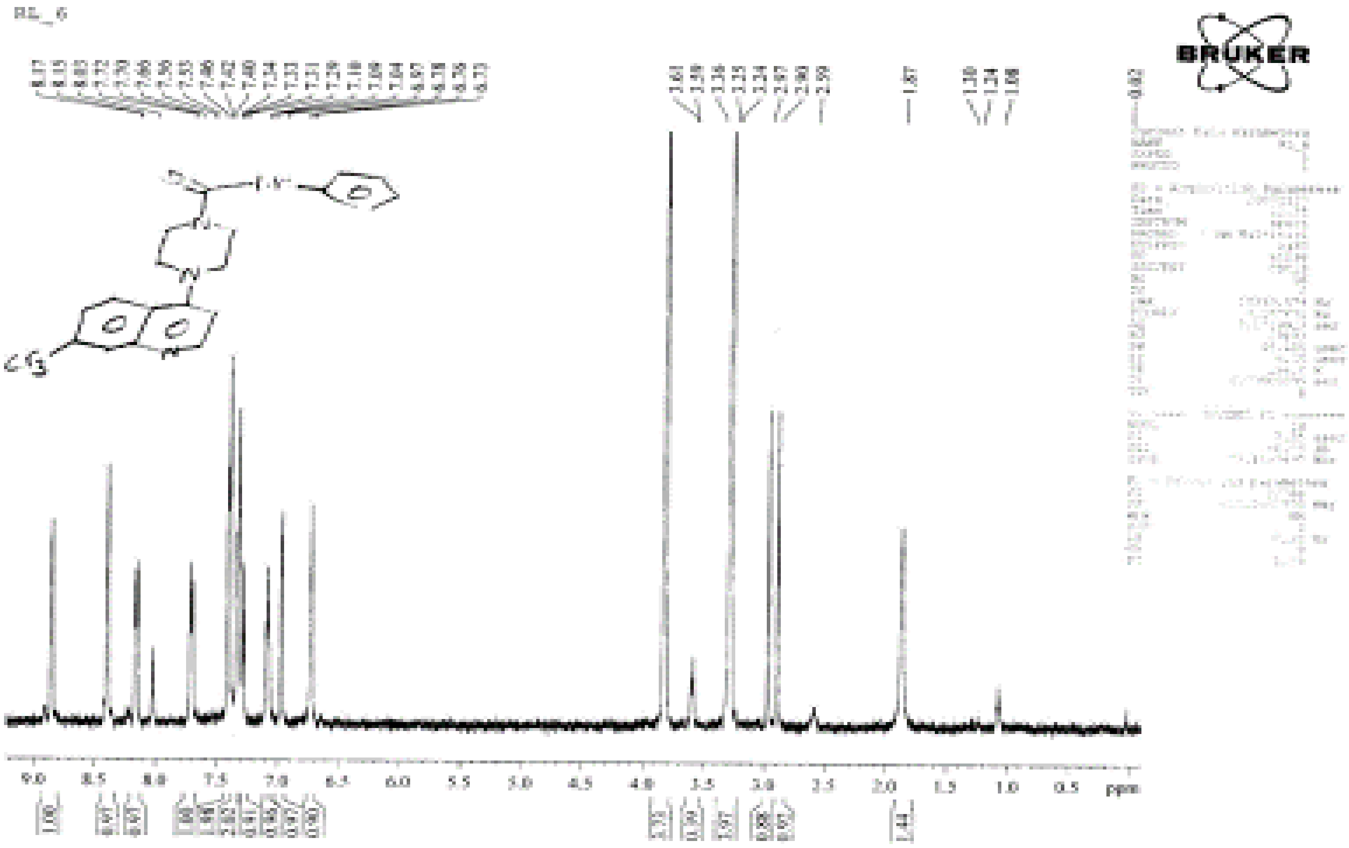
RL_6
BRUKER
9.0 8.5 8.0 7.5 7.0 6.5 6.0 5.5 5.0 4.5 4.0 3.5 3.0 2.5 2.0 1.5 1.0 0.5 ppm

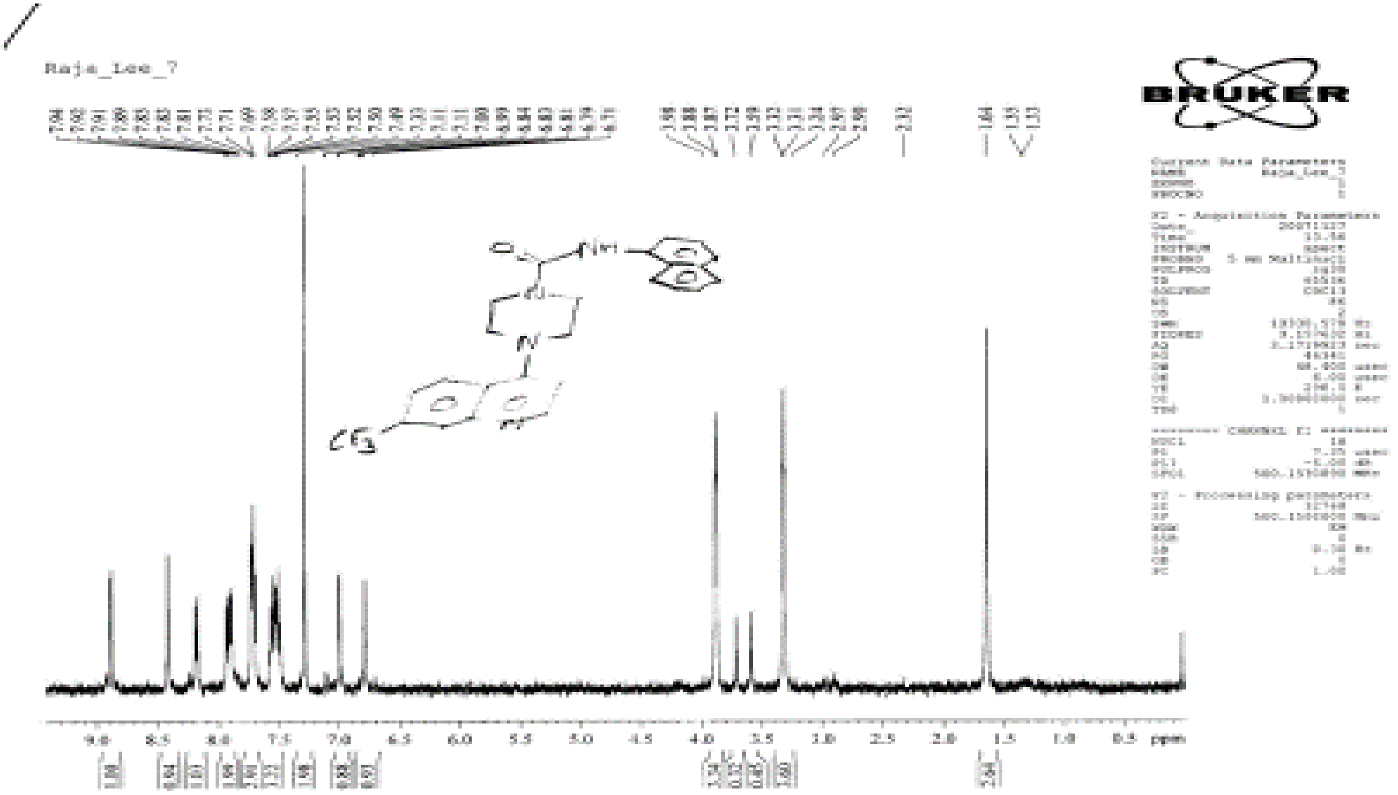
Raja_Lee_7
ppm

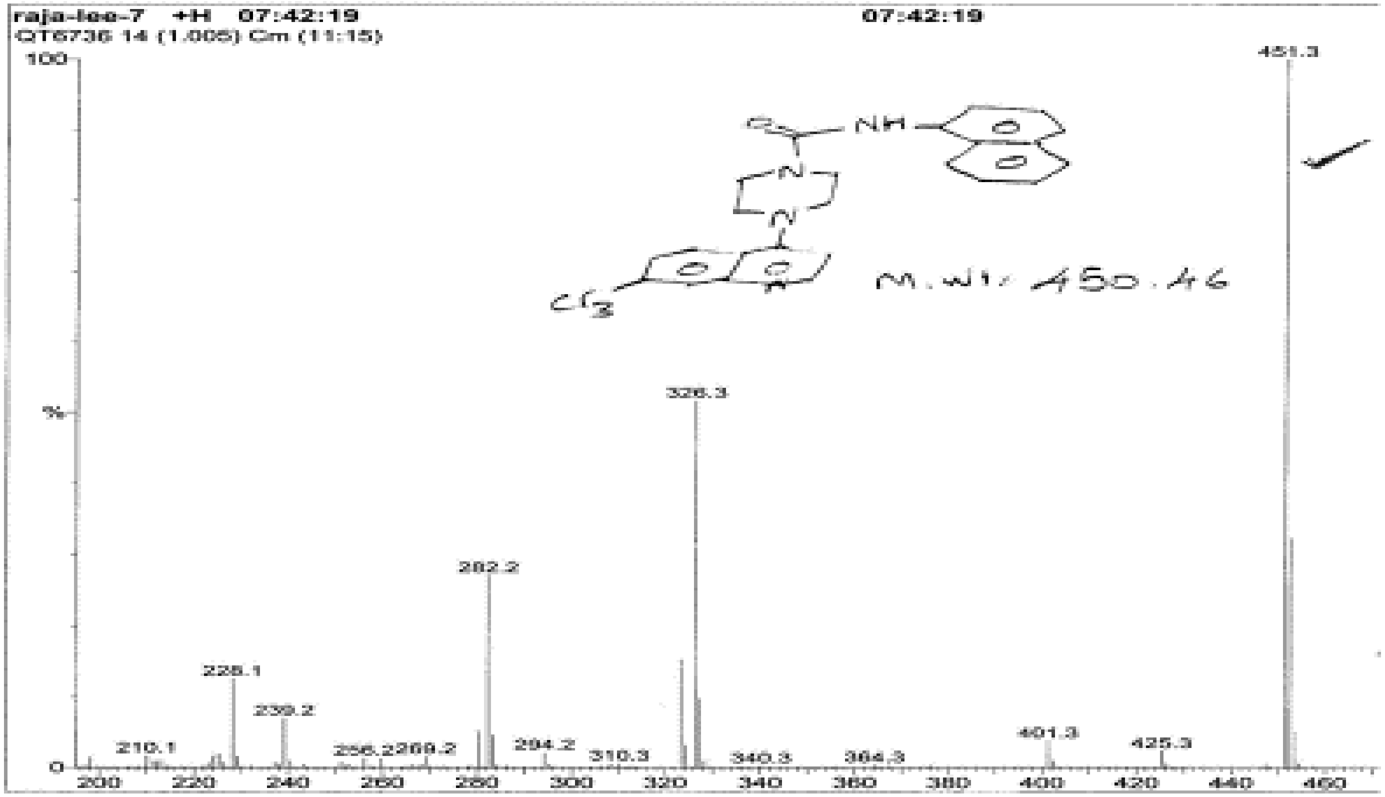
raja-lee-7 +H 07:42:19
07:42:19
QT6736 14 (1.005) Cm (11:15)
100
%
0
451.3
326.3
282.2
228.1
239.2
210.1
256.2
269.2
294.2
310.3
340.3
364.3
401.3
425.3
200
220
240
260
280
300
320
340
360
380
400
420
440
460
NH
N
M.Wt: 450.46

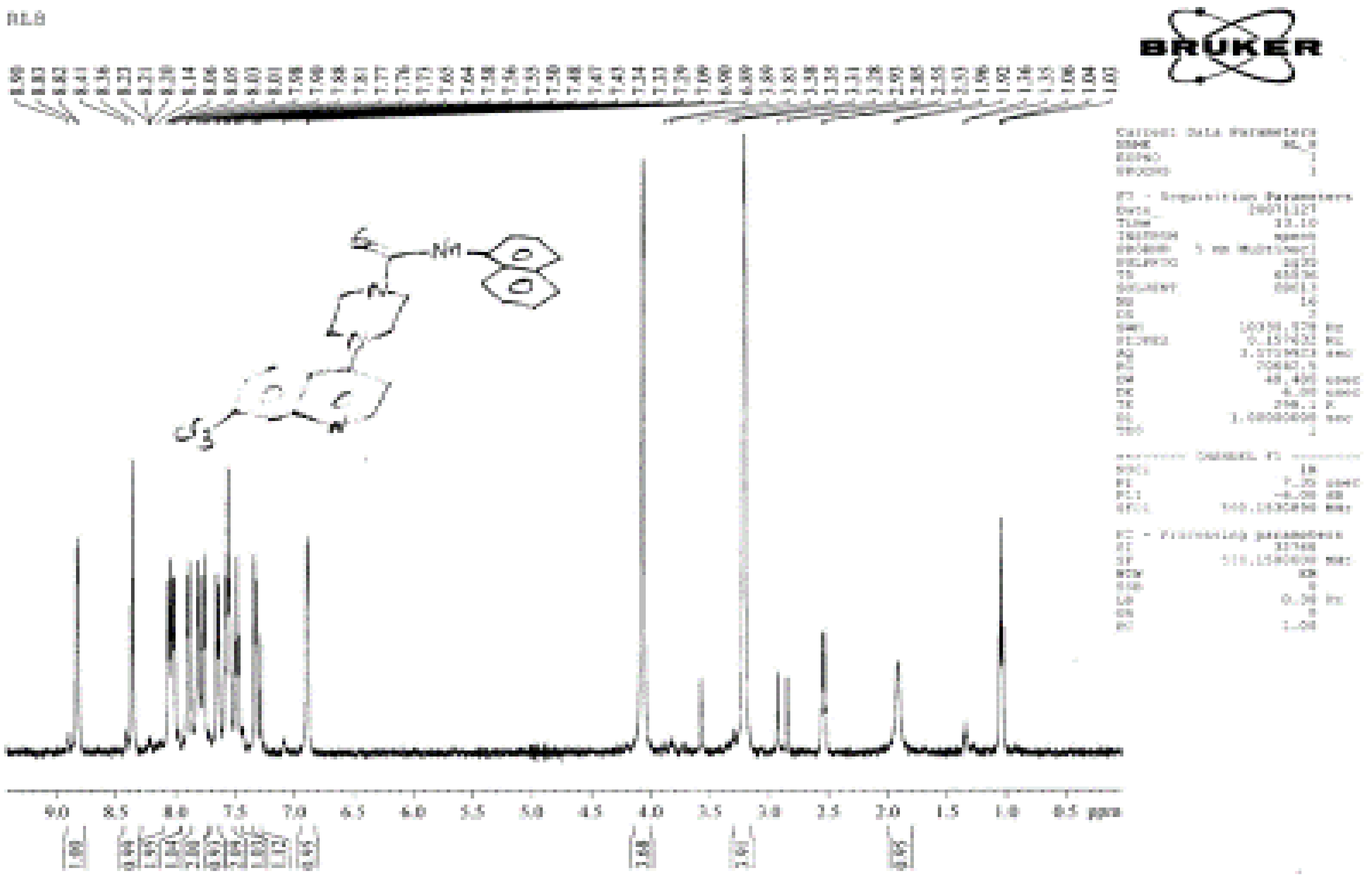
RL8
9.0 8.5 8.0 7.5 7.0 6.5 6.0 5.5 5.0 4.5 4.0 3.5 3.0 2.5 2.0 1.5 1.0 0.5 ppm

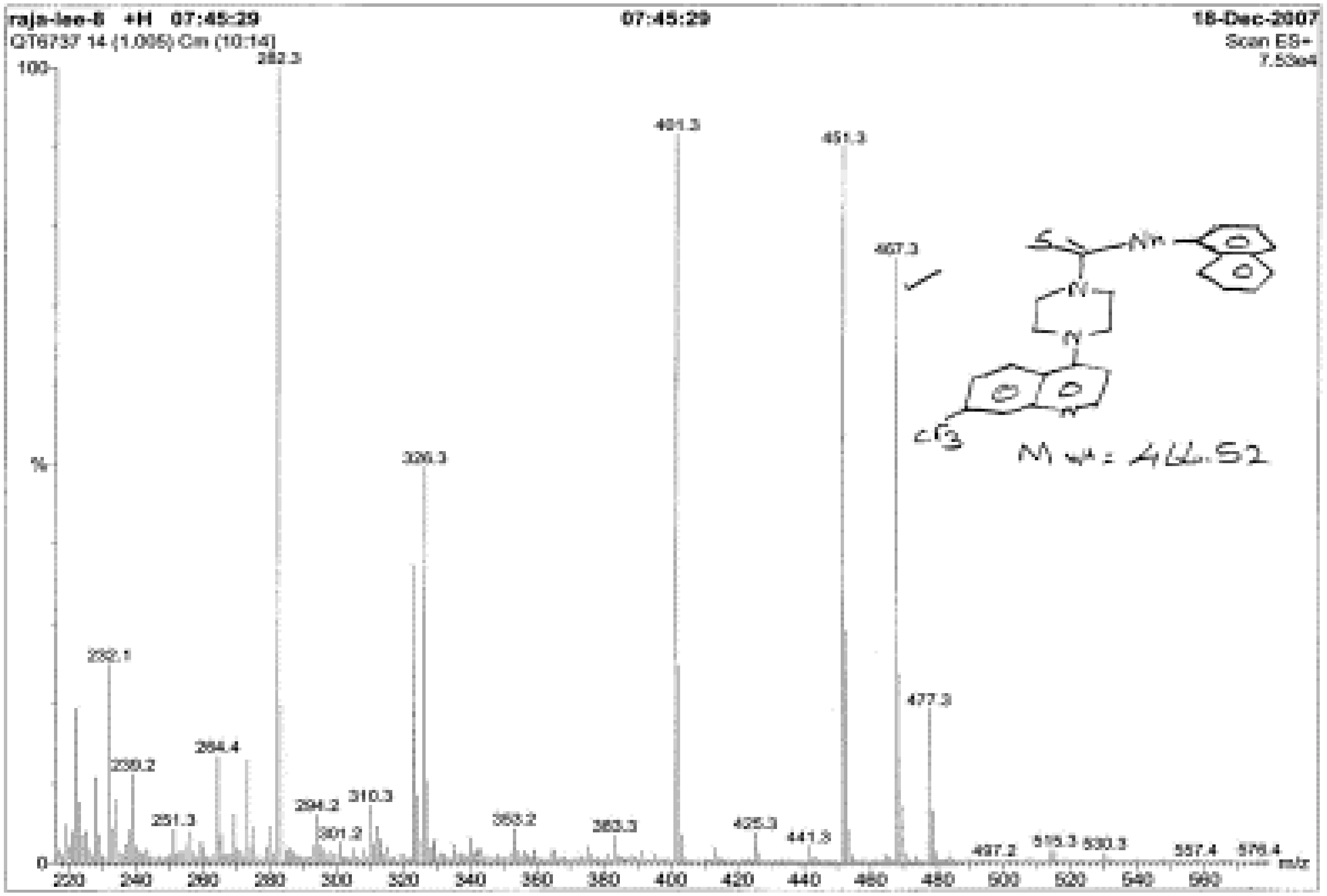
raja-lee-8 +H 07:45:29
07:45:29
18-Dec-2007
QT6737 14 (1.005) Cm (10:14)
Scan ES+
7.53e4
M wt: 466.52
m/z

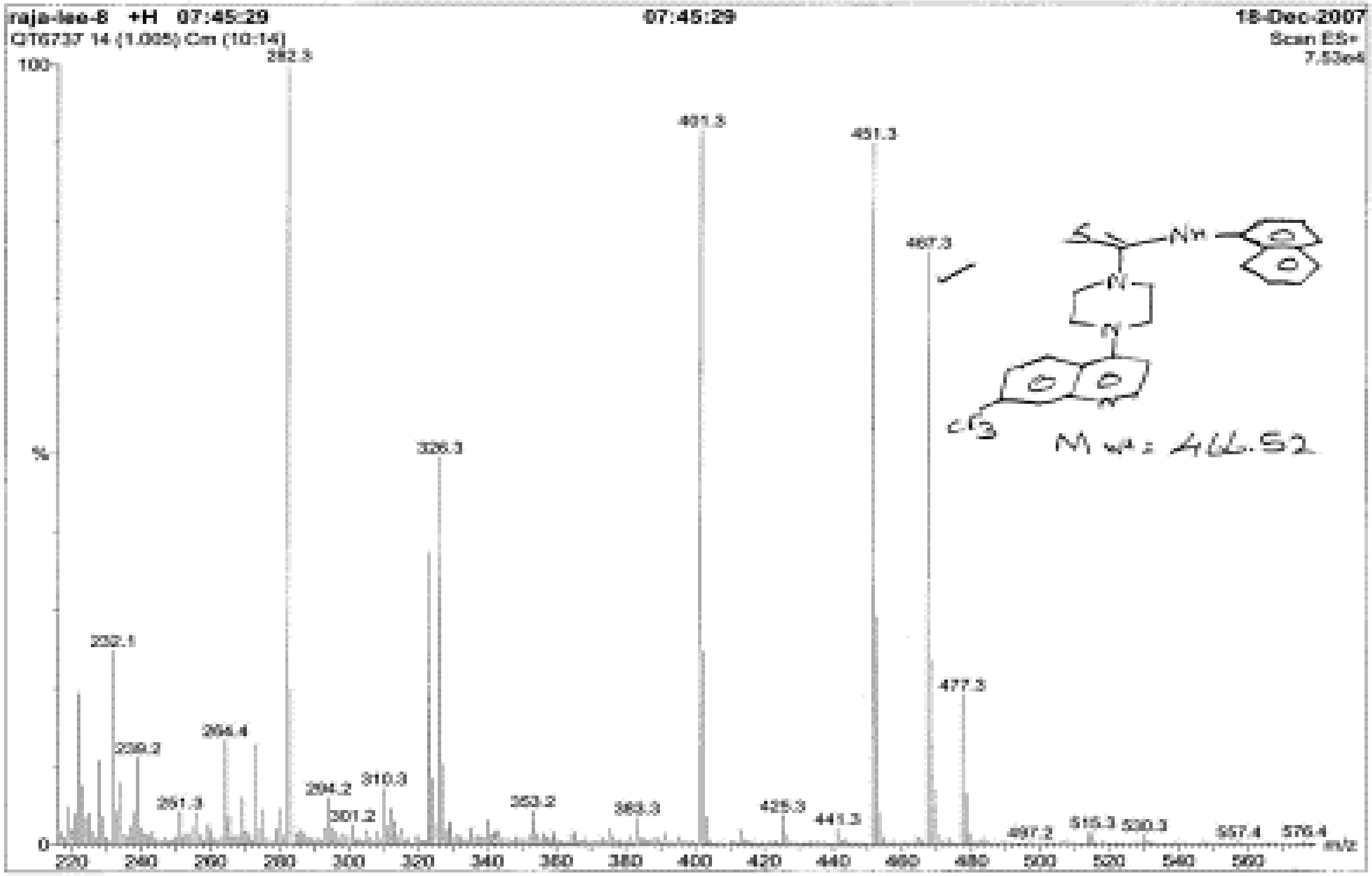
raja-lee-8 +H 07:45:29
QT6737 14 (1.005) Cm (10:14)
07:45:29
18-Dec-2007
Scan ES+
7.53e4
100
%
0
282.3
401.3
451.3
467.3
326.3
232.1
477.3
239.2
264.4
251.3
294.2
310.3
301.2
353.2
383.3
425.3
441.3
497.2
515.3
530.3
557.4
576.4
m/z
220
240
260
280
300
320
340
360
380
400
420
440
460
480
500
520
540
560

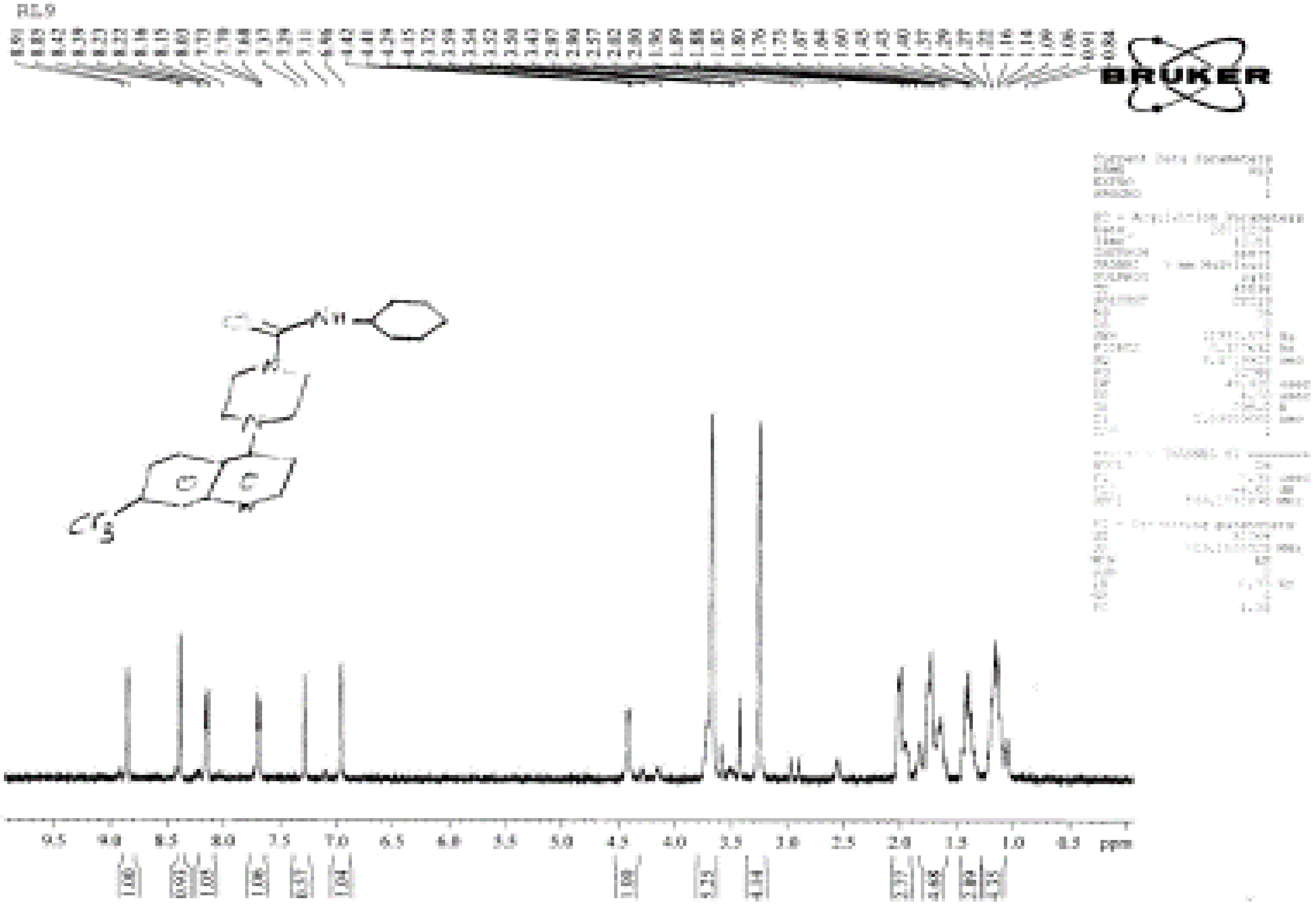
RL9
BRUKER
9.5 9.0 8.5 8.0 7.5 7.0 6.5 6.0 5.5 5.0 4.5 4.0 3.5 3.0 2.5 2.0 1.5 1.0 0.5 ppm

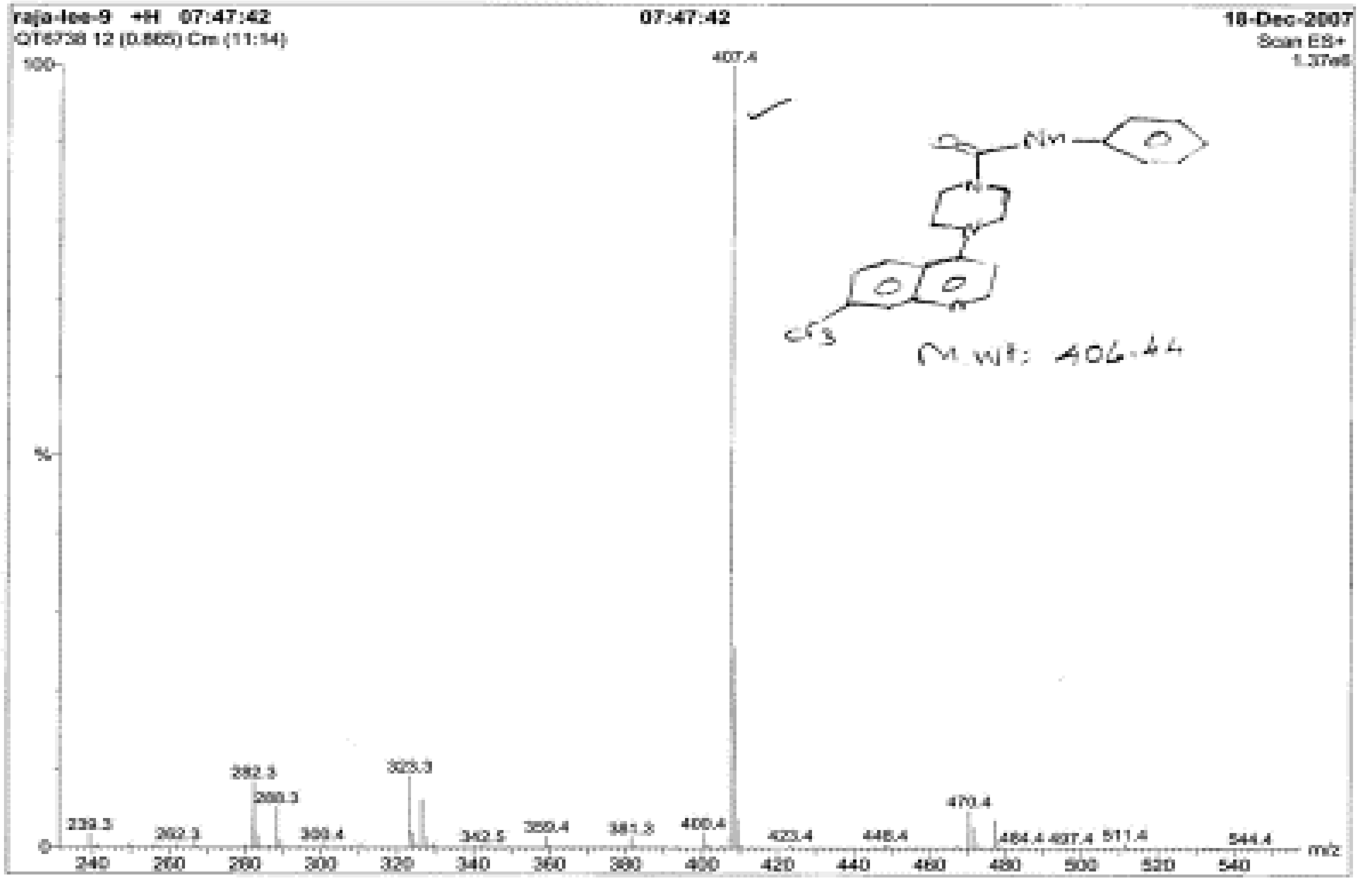
raja-lee-9 +H 07:47:42
07:47:42
18-Dec-2007
Scan ES+
407.4
M. wt: 406.44
m/z

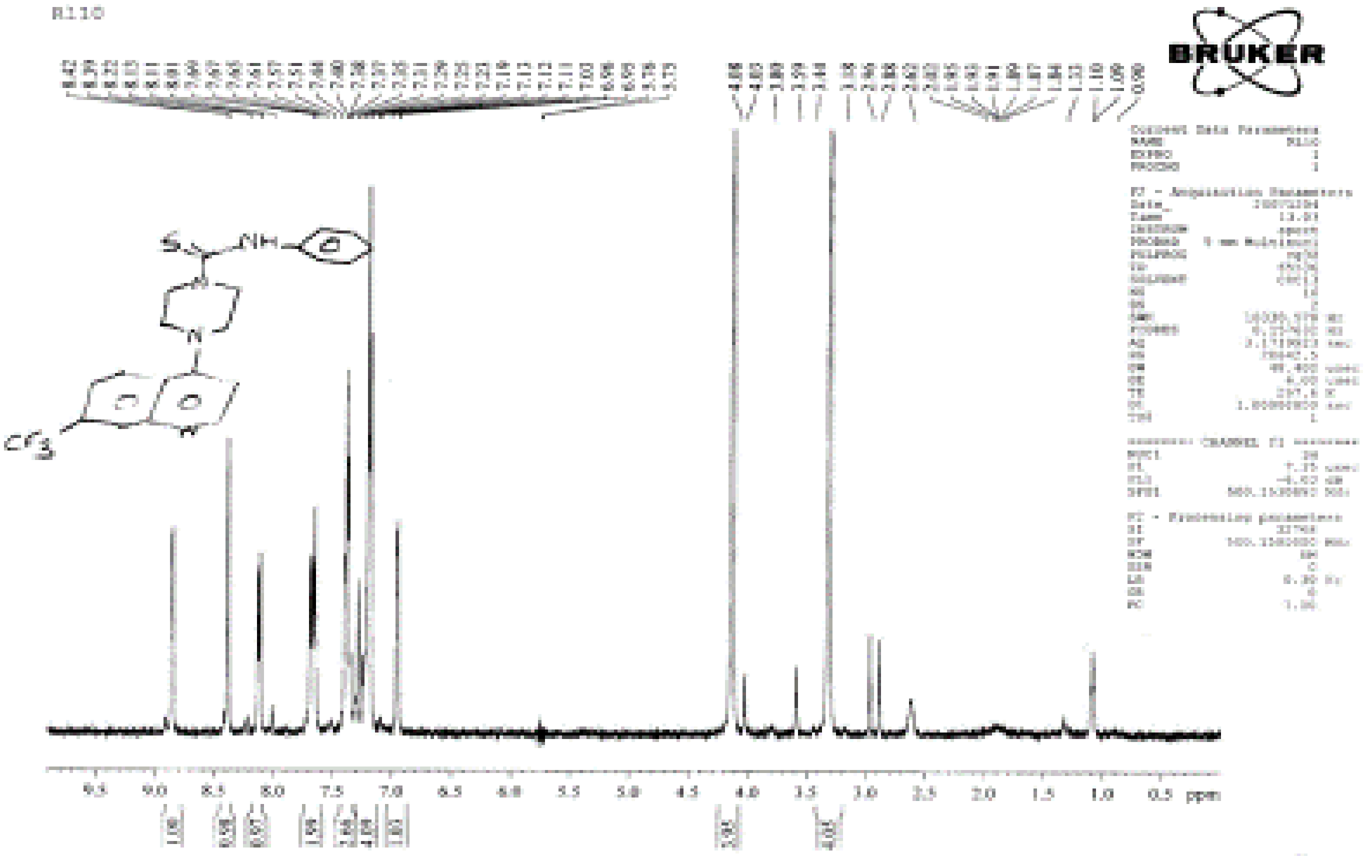
R110
BRUKER
9.5 9.0 8.5 8.0 7.5 7.0 6.5 6.0 5.5 5.0 4.5 4.0 3.5 3.0 2.5 2.0 1.5 1.0 0.5 ppm

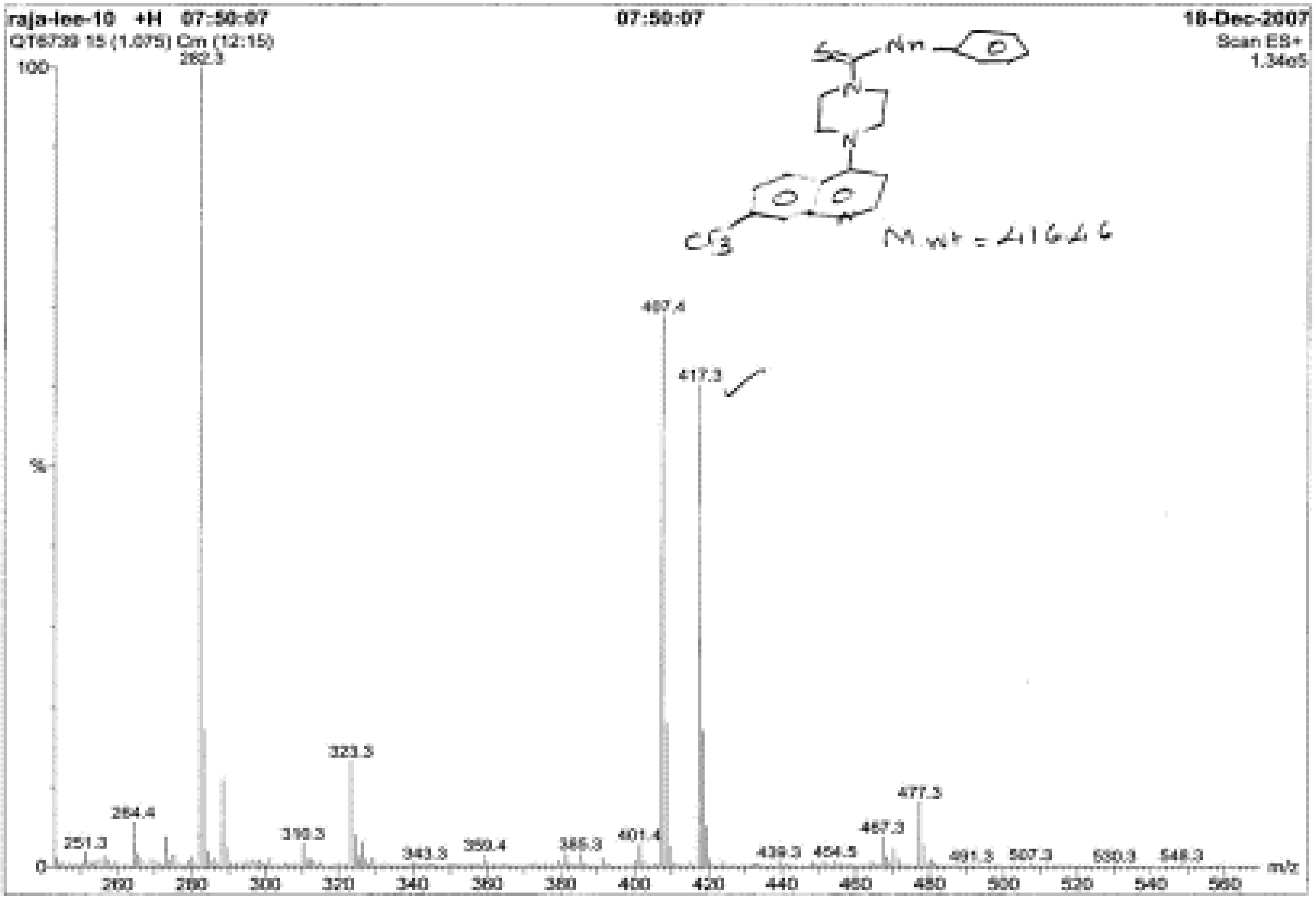
raja-lee-10 +H 07:50:07
QT6739 15 (1.075) Cm (12:15)
07:50:07
18-Dec-2007
Scan ES+
1.34e5
M.wt = 416.46
100
%
0
282.3
407.4
417.3
323.3
477.3
264.4
251.3
310.3
343.3
359.4
385.3
401.4
439.3
454.5
467.3
491.5
507.3
530.3
548.3
m/z
